# Targeting Endothelial KDM5A to Attenuate Aging and Ameliorate Age‐Associated Metabolic Abnormalities

**DOI:** 10.1002/advs.202512657

**Published:** 2025-11-14

**Authors:** Rifeng Gao, Lifeng Liang, Ling Yang, Chunyu Lyu, Yang Lyu, Weijun Yang, Jiaran Shi, Wei Wei, Jiahui Cheng, Xiaolei Sun, Xian Zhu, Chao Chen, Xiaoting Xu, Jianchuang Qi, Wenli Li, Yizhe Zhang, Xiao Zhang, Yan Zhou, Aiqiang Dong, Juntao Chen, Bo Li, Kun Yang

**Affiliations:** ^1^ Department of Cardiac Surgery, The Second Affiliated Hospital Zhejiang University Hangzhou 310009 China; ^2^ Department of Cardiology, Cardiovascular Center, Beijing Friendship Hospital Capital Medical University Beijing 100029 China; ^3^ Department of Ultasonography, Shanghai Chest Hospital Shanghai Jiao Tong University School of Medicine Shanghai 200127 China; ^4^ School of Nursing Zhejiang Chinese Medical University Hangzhou 310053 China; ^5^ Department of Cardiology, Shanghai Fifth People's Hospital Fudan University Shanghai 200240 China; ^6^ Department of Cardiology Lihuili Hospital Facilitated to Ningbo University Ningbo 315048 China; ^7^ Department of Radiology Renji Hospital Affiliated to Shanghai Jiao Tong University School of Medicine Shanghai 200127 China; ^8^ Department of Cardiology, Shanghai Institute of Cardiovascular Diseases, Zhongshan Hospital Fudan University Shanghai 200232 China; ^9^ Department of Anesthesiology Renji Hospital Affiliated to Shanghai Jiao Tong University School of Medicine Shanghai 200127 China; ^10^ Key Laboratory of Anesthesiology (Shanghai Jiao Tong University) Ministry of Education Shanghai 200127 China; ^11^ Department of Urology, The Second Affiliated Hospital Zhejiang University Hangzhou 310009 China

**Keywords:** FABP4, KDM5A, aging, metabolic abnormalities, vascular endothelial cells

## Abstract

Vascular aging accelerates the gradual deterioration of systemic organ function, yet its key driving factors are still largely unexplored. Here, it is demonstrated that lysine‐specific demethylase 5A (KDM5A) decreases and histone H3 lysine 4 (H3K4me3) increases in vascular endothelial cells (VECs) isolated from ageing mice and VEC senescence models. KDM5A deficiency exacerbated endothelial cell aging in vitro. Endothelial‐specific KDM5A‐deficient mice exhibit shortened lifespan and multiple senescent phenotypes, including fat accumulation, reduced thermogenic capacity, skeletal kyphosis, and age‐related liver lesions, while maintaining VECs‐specific KDM5A levels attenuates these adverse metabolic abnormalities and prolongs lifespan. Mechanistically, endothelial KDM5A deficiency aggravates aging‐associated fatty acid (FA) metabolism disorders by enhancing H3K4me3 enrichment at the promoter region of FA‐binding protein 4 (*FABP4*), which leads to active *FABP4* transcription. Together, the study reveals the regulatory mechanisms of KDM5A in age‐dependent metabolic disorders and identifies KDM5A/FABP4 axis as a potential therapeutic target for vascular aging and related organ dysfunction.

## Graphical abstract

This study identifies endothelial KDM5A as a key regulator of aging. KDM5A deficiency accelerates aging by enhancing H3K4me3‐mediated FABP4 expression, disrupting fatty acid metabolism, and promoting multi‐organ senescence. KDM5A restoration or FABP4 inhibition reverses these adverse effects and extends lifespan, positioning the KDM5A/FABP4 axis as a potential therapeutic target for vascular aging and metabolic disorders.

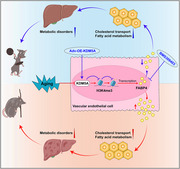

## 1 Introduction

Age‐related decline in vascular function affects organ physiology, prompting the “vascular senescence theory”, which states that vascular senescence is a prior driver of aging and suggests vascular senescence as a highly graded element during organ aging.^[^ ^1^, ^2^ ^]^ Previously, a few animal studies have demonstrated the alleviation of age‐associated pathologies by vascular manipulation.^[^ ^3^, ^4^, ^5^, ^6^ ^]^ Endothelial‐derived paracrine factors are crucial for maintaining the physiological function of organs,^[^ ^7^, ^8^ ^]^ and the dysfunction of endothelial secretion is a sign of an aging circulatory system,^[^ ^9^ ^]^ which may induce major age‐related pathologies. This age‐related paracrine dysfunction of vascular endothelial cells (VECs) leads to physiological aging manifestations of multiple organ systems,^[^ ^1^, ^10^, ^11^, ^12^, ^13^ ^]^ and improving the paracrine function of VECs can enhance organ function and extend lifespan.^[^ ^1^, ^12^, ^13^ ^]^ Therefore, preventing and intervening vascular senescence is crucial for maintaining organ function, delaying age‐related metabolic disorders, and extending lifespan.

Aging is typically characterized as a decline in bodily function accompanied by systemic metabolic dysfunction, with severe disruptions in fatty acid (FA) metabolism processes associated with vascular aging.^[^ ^9^, ^14^, ^15^ ^]^ For instance, AMPK, an essential kinase of the nutrient‐sensing signaling pathways in longevity, inhibits FA synthesis and promotes FA oxidation via inhibition of acetyl‐CoA carboxylase.^[^ ^16^, ^17^ ^]^ Moreover, vascular aging triggers metabolic diseases such as dyslipidemia, hypertension, obesity, and metabolic syndrome.^[^ ^18^, ^19^, ^20^ ^]^ The metabolic disorders in the aging liver may contribute to metabolic dysfunction and increased susceptibility to age‐related diseases.^[^ ^21^ ^]^ Current reports highlight the role of Liver FA oxidation in aging and longevity. Ketogenic diet specifically reduces midlife mortality by upregulating the genes involved in FA oxidation in the liver.^[^ ^22^ ^]^ Differential Rank Conservation analyses of mouse liver proteomics and transcriptomics data show that lifespan‐extending interventions generally tighten the regulation of biological modules, including FA oxidation.^[^ ^23^ ^]^

Epigenetic mechanisms, particularly post‐translational modifications of histones,^[^ ^24^ ^]^ integrate environmental signals to influence gene expression and downstream cellular processes.^[^ ^25^, ^26^, ^27^ ^]^ An increase in histone H3 lysine 4 (H3K4me3) expression leads to age‐related diseases and shorter lifespan.^[^ ^28^, ^29^, ^30^, ^31^ ^]^ Lysine‐specific demethylase 5A (KDM5A) is a member of the Jumonji C domain‐containing histone demethylase family,^[^ ^32^, ^33^ ^]^ which demethylates histones and removes methyl groups specifically from H3K4me3.^[^ ^2^, ^29^, ^34^, ^35^ ^]^ Notably, KDM5A is involved in the regulation of DNA repair,^[^ ^36^ ^]^ progression of the cell cycle,^[^ ^37^ ^]^ cellular aging,^[^ ^29^ ^]^ and other processes.^[^ ^35^, ^38^ ^]^ However, the role of KDM5A in VECs, particularly in endothelial aging, remains to be elucidated.

In this study, we investigated whether KDM5A‐mediated modification of histone demethylation of H3K4me3 in VECs could provide comprehensive protection against aging, and the underlying mechanisms by which endothelial KDM5A regulates aging‐related metabolism.

## 2 Results

### 2.1 KDM5A Decreases in Senescent VECs

**Figure 1:**
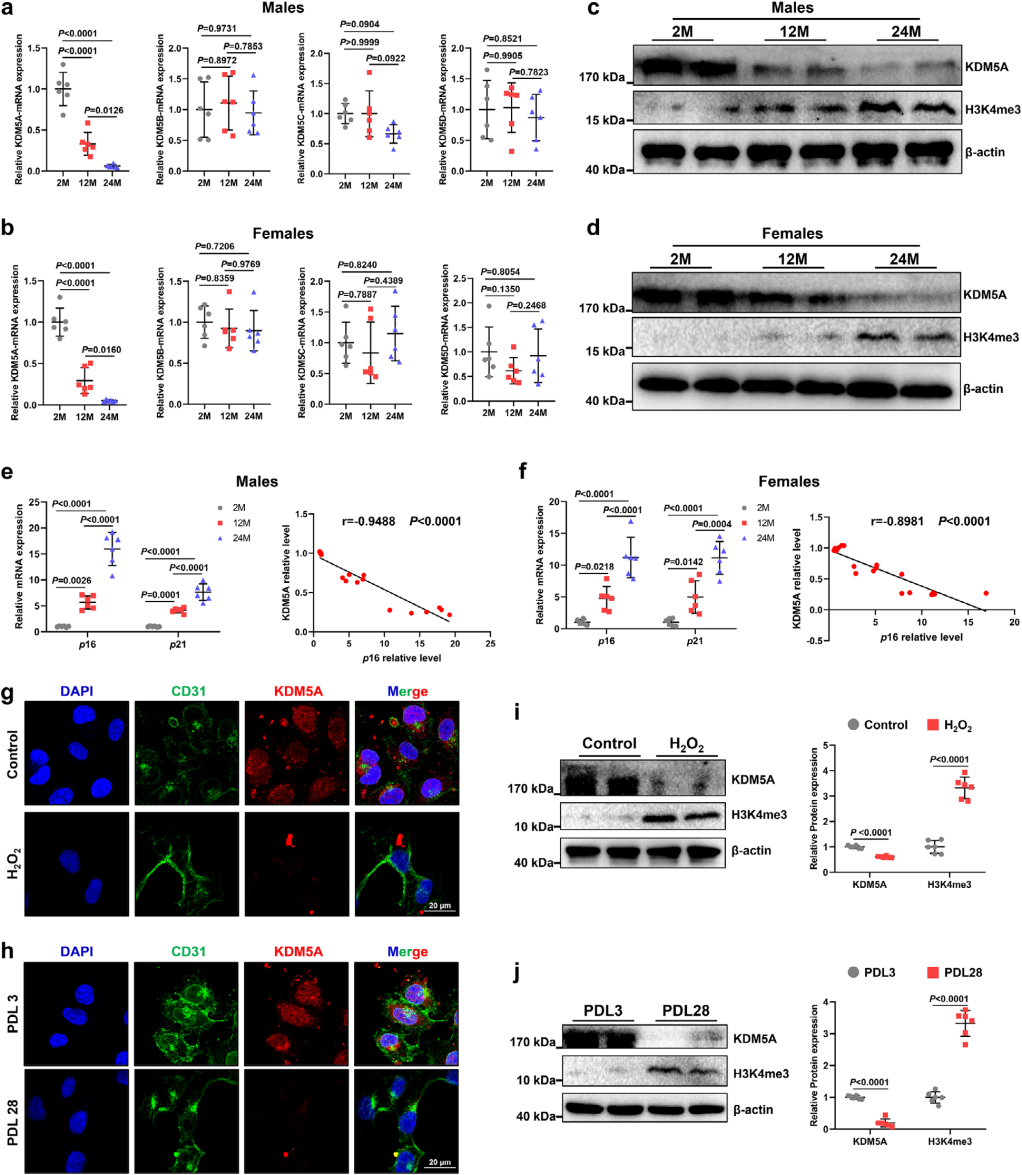
Alternations of KDM5A and H3K4me3 in the senescence process of mouse VECs. a) RT‐qPCR analysis of KDM5A, KDM5B, KDM5C and KDM5D in mouse VECs sorted from the livers of 2‐, 12‐, and 24‐month‐old male mice using flow cytometry (*n* = 6). b) RT‐qPCR analysis of KDM5A, KDM5B, KDM5C and KDM5D in VECs sorted from the livers of 2‐, 12‐, and 24‐month‐old female mice (*n* = 6). c) Western blot analysis of KDM5A and H3K4me3 in VECs sorted from the livers of 2‐, 12‐, and 24‐month‐old male mice using flow cytometry (*n* = 6). d) Western blot analysis of KDM5A and H3K4me3 in VECs sorted from the livers of 2‐, 12‐, and 24‐month‐old female mice (*n* = 6). e) Left: RT‐qPCR analysis of *p*16 and *p*21 in VECs sorted from the livers of 2‐, 12‐, and 24‐month‐old male mice (*n* = 6); Right: Simple linear regression analysis between KDM5A protein and *p*16‐mRNA expression. f) Left: RT‐qPCR analysis of *p*16 and *p*21 in VECs sorted from the livers of 2‐, 12‐, and 24‐month‐old female mice (*n* = 6); Right: Simple linear regression analysis between KDM5A protein and *p*16‐mRNA expression. g,h) Immunofluorescence analysis of KDM5A expression in H_2_O_2_‐induced and PDL‐replicative senescent VECs (Scale bar: 20 µm). i,j) Western blot analysis of KDM5A and H3K4me3 in H_2_O_2_‐induced and PDL‐replicative senescent VECs (*n* = 6). Data are presented as mean ± SD. One‐way analysis of variance analysis (ANOVA) followed by Sidak post hoc multi‐comparison test was used in (a,c). Two‐way ANOVA analysis followed by Sidak post hoc multi‐comparison test was used in (e,f,i,j).

To test whether the KDM5 family alters with age‐associated vascular senescence, we assessed the mRNA levels of *KDM5A/B/C/D* in VECs sorted from the livers of 2‐, 12‐, and 24‐month‐old mice using flow cytometry; the gating strategy used is detailed in Figure S1a (Supporting Information). Of *KDM5A/B/C/D*, only the mRNA levels of *KDM5A* significantly declined with age in VECs of both male and female mice, whereas the other genes showed no significant age‐dependent difference (**Figure** **1**a,b). Furthermore, western blot analysis confirmed that the KDM5A protein contents changed in consonance with the mRNA levels, and H3K4me3 expression increased with age accordingly (Figures 1c,d; S1b,c, Supporting Information). Subsequently, we analyzed the correlations between endothelial *KDM5A* and *p*16/*p*21, two major markers of senescent cells. The reverse transcription quantitative real‐time PCR (RT‐qPCR) results showed that *KDM5A* mRNA levels were negatively correlated with *p*16 and *p*21 in male and female mice (Figures 1e,f; S2a,b, Supporting Information). In contrast, *H3K4me3* mRNA levels in VECs of male and female mice were positively correlated with *p*16 and *p*21 (Figure S2c–f, Supporting Information).

To further clarify the relationship between VECs and KDM5A, we constructed and validated two aging VEC models in vitro: one for replicative senescence and the other for hydrogen peroxide (H_2_O_2_)‐induced senescence (Figure S3, Supporting Information). Consistent with previous findings, KDM5A expression was significantly downregulated, and H3K4me3 expression was significantly upregulated in senescent VECs compared to those in young VECs (Figure 1g–j). Taken together, these results indicate that KDM5A expression decreases in senescent VECs.

### 2.2 KDM5A Level Regulates Senescence Manifestation in VECs

**Figure 2:**
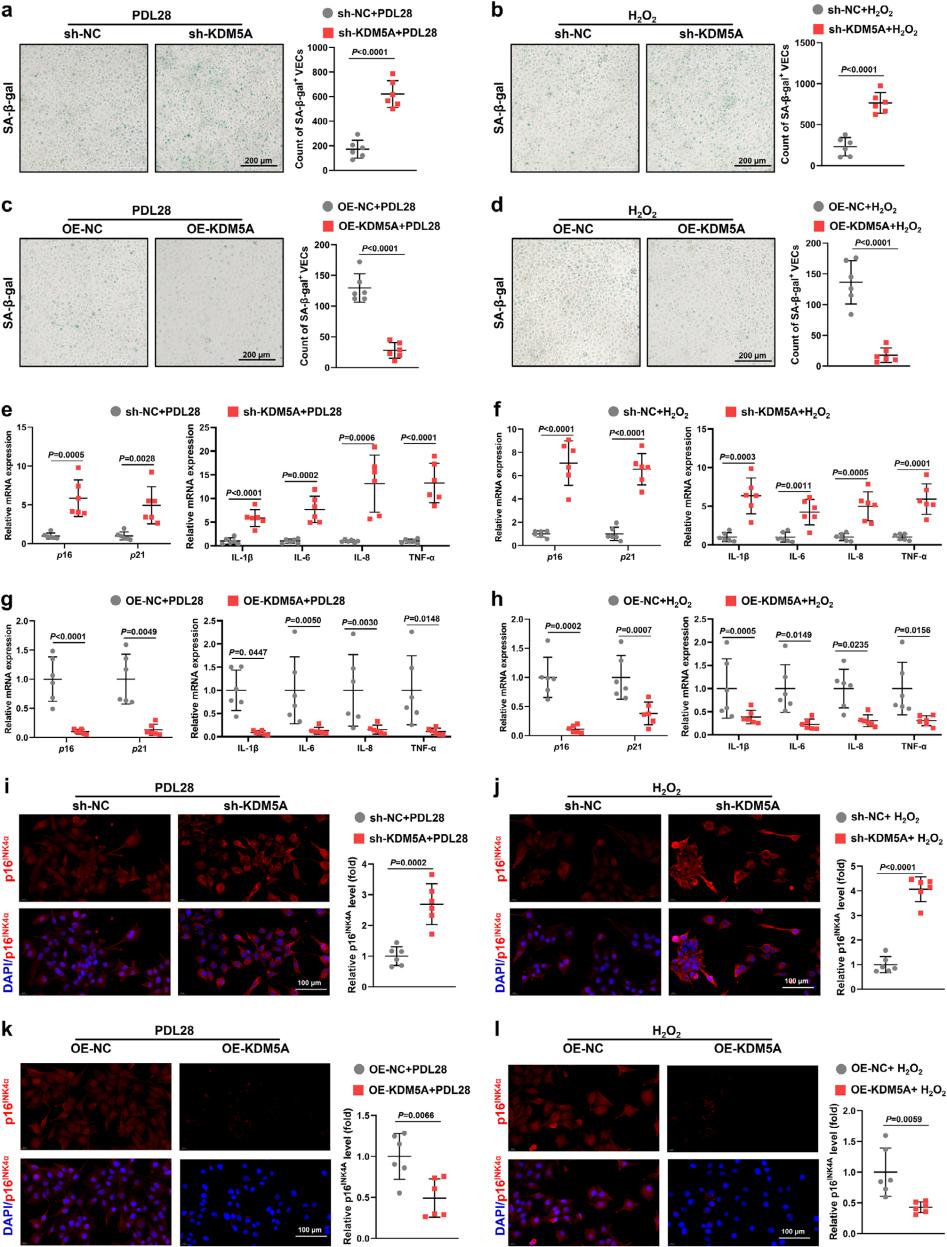
KDM5A deficiency promotes VEC senescence and KDM5A overexpression attenuates VEC senescence. a,b) SA‐β‐gal staining showing the number of senescent cells in PDL28 or H_2_O_2_‐induced VECs after transfection with or without sh‐KDM5A (Scale bar: 200 µm; *n* = 6). c,d) SA‐β‐gal staining revealing the number of senescent cells in PDL28 or H_2_O_2_‐induced VECs after transfection with or without OE‐KDM5A (Scale bar: 200 µm; *n* = 6). e,f) RT‐qPCR analysis of senescence markers (*p*16 and *p*21) and SASPs (IL‐1β, IL‐6, IL‐8, and TNF‐α) in PDL28 and H_2_O_2_‐induced VECs after sh‐KDM5A or sh‐NC transfection (*n* = 6). g,h) RT‐qPCR analysis of senescence markers (*p*16 and *p*21) and SASPs (IL‐1β, IL‐6, IL‐8, and TNF‐α) in replicative and H_2_O_2_‐induced VECs after KDM5A overexpression plasmid transfection (*n* = 6). i,j) Representative immunofluorescence images and quantitative analyses of p16^INK4α^ in PDL28 and H_2_O_2_‐induced VECs after sh‐KDM5A or sh‐NC transfection (Scale bar: 100 µm; *n* = 6). k,l) Representative fluorescent images and quantitative analyses of p16^INK4α^ in PDL28 and H_2_O_2_‐treated VECs after OE‐KDM5A or OE‐NC transfection (Scale bar: 100 µm; *n* = 6). All results are presented as mean ± SD. Unpaired 2‐tailed t test was used in (a–d,i–l). One‐way ANOVA analysis followed by Sidak post hoc multi‐comparison test was used in (e–h).

To investigate whether KDM5A affects VEC senescence, we constructed *KDM5A* knockdown (short hairpin [sh]‐*KDM5A*; Material S1, Supporting Information) and stably overexpressing (OE‐*KDM5A*) VECs (Material S2, Supporting Information). The efficiency of *KDM5A* knockdown and overexpression in VECs was validated by RT‐qPCR and western blot analysis (Figure S4, Supporting Information). Senescence‐associated beta‐galactosidase (SA‐β‐gal) and p16^INK4α^ staining showed no deterioration in the number of senescence‐positive cells and senescent phenotype in young *sh‐KDM5A* VECs or *OE‐KDM5A* VECs (Figure S5, Supporting Information). However, sh‐*KDM5A* VECs exhibited significantly more beta‐galactosidase‐positive cells in both senescence models (**Figure** **2**a,b), while the OE‐*KDM5A* group showed a significant reduction in the proportion of beta‐galactosidase‐positive cells (Figure 2c,d). Meanwhile, RT‐qPCR data demonstrated significant transcriptional elevations of *p*16*, p*21, and senescence‐associated secretory phenotypes (SASPs), such as interleukin (IL)‐1β, IL‐6, IL‐8, and tumor necrosis factor alpha (TNF‐α) in sh‐K*DM5A* VECs (Figure 2e,f), whereas OE‐*KDM5A* VECs exhibited the opposite tendency (Figure 2g,h). In addition, *KDM5A* knockdown exacerbated the generation of p16^INK4α^ in hepatic VECs (Figure 2i,j), whereas its overexpression effectively reduced p16^INK4α^ levels (Figure 2k,l). These results suggest that modulating KDM5A expression strongly influences the senescence state of VECs.

### 2.3 Endothelial KDM5A‐Deficient Mice Exhibit Shortened Lifespan and Aggravated Aging Phenotypes

**Figure 3:**
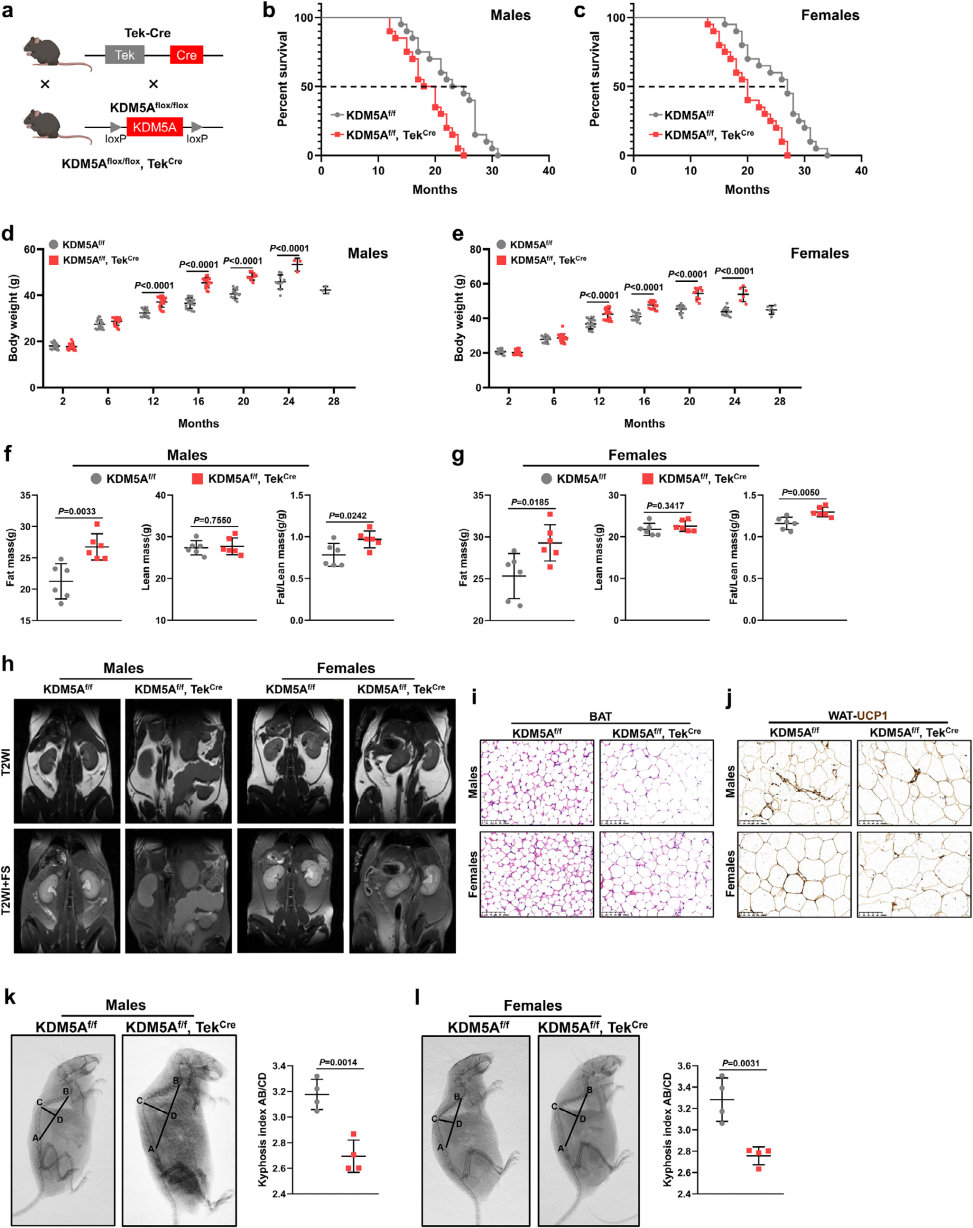
Endothelium‐specific KDM5A deficiency shortens lifespan and exacerbates adverse age‐associated signs in mice. a) Schematic of the hybridization that produces KDM5A^f/f^, Tek^Cre^ mice. b) Kaplan–Meier survival curves of male KDM5A^f/f^, Tek^Cre^, and KDM5A^f/f^ mice (*n* = 20). c) Kaplan–Meier survival curves of female KDM5A^f/f^, Tek^Cre^, and KDM5A^f/f^ mice (*n* = 20). d,e) Body weight changes of male or female KDM5A^f/f^, Tek^Cre^, and KDM5A^f/f^ mice. f) Fat masses (left), lean masses (middle), and fat/lean body mass (right) in 24‐month‐old male KDM5A^f/f^, Tek^Cre^, and KDM5A^f/f^ mice was calculated based on Echo‐MRI measurements (*n* = 6). g) Fat masses (left), lean masses (middle) and fat/lean body mass (right) in 24‐month‐old female KDM5A^f/f^, Tek^Cre^, and KDM5A^f/f^ mice (*n* = 6). h) MRI scans showing the accumulation of abdominal fat in 24‐month‐old male and female KDM5A^f/f^, Tek^Cre^, and KDM5A^f/f^ mice (fat appears white signal on T2WI and depressed signal on T2WI+FS). i) Representative H&E‐stained brown adipose tissue (BAT) sections resected from 24‐month‐old male and female KDM5A^f/f^, Tek^Cre^, and KDM5A^f/f^ mice (Scale bar: 100 µm). j) Representative UCP1‐immunostained sections of abdominal adipose tissue (WAT) sections resected from 24‐month‐old male and female KDM5A^f/f^, Tek^Cre^, and KDM5A^f/f^ mice (Scale bar: 100 µm). k,l) Representative images of whole‐body X‐rays of 24‐month‐old male or female KDM5A^f/f^, Tek^Cre^, and KDM5A^f/f^ mice (*n* = 4). Data are presented as mean ± SD. One‐way ANOVA analysis followed by Sidak post hoc multi‐comparison test was used in (d,e). Unpaired 2‐tailed t test was used in (f,g,k,l).

Taking a step further, endothelial *KDM5A*‐specific knockout mice (KDM5A^f/f^, Tek^Cre^) were generated using KDM5A^f/f^ mice (Material S3, Supporting Information) crossed with Tek^Cre^ mice (Material S4, Supporting Information) to observe alternations in age‐associated pathologies and lifespan (**Figure** **3**a). As expected, both male and female KDM5A^f/f^, Tek^Cre^ mice exhibited shorter median survival and maximum lifespan than their KDM5A^f/f^ counterparts (Figure 3b,c). In addition, these KDM5A^f/f^, Tek^Cre^ mice of both sexes exhibited significant increases in age‐related weight gain (Figure 3d,e), accompanied by higher fat mass and fat‐to‐lean body mass ratio (Figure 3f,g).

To further elucidate the persistent effect of endothelial *KDM5A* deletion on the body weight of aging mice, we measured the weights of various organs, including the liver, heart, kidney, and brain, of male KDM5A^f/f^ and KDM5A^f/f^, Tek^Cre^ mice of different ages (2, 12, and 24 months; Figure S6a, Supporting Information). Apparently, liver and abdominal fat weights were different between KDM5A^f/f^ and KDM5A^f/f^, Tek^Cre^ mice at 12 and 24 months (Figure S6b–g, Supporting Information). Magnetic resonance imaging scans revealed significant more fat accumulation in the abdominal cavity of 24‐month‐old male and female KDM5A^f/f^, Tek^Cre^ mice (Figure 3h).

Next, we investigated the difference in thermogenic capacity between 24‐month‐old KDM5A^f/f^ and KDM5A^f/f^, Tek^Cre^ mice, which reflects the potential for energy expenditure, by examining their interscapular brown adipose tissue (BAT). Compared to KDM5A^f/f^ mice, KDM5A^f/f^, Tek^Cre^ mice showed an apparently higher composition of white‐like adipocytes in BAT that had undergone whitening, indicating an anabatic loss of thermogenic ability (Figure 3i). Similarly, the expression of thermogenic uncoupling protein 1 (UCP1), which is typically scattered throughout white adipose tissue (WAT), was observed in KDM5A^f/f^ mice, but was difficult to detect in KDM5A^f/f^, Tek^Cre^ mice (Figure 3j). Conventionally, age‐related muscular and skeletal changes usually manifest as kyphosis. Here, we discovered that the kyphosis index (i.e., lower outward curvature of the spine, AB/CD) was significantly lower in 24‐month‐old KDM5A^f/f^, Tek^Cre^ mice than in KDM5A^f/f^ mice, as measured from X‐ray images (Figure 3k,l). Together, murine VEC‐specific KDM5A elimination accelerates the progression of senescence phenotypes and shortens survival time.

### 2.4 Endothelial KDM5A Deficiency Exacerbates Age‐Related Lesions in Liver

**Figure 4:**
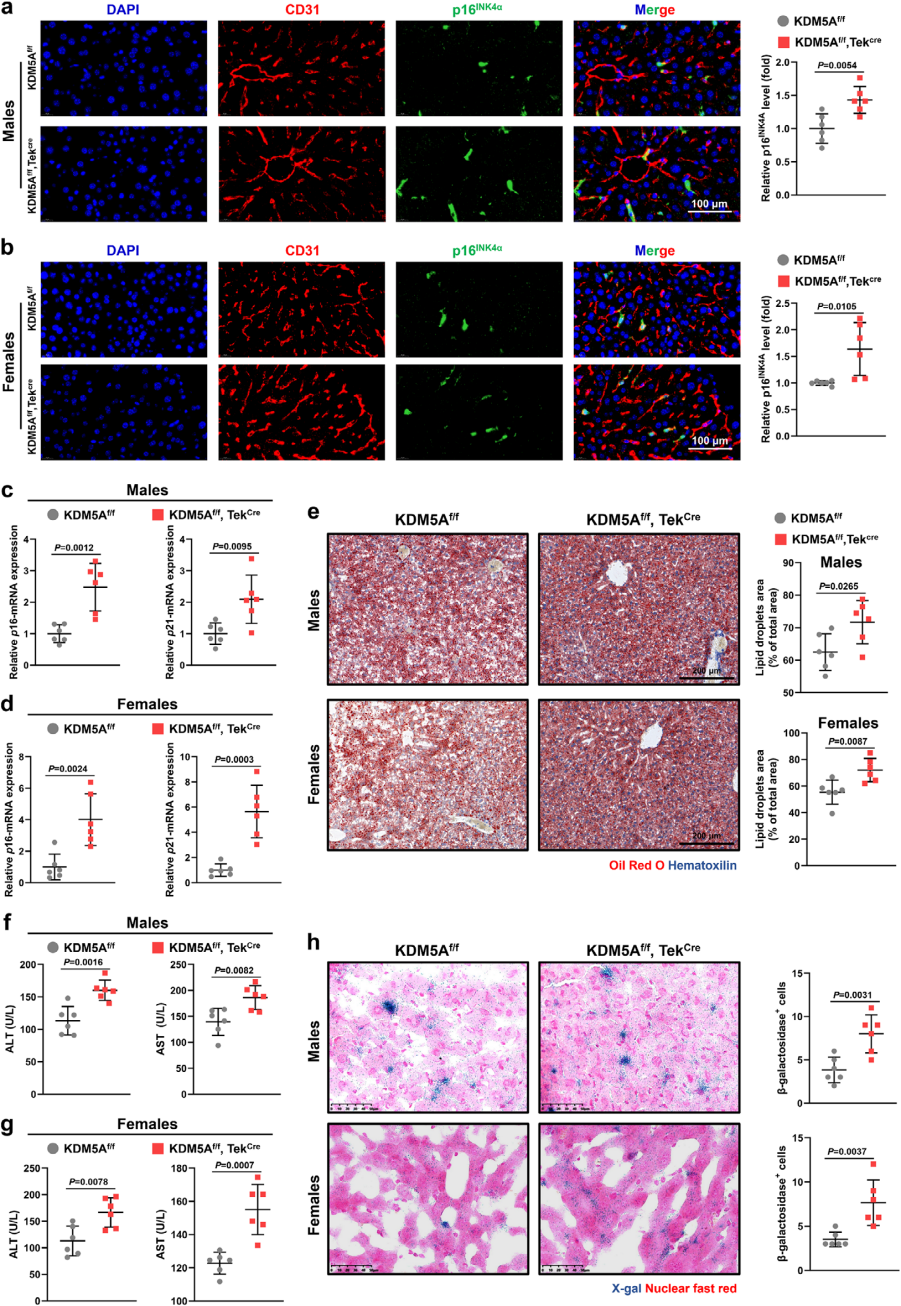
VEC KDM5A knockdown aggravates liver senescence and injury in mice. a,b) Representative immunofluorescence images and quantitative analyses of p16^INK4α^ in liver VECs from 24‐month‐old male or female KDM5A^f/f^, Tek^Cre^, and KDM5A^f/f^ mice (Scale bar: 100 µm; *n* = 6). c,d) RT‐qPCR analysis of senescence markers (*p*16 and *p*21) in liver VECs of 24‐month‐old male or female KDM5A^f/f^, Tek^Cre^, and KDM5A^f/f^ mice (*n* = 6). e) Representative liver sections and quantitative analyses in 24‐month‐old male or female KDM5A^f/f^, Tek^Cre^, and KDM5A^f/f^ mice stained with Oil Red O and counterstained with H&E (Scale bar: 200 µm; *n* = 6). f,g) Serum levels of the liver enzymes alanine transaminase (ALT) and aspartate transaminase (AST) in 24‐month‐old male or female KDM5A^f/f^, Tek^Cre^, and KDM5A^f/f^ mice (*n* = 6). h) Representative liver cryosections stained for SA‐β‐gal and quantitative analyses in 24‐month‐old male or female KDM5A^f/f^, Tek^Cre^, and KDM5A^f/f^ mice (Scale bar: 50 µm; *n* = 6). Data are presented as mean ± SD. Unpaired 2‐tailed t test was used in these results.

In previous results, we established that endothelial KDM5A deficiency leads to increased visceral fat and liver weight (Figure S6, Supporting Information). Notably, age‐related increase in liver weight is typically owing to the accumulation of fat within hepatocytes, which is also a common aging phenotype.^[^ ^39^, ^40^ ^]^ Immunofluorescence analysis revealed that p16^INK4α^ expression was marginal in the liver VECs of young *KDM5A* knockout mice, which was indistinguishable from that of non‐knockout mice (Figure S7a,b, Supporting Information); nevertheless, p16^INK4α^ expression levels were further elevated in the liver VECs of 24‐month‐old male and female KDM5A^f/f^, Tek^Cre^ mice than that of KDM5A^f/f^ mice (**Figure** **4**a,b), indicating that endothelial KDM5A deficiency triggers age‐dependent aggravation of cellular senescence. To gain insight into the impact of KDM5A deficiency on the aging of liver VECs, we sorted hepatic VECs from KDM5A^f/f^, Tek^Cre^, and KDM5A^f/f^ mice using flow cytometry. Consistent with the tissue immunofluorescence results, we observed upregulated mRNA levels of *p*16 and *p*21 in the liver VECs of 24‐month‐old KDM5A^f/f^, Tek^Cre^ mice compared with that of KDM5A^f/f^ mice (Figure 4c,d). Moreover, Oil red O staining of liver sections revealed that 24‐month‐old KDM5A^f/f^, Tek^Cre^ mice displayed higher numbers of lipid droplets and higher degrees of steatosis than KDM5A^f/f^ mice (Figure 4e). Additionally, our study disclosed elevated serum levels of alanine aminotransferase (ALT) and aspartate aminotransferase (AST) in 24‐month‐old KDM5A^f/f^, Tek^Cre^ mice compared with those in KDM5A^f/f^ mice (Figure 4f,g), suggesting that endothelial KDM5A deficiency induced relatively severe liver damage. Finally, SA‐β‐gal staining showed that endothelial KDM5A deficiency significantly increased the number of senescent cells in the liver tissues of 24‐month‐old KDM5A^f/f^, Tek^Cre^ mice (Figure 4h). However, no significant differences in hepatic lipid deposition, liver function, and aging‐related factors were noticed between 2‐month‐old KDM5A^f/f^ and KDM5A^f/f^, Tek^Cre^ mice (Figure S7c–h, Supporting Information). Overall, these data emphasize that endothelial KDM5A deficiency further exacerbates the abnormal manifestations associated with senescent livers.

### 2.5 Endothelial KDM5A Deficiency Aggravates Hepatocyte Senescence‐Associated Fatty Acid Metabolism Disorders

**Figure 5:**
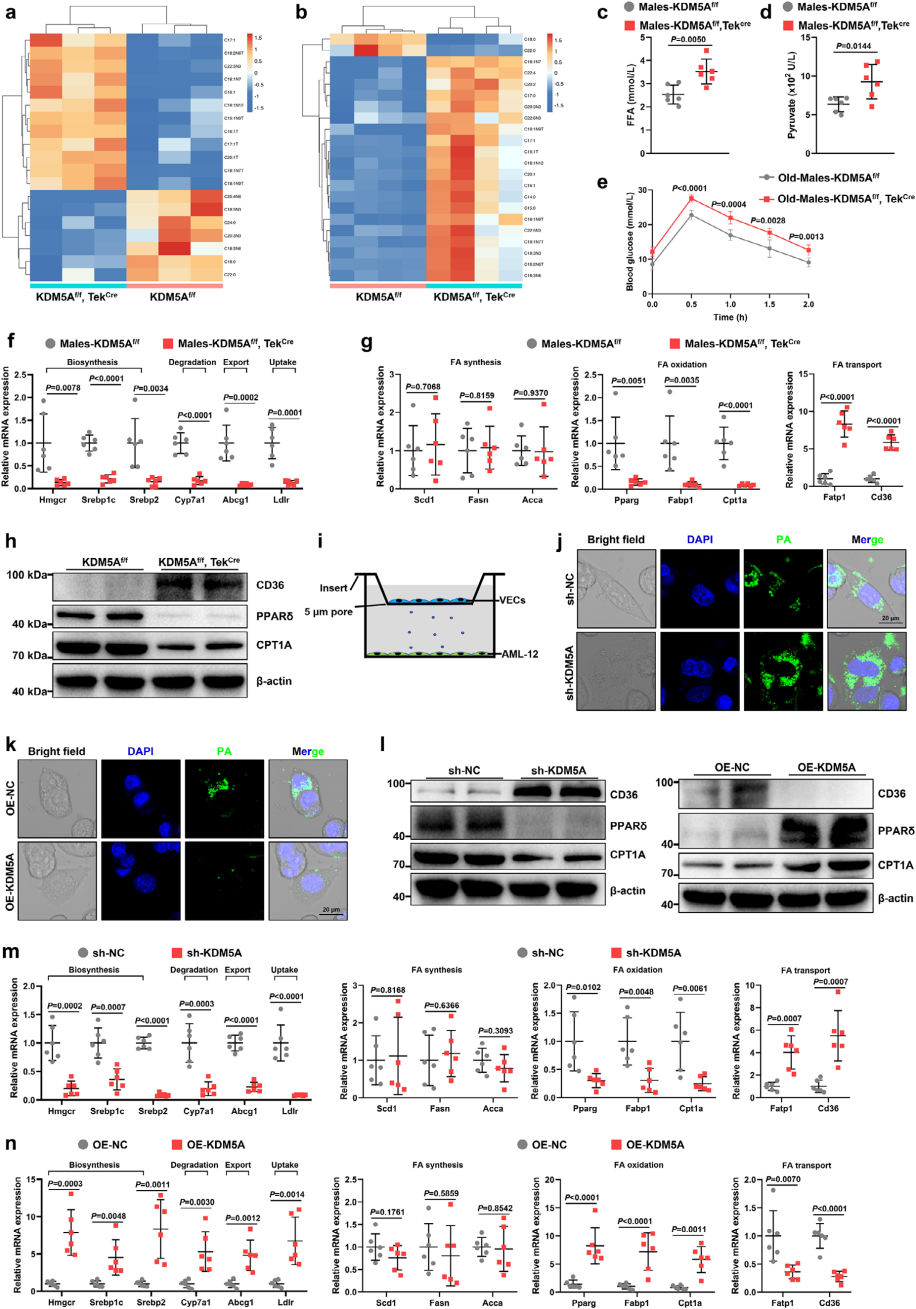
KDM5A deficiency in VECs exacerbates age‐related FA metabolism disorders. Targeted quantitative analysis of medium‐chain FA metabolism in adipose tissues (*n* = 3; a) and livers (*n* = 4; b) of 24‐month‐old male KDM5A^f/f^ and KDM5A^f/f^, Tek^Cre^ mice. c) Impact of endothelial KDM5A deficiency on plasma levels of free FA (*n* = 6). d) Fasting plasma pyruvate in 24‐month‐old KDM5A^f/f^ and KDM5A^f/f^, Tek^Cre^ mice (*n* = 6). e) Curve showing the impact of endothelial KDM5A deficiency on intraperitoneal glucose tolerance test (*n* = 6). f) RT‐qPCR analysis of cholesterol biosynthesis (Hmgcr, Srebp1c, and Srebp2), degradation (Cyp7a1), export (Abcg1), and uptake (Ldlr; n=6). g) RT‐qPCR analysis of FA synthesis (Scd1, Fasn and Acca; Left; *n* = 6), FA transport (Pparg, Fabp1, and Cpt1a; Middle; *n* = 6), and FA oxidation (Fatp1 and Cd36; Right; *n* = 6) markers. h) Western blot analysis of CD36, PPARδ, and CPT1A in the liver of KDM5A^f/f^ and KDM5A^f/f^, Tek^Cre^ mice (*n* = 4). i) Diagram of a coculture model of VECs and AML‐12. j) Representative fluorescent images of palmitic acid (PA) uptake by AML‐12 after VECs were transfected with sh‐KDM5A. k) Representative fluorescent images of PA uptake by AML‐12 after VECs were transfected with OE‐KDM5A. l) Representative western blot of CD36, CPT1A,‐ and PPARδ in the AML‐12 cells under different VEC states (*n* = 4). m) RT‐qPCR analysis of cholesterol biosynthesis (Hmgcr, Srebp1c, and Srebp2), cholesterol degradation (Cyp7a1), cholesterol export (Abcg1), cholesterol uptake (Ldlr), FA synthesis (Scd1, Fasn, and Acca), FA transport (Pparg, Fabp1, and Cpt1a), and FA oxidation (Fatp1 and Cd36) markers in AML‐12 cells after sh‐KDM5A‐transfection of VECs (*n* = 6). n) RT‐qPCR analysis of cholesterol biosynthesis, cholesterol degradation, cholesterol expor, cholesterol uptake, FA synthesis, FA transport, and FA oxidation markers in AML‐12 cells after OE‐KDM5A‐transfection of VECs (*n* = 6). Data are depicted as mean ± SD. Unpaired 2‐tailed t test was used in (c,d). One‐way ANOVA analysis followed by Sidak post hoc multi‐comparison test was used in (f,g,m,n).

As previously described, we determined that KDM5A deficiency in VECs led to abdominal obesity and hepatic fat accumulation in aging mice (Figures 3 and 4). This phenomenon and intrinsic association led us to consider that KDM5A deficiency may accelerate aging by regulating FA metabolism, thereby impairing healthy aging and reducing lifespan in mice. To understand the effects of endothelial *KDM5A* knockout on energy homeostasis, mice were individually housed in metabolic chambers for three days (acclimation), after which metabolic measurements were made for two days. The oxygen consumption rate (VO_2_), carbon dioxide production rate (VCO_2_), and energy expenditure (EE), normalized to whole body weight, were reduced in male KDM5A^f/f^, Tek^Cre^ mice compared to KDM5A^f/f^ mice (Figure S8, Supporting Information). Furthermore, targeted medium‐chain FA metabolism in the livers and adipose tissues demonstrated that aged mice with endothelial KDM5A deficiency experienced increased FA accumulation in the liver and adipose tissues (**Figure** **5**a,b). Specifically, endothelial *KDM5A* deletion had no effect on the levels of total cholesterol, triglycerides, high‐density lipoprotein cholesterol, and low‐density lipoprotein cholesterol in serum (Figure S9a, Supporting Information), whereas it resulted in an increase in the serum level of free FAs (Figure 5c). Compared to those in male KDM5A^f/f^ mice, 24‐month‐old male KDM5A^f/f^, Tek^Cre^ mice exhibited increased pyruvate (Figure 5d) and worsened glucose intolerance (Figure 5e). Furthermore, RT‐qPCR analysis of FA metabolism‐related genes revealed that endothelial *KDM5A* knockout significantly inhibited cholesterol metabolism (Figures 5f; S9b, Supporting Information), promoted FA transport, and suppressed FA oxidation (Figures 5g; S9c, Supporting Information) in male and female KDM5A^f/f^, Tek^Cre^ mice. Subsequently, protein expression analysis verified that *KDM5A* knockout led to upregulation of CD36 and downregulation of carnitine palmitoyltransferase 1A (CPT1A) and peroxisome proliferator‐activated receptor δ (PPARδ) (Figures 5h; S9d, Supporting Information). CD36, also known as FA transporter, is a multifunctional membrane protein highly expressed in adipocytes and myocytes that promotes cellular uptake of long‐chain FAs and is involved in lipid metabolism, inflammation, and immune responses.^[^ ^41^ ^]^ CPT1A is an enzyme located in the outer mitochondrial membrane that plays a key role in FA β‐oxidation.^[^ ^42^ ^]^ It is responsible for converting long‐chain FAs to acylcarnitines, which are then transported to the mitochondria for β‐oxidation, a process that is essential for catabolizing FAs to produce ATP. PPARδ is a nuclear receptor that regulates the expression of genes involved in various metabolic processes, and its activation promotes FA oxidation and utilization and reduces lipid accumulation in tissues (Figure S9e, Supporting Information).^[^ ^43^ ^]^ The above data demonstrates a highly severely disrupted pattern of FA metabolism triggered by endothelial KDM5A deficiency.

To comprehensively illustrate the role of endothelial KDM5A on FA metabolism in hepatocytes, we constructed an in vitro coculture system of VECs and AML‐12 murine hepatocytes (Figure 5i). Notably, sh‐*KDM5A* VECs mediated an elevated FA uptake by hepatocytes (Figure 5j), whereas OE‐*KDM5A* VECs significantly attenuated this process (Figure 5k). Furthermore, *KDM5A* knockdown VECs significantly promoted cholesterol metabolism, inhibited FA transport, and increased FA oxidation in hepatocytes (Figures 5l,m; S9f, Supporting Information). Conversely, *KDM5A*‐overexpressing produced the opposite effects (Figures 5l,n; S9g, Supporting Information). These results suggest that deletion of *KDM5A* in VECs may exacerbate ageing‐related FA metabolic disorders in a paracrine manner.

### 2.6 FABP4 is a Key Target of KDM5A Deficiency‐Mediated VEC Senescence and Hepatocytic FA Metabolism Disorders

**Figure 6:**
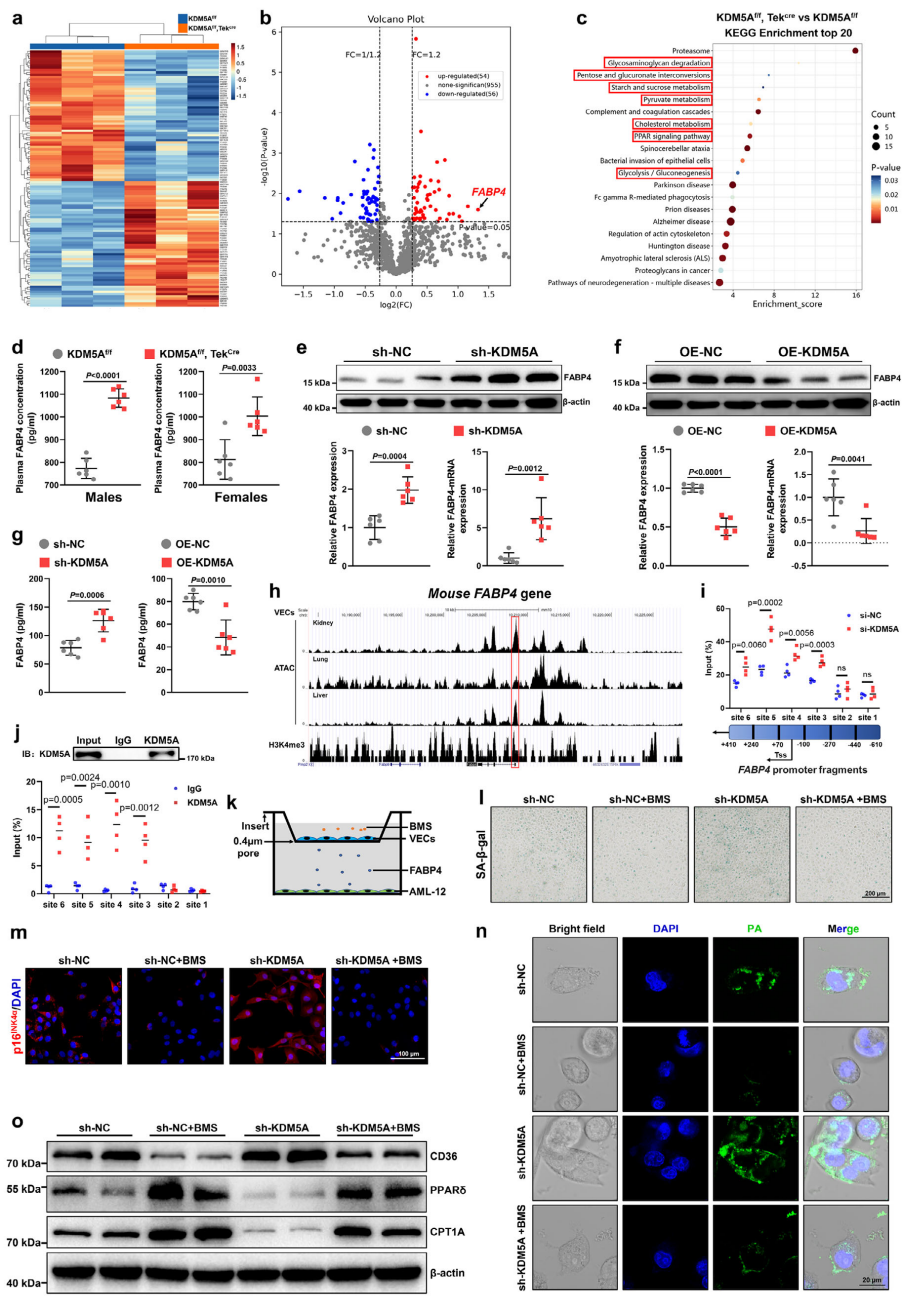
Endothelial KDM5A deficiency deteriorates hepatocyte FA metabolism disorders through FABP4 synthesis. a) Heatmap of tandem mass tags (TMT)‐labeled quantitative proteomics analysis of male KDM5A^f/f^ and KDM5A^f/f^, Tek^Cre^ mice (*n* = 3). b) Volcano plots showing differences in serum proteins detected by TMT in male KDM5A^f/f^ and KDM5A^f/f^, Tek^Cre^ mice. c) KEGG Pathway Enrichment of TMT proteomics analysis in KDM5A^f/f^, Tek^Cre^ versus KDM5A^f/f^ mice. d) ELISA for serum FABP4 levels in male or female KDM5A^f/f^ and KDM5A^f/f^, Tek^Cre^ mice (*n* = 6). e) VECs were extracted from the livers of 8‐week‐old C57BL/6 mice. Western blot and RT‐qPCR analysis of FABP4 in VECs after sh‐KDM5A transfection (*n* = 6). f) Western blot and RT‐qPCR analysis of FABP4 in VECs after OE‐KDM5A transfection (*n* = 6). g) ELISA for serum FABP4 levels in VECs after sh‐KDM5A or OE‐KDM5A transfection (*n* = 6). h) Analysis of ChIP‐seq data (GSE94829) for histone H3K4me3 methylation in VECs (Cistrome Data Browser). i) ChIP‐qPCR analysis of H3K4me3 enriched sites in *FABP4* promoter fragment. j) ChIP analysis of KDM5A binding to the *FABP4* promoter. k) Diagram of a treating strategy for VECs and AML‐12 coculture system. l) SA‐β‐gal staining showing the number of senescent cells in H_2_O_2_‐induced VECs after treatment with a small molecule FABP4 inhibitor BMS309403 (BMS) (Scale bar: 200 µm; *n* = 6). m) Representative fluorescent images and quantitative analyses of p16^INK4α^ in H_2_O_2_‐induced VECs after BMS treatment (Scale bar: 100 µm). n) Representative fluorescent images of PA uptake by AML‐12 after VECs being transfected with sh‐KDM5A and treated with BMS. o) Western blot analysis of CD36, PPARδ, and CPT1A in AML‐12 cells after sh‐KDM5A transfection and BMS treatment in upper VECs. Data are presented as mean ± SD. Unpaired 2‐tailed t test was used in (d–g). One‐way ANOVA analysis followed by Sidak post hoc multi‐comparison test was used in (i).

Given that VEC‐specific KDM5A deficiency promotes aging through paracrine signaling pathways, to identify its potential target, we extracted serum from 24‐month‐old KDM5A^f/f^ and KDM5A^f/f^, Tek^Cre^ mice for tandem mass tag‐labeled quantitative proteomic analysis. Compared to the mice in the KDM5A^f/f^ group, those in the KDM5A^f/f^, Tek^Cre^ group exhibited upregulation of 54 proteins and downregulation of 56 proteins (**Figure** **6**a). Among them, the upregulation of FABP4 was most significant (Figure 6b). Kyoto Encyclopedia of Genes and Genomes (KEGG) enrichment analysis revealed that multiple metabolic signaling pathways were involved in the deletion of endothelial *KDM5A* (Figure 6c). Next, to evaluate whether KDM5A regulates *FABP4* gene expression in VECs, we extracted VECs from male KDM5A^f/f^ and KDM5A^f/f^, Tek^Cre^ mice and treated them with H_2_O_2_ to induce senescence. RNA‐seq analysis showed that Gene Ontology and KEGG analysis were mainly enriched in aging and metabolic signaling pathways in senescent KDM5A‐deficient VECs versus KDM5A^f/f^‐derived VECs (Figure S10a,b, Supporting Information). Heatmap results showed that *FABP4* expression was significantly upregulated in *KDM5A*‐knockout VECs, and the phenotype of senescence‐associated genes (Cdkn1a and Lmnb1) was further deteriorated (Figure S10c, Supporting Information), which corresponded to the metabolic abnormalities at the animal level. As expected, serum levels of FABP4 were significantly upregulated in both female and male 24‐month‐old KDM5A^f/f^, Tek^Cre^ mice than those in the control mice (Figure 6d). Western blot and RT‐qPCR analyses showed that the transcription and synthesis of FABP4 were activated by *KDM5A* knockdown (Figure 6e), while *KDM5A* overexpression diminished FABP4 expression (Figure 6f). Enzyme‐linked immunosorbent assay also confirmed that *KDM5A* knockdown increased FABP4 levels in cell supernatants, whereas *KDM5A* overexpression decreased it (Figure 6g). The analysis (Cistrome Data Browser) of chromatin immunoprecipitation (ChIP) sequencing data (GSE94829) of histone H3K4me3 methylation in VECs demonstrated that only one H3K4me3 methylation peak existed in the *FABP4* promoter, which was compatible with the open chromatin region for transposase‐accessible chromatin using ATAT sequence (GSE111839) (Figure 6h). The ChIP‐qPCR data validated that the H3K4me3 enrichment site was enhanced in the fragments of *FABP4* promoter situated between –270 bp and +410 bp by *KDM5A* knockdown in VECs (Figure 6i). To determine whether KDM5A directly binds to *FABP4* promotor during transcription, ChIP assay was performed using either a KDM5A or IgG control antibody in VECs. The *FABP4* promoter –270 bp and +410 bp was preferentially enriched after immunoprecipitation with the KDM5A antibody compared with the IgG control antibody (Figure 6j), suggesting that KDM5A can directly bind to this region.

Additionally, we conducted serial experiments to assess whether endothelial *KDM5A* knockdown resulted in impaired FA metabolism and accelerated aging in VECs through promoting FABP4 secretion. A small molecule inhibitor of FABP4, BMS309403 (BMS), was used to treat VECs for blocking FABP4 release (Figure 6k). At the outset, SA‐β‐gal staining and p16^INK4α^ immunofluorescence showed that BMS treatment significantly attenuated H_2_O_2_‐induced VEC senescence and prevented the deteriorative senescence level caused by endothelial KDM5A deficiency (Figures 6l,m; S11a,b, Supporting Information). As a result, BMS treatment markedly reduced the uptake of FAs by hepatocytes in different environments of sh‐NC and sh‐*KDM5A* VECs (Figure 6n), particularly in the sh‐*KDM5A* VECs‐intervened groups. Western blot and RT‐qPCR analyses also confirmed that BMS efficiently inhibited FA uptake and promoted FA oxidation of AML‐12 hepatocytes, and significantly antagonized the exacerbation of FA metabolism abnormalities in lower AML‐12 cells due to KDM5A deficiency in upper VECs (Figures 6o; S11c–e, Supporting Information). These results indicate that FABP4 is a pivotal target for KDM5A‐accelerated effects on VEC senescence and impaired hepatocellular FA metabolism.

### 2.7 Endothelial FABP4 Inhibition Counteracts Endothelial KDM5A Deficiency‐Mediated Senescence

**Figure 7:**
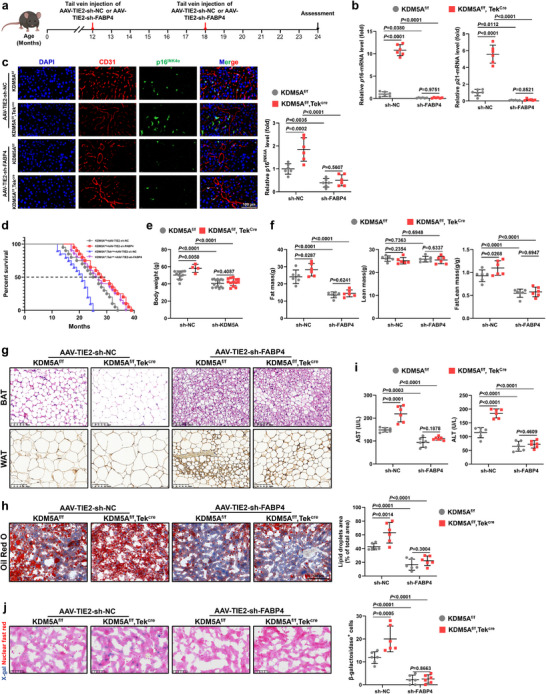
FABP4 inhibition eliminates endothelial KDM5A knockdown‐mediated shortened lifespan and aggravated senescence manifestations. a) Schematic showing the treating strategy of AAV‐TIE2‐sh‐FABP4 in aged mice. b) RT‐qPCR analysis of senescence markers (*p*16 and *p*21) in liver VECs of 24‐month‐old male KDM5A^f/f^, Tek^Cre^, and KDM5A^f/f^ mice after AAV‐TIE2‐sh‐FABP4 treatment (*n* = 6). c) Representative immunofluorescence images and quantitative analyses of p16^INK4α^ in liver VECs from 24‐month‐old male KDM5A^f/f^, Tek^Cre^, and KDM5A^f/f^ mice after AAV‐TIE2‐sh‐FABP4 treatment (Scale bar: 100 µm; *n* = 6). d) Kaplan‐Meier survival curves of male mice after diverse treatments (*n* = 20). e) Body weight changes of 24‐month‐old male mice after treatment with AAV‐TIE2‐sh‐FABP4. f) Fat masses (left), lean masses (middle), and fat/lean body mass (right) in 24‐month‐old male mice after treatment with AAV‐TIE2‐sh‐FABP4 (*n* = 6). g) Representative H&E‐stained BAT and UCP1‐immunostained sections of WAT in 24‐month‐old male mice after treatment with AAV‐TIE2‐sh‐FABP4 (Scale bar: 100 µm). h) Representative liver sections from old mice of each group stained with Oil Red O and counterstained with H&E (*n* = 6). i) Serum levels of the liver enzymes ALT and AST in 24‐month‐old male mice after different treatments. j) SA‐β‐gal staining showing the number of senescent cells after AAV‐TIE2‐sh‐FABP4 treatment (Scale bar: 50 µm; *n* = 6). Data are presented as mean ± SD. Two‐way ANOVA analysis followed by Sidak post hoc multi‐comparison test was used in these results.

FABP4 is primarily secreted by hepatocytes and VECs and considered a reliable biomarker of aging and a promising target for improving aging in older individuals.^[^ ^15^ ^]^ To further clarify whether KDM5A affects aging of mice primarily by regulating FABP4 secretion in VECs, we constructed a *FABP4* knockdown adeno‐associated virus with an endothelial‐specific promoter derived from the tyrosine kinase receptor *TIE2* **(AAV‐TIE2‐sh‐*FABP4*)**. The efficiency of *FABP4* knockdown in VECs was verified by western blot using flow‐sorted liver VECs (Figure S12a,b, Supporting Information). Moreover, no significant damage was detected in the histopathological assessment of major organs at days 14 following the administration of AAV‐TIE2‐sh‐*FABP4* (Figure S12c, Supporting Information). Therefore, our therapeutic strategy, as shown in **Figure** **7**a, involved tail vein injection of 100 µL of AAV‐TIE2‐sh‐*FABP4* (with a viral titer of 2×10^12^ genomes/mL) or an equal volume of control virus AAV‐TIE2‐sh‐*NC* into 12‐month‐old KDM5A^f/f^ and KDM5A^f/f^, Tek^Cre^ mice.

The mRNA expression analysis showed that endothelium‐targeted sh‐*FABP4* treatment intensively inhibited the expression of *p*16 and *p*21 in the liver VECs of KDM5A^f/f^ and KDM5A^f/f^, Tek^Cre^ mice and completely abolished the effects of *KDM5A* deletion (Figure 7b). Meanwhile, sh‐*FABP4* treatment decreased the fluorescent expression of p16^INK4α^ in liver VECs of 24‐month‐old KDM5A^f/f^ and KDM5A^f/f^, Tek^Cre^ mice (Figure 7c). Moreover, *FABP4* knockdown in VECs did not cause significant difference in p16^INK4α^ expression between KDM5Af/f and KDM5A^f/f^, Tek^Cre^ mice, further demonstrating that KDM5A plays a major role in regulating endothelial FABP4 during the aging process of mice (Figure 7c). Importantly, AAV‐TIE2‐sh‐*FABP4*‐treated mice exhibited longer median survival and maximum lifespan than control mice (Figure 7d). After *FABP4* knockdown in VECs, the effect of *KDM5A* deletion in VECs on mouse lifespan was almost completely antagonized (Figure 7d). In addition, body weights, fat mass, and the ratios of fat mass to lean mass were significantly reduced in sh‐*FABP4*‐treated 24‐month‐old mice compared to those in the sh‐*NC* groups, and the negative effects of *KDM5A* deletion in VECs were minimized via sh‐*FABP4* administration (Figure 7e,f). In liver‐related studies, the sh‐*FABP4* group showed distinctly reduced white adipocytes in BAT and enhanced UCP1 expression in WAT (Figure 7g), and significantly reduced deposition of lipid droplets (Figure 7h) and serum ALT and AST levels (Figure 7i). Furthermore, endothelial KDM5A deficiency‐mediated undesirable fat accumulation, infiltration, and hepatic injury did not significantly differ from those of the control group after FABP4 removal (Figure 7h,i). Additionally, SA‐β‐gal staining indicated that sh‐*FABP4* treatment decreased the number of senescent hepatocytes in the livers of 24‐month‐old KDM5A^f/f^ and KDM5A^f/f^, Tek^Cre^ mice (Figure 7j). Together, these in vivo findings reemphasize that endothelial FABP4 secretion is a major target of endothelial KDM5A‐mediated progressive senescence.

### 2.8 Maintaining KDM5A Expression Prolongs Lifespan and Optimizes Metabolism in Aging Mice

**Figure 8:**
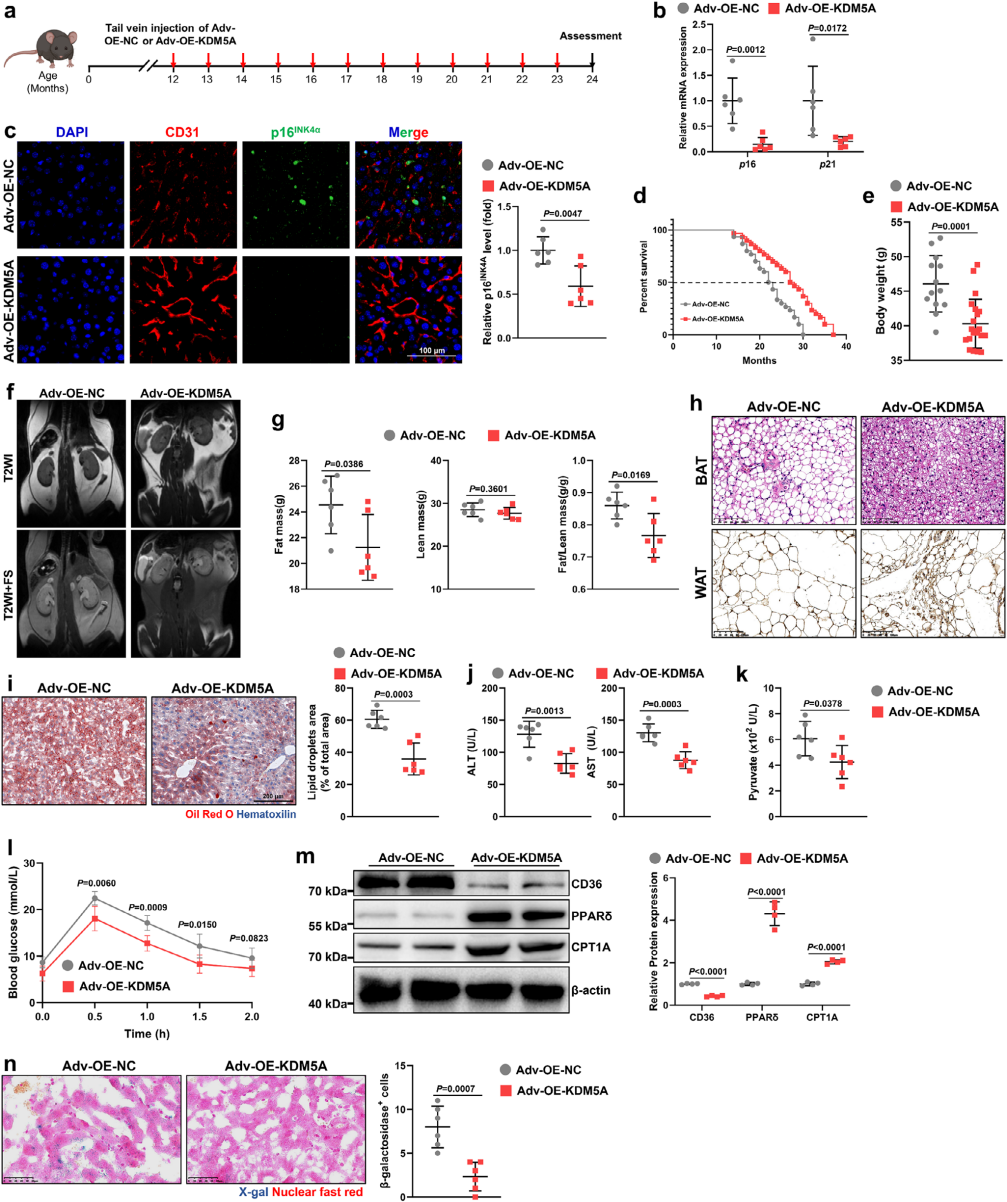
Exogenous KDM5A supplementation prolongs survival and attenuates age‐related metabolic abnormalities in mice. a) Schematic showing the treating strategy of Adv‐OE‐KDM5A in aged mice. b) RT‐qPCR analysis of senescence markers (*p*16 and *p*21) in liver VECs of 24‐month‐old male mice after Adv‐OE‐KDM5A treatment (*n* = 6). c) Representative immunofluorescence images and quantitative analyses of p16^INK4α^ in liver VECs from 24‐month‐old male mice after Adv‐OE‐KDM5A treatment (Scale bar: 100 µm; *n* = 6). d) Kaplan‐Meier survival curves of male mice after treatment with Adv‐OE‐KDM5A (*n* = 20). e) Body weight changes of 24‐month‐old male mice after treatment with Adv‐OE‐KDM5A. f) Accumulation of abdominal fat in 24‐month‐old male mice by MRI (fat shows white signal on T2WI and can be depressed on T2WI+FS). g) Fat masses (left), lean masses (middle) and fat/lean body mass (right) in 24‐month‐old male mice after Adv‐OE‐KDM5A treatment (*n* = 6). h) Representative H&E‐stained BAT and UCP1‐immunostained WAT in the liver sections of 24‐month‐old male mice after Adv‐OE‐KDM5A treatment. i) Representative liver sections from old mice of each group stained with Oil Red O and counterstained with H&E (*n* = 6). j) Serum levels of the liver enzymes ALT and AST in 24‐month‐old male after treatment with Adv‐OE‐KDM5A. k) Fasting plasma pyruvate in 24‐month‐old mice with or without Adv‐OE‐KDM5A treatment (*n* = 6). l) Curves of intraperitoneal glucose tolerance test with or without Adv‐OE‐KDM5A injection (*n* = 6). m) Western blot analysis of CD36, CPT1A and PPARδ in the liver of 24‐month‐old male mice (*n* = 4). n) SA‐β‐gal staining showing the number of senescent cells after Adv‐OE‐KDM5A treatment (Scale bar: 50 µm; *n* = 6). Data are exhibited as mean ± SD. Unpaired 2‐tailed t test as used in (e,g,i,j,k,n). One‐way ANOVA analysis followed by Sidak post hoc multi‐comparison test was used in (b,m).

To investigate therapeutic strategies for aging mice, we constructed an adenovirus‐encapsulated plasmid overexpressing *KDM5A* specifically driven by Tyrosine Kinase, Endothelial (TEK; Material S2, Supporting Information) and administered it monthly via the tail vein of 12‐month‐old male and female mice (**Figure** **8**a). In the test, we found that after 2 weeks of tail vein injection of KDM5A overexpressing adenovirus (Adv‐OE‐*KDM5A* group), the expression of KDM5A in liver VECs was significantly upregulated, while KDM5B/C did not change significantly (Figure S13, Supporting Information). Furthermore, there was no obvious damage to the aorta, liver, spleen, kidney, heart, and lungs of mice (Figure S14, Supporting Information), demonstrating that Adv‐OE‐*KDM5A*‐mediated overexpression had no significant toxic side effects or off‐target effects. After 12 months of intervention, overexpression of *KDM5A* dramatically inhibited *p*16, *p*21, and SASPs in liver VECs compared to that of the control group (Adv‐OE‐*NC* group) (Figures 8b; S15a, Supporting Information). Simultaneously, the application of Adv‐OE‐*KDM5A* significantly reduced p16^INK4α^ expression in the hepatic vasculature of 24‐month‐old mice (Figure 8c) and bestowed mice with relatively long median survival and maximum lifespan (Figure 8d). Similarly, the Adv‐OE‐*KDM5A* group showed superior age‐related body composition, as evidenced by relatively lower body weights (Figure 8e), less abdominal fat accumulation (Figure 8f), and relatively lower fat mass and ratios of fat mass to lean mass (Figure 8g). Exogenous KDM5A supplementation significantly ameliorated liver injury, as manifested by decreased white adipocytes in BAT and increased UCP1 in WAT (Figure 8h), lessened lipid droplet deposition (Figure 8i), and downregulated serum ALT and AST levels (Figure 8j). Subsequently, we evaluated the therapeutic effects of Adv‐OE‐*KDM5A* on age‐related metabolic abnormalities and found that *KDM5A* overexpression significantly reduced pyruvate (Figure 8k) and improved glucose intolerance (Figure 8l). Moreover, *KDM5A* reacquisition significantly promoted cholesterol metabolism, decreased FA transport, and facilitated FA oxidation in the liver (Figures 8m; S15b–d, Supporting Information). Ultimately, Adv‐OE‐*KDM5A* reduced the generation of senescent hepatocytes in the livers of 24‐month‐old mice (Figure 8n). Considering that KDM5A‐mediated H3K4me3 demethylation may exhibit dual roles in both senescence and tumorigenesis, we administered Adv‐OE‐*KDM5A* via tail vein injection to treat C57BL/6 nude mice and establish a tumor xenograft model. It was observed that KDM5A overexpression in endothelial cells did not significantly affect tumor initiation and growth capabilities in C57BL/6 nude mice (Figure S16, Supporting Information). Thus, KDM5A therapy represents a promising strategy for the treatment of age‐related metabolic diseases and extending lifespan.

## 3 Discussion

**Figure 9:**
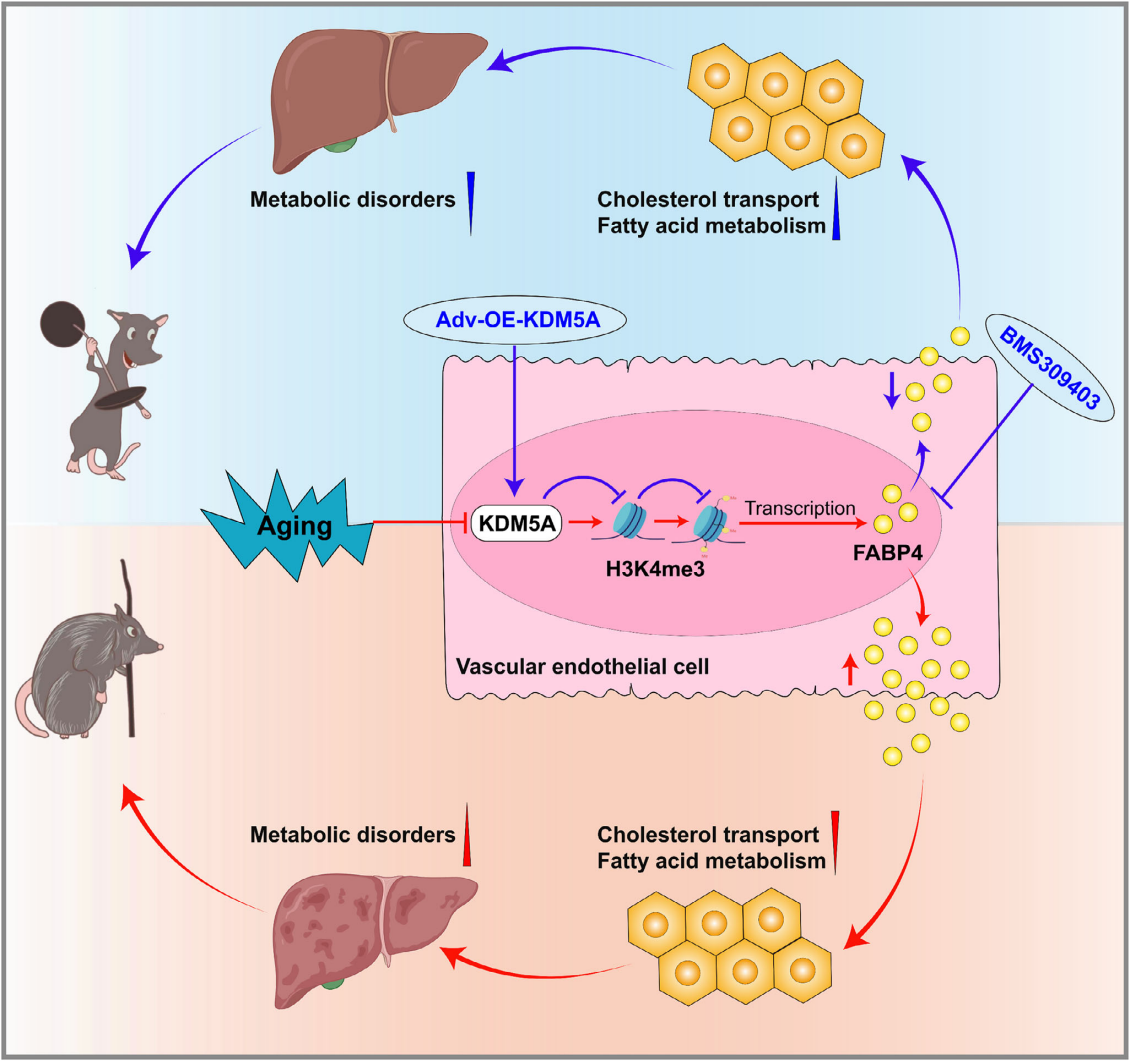
A schematic diagram showing the mechanism of action of KDM5A from VECs in regulating age‐related metabolic disorders in aged mice. The aging process in mice is accompanied by a progressive decrease in VECs‐specific KDM5A and a growth of H3K4me3 accumulation. KDM5A deficiency in VECs exacerbates age‐related FA metabolism disorders by enhancing FABP4 production, which in turn leads to shortened lifespan and worsened multiple aging phenotypes in aged mice. In contrast, KDM5A maintenance or administration of FABP4 inhibitors attenuate these senescence‐associated metabolic abnormalities.

Delaying endothelial senescence, rather than removing aging VECs, is an effective anti‐aging strategy.^[^ ^1^, ^44^ ^]^ The present study has revealed the protective role and mechanisms of VECs‐specific KDM5A in regulating age‐associated metabolic disturbance in aged mice (**Figure** **9**). We first observed reduced KDM5A and increased H3K4me3 expression in liver VECs of aging mice, and showed that endothelial KDM5A deficiency resulted in shortened lifespan and multiorgan aging phenotypes. Furthermore, our data showed that endothelial KDM5A deficiency induced aging‐associated FA metabolism disorders by enhancing H3K4me3 enrichment in the *FABP4* promoter region and subsequently upregulating its expression. We also found that maintaining KDM5A expression by adenovirus based genetic therapy could reverse multiorgan aging phenotype and prolong lifespan. Thus, we identified KDM5A, regardless of sex, as a novel therapeutic factor that delays VEC senescence, thereby extending lifespan in mice.

Notably, H3K4me3 is closely associated with endothelial aging.^[^ ^45^, ^46^, ^47^ ^]^ A recent research reported that MLL1 drives H3K4me3 on nuclear factor‐κB p65 and NOX4 promoter to increase endothelial inflammatory and oxidative phenotype.^[^ ^48^ ^]^ And KMT2E increases epigenetic H3K4me3 to increase HIF‐2α‐dependent metabolic and pathogenic endothelial activity.^[^ ^49^ ^]^ However, the role of the Jumonji C domain‐containing histone demethylase family that demethylates H3K4me3 in endothelial aging remains unclear. We noticed a decrease in KDM5A expression in aging liver VECs. In vitro and in vivo results confirmed that endothelial KDM5A deficiency accelerated VECs aging and that KDM5A overexpression substantially reversed this effect. Moreover, endothelial KDM5A deficiency induced shortened lifespan and multiorgan aging phenotype in older mice, including abdominal fat accumulation, decreased thermogenic capacity, and liver steatosis. Recombinant adenoviruses, as a category of gene therapy, have been extensively studied and used in clinical trials for cancer and vaccines.^[^ ^50^, ^51^, ^52^ ^]^ In this study, an adenovirus‐based gene therapy showed that specific supplementation of KDM5A was effective in reducing senescent VECs, ameliorating multiorgan senescence phenotype, and prolonging lifespan in aging mice. Our findings provide crucial evidence that endothelial aging by the imbalance in epigenetic modification of KDM5A and H3K4me3 may be an important driver of aging, and that measures targeting this abnormality are preclinically practicable.

Lipid metabolism disorders are a central cause of aging.^[^ ^53^, ^54^, ^55^, ^56^, ^57^ ^]^ In aged endothelial *KDM5A* knockout mice, we revealed a prominent obesity phenotype and discovered increased FA accumulation in the liver and adipose tissue, which was consistent with the changes in expression of lipid metabolism‐related enzymes. The data of a coculture system of VECs and hepatocytes showed that deletion of KDM5A in VECs exacerbated hepatic metabolic disorders of FAs, indicating the crucial paracrine manner between VECs and hepatocytes. The trimethylation of H3K4me3 in thermogenic genes is suppressed in BAT during obesity,^[^ ^58^, ^59^ ^]^ suggesting the promoting role of H3K4me3 in adipocyte differentiation and thermogenesis. These findings are inconsistent with our results on endothelial H3K4me3‐mediated age‐related abnormalities in FA metabolism. The cellular heterogeneity of H3K4me3 indicates that targeting these modifications demands greater precision and caution. Here, we propose a novel concept that age‐related FA metabolic disorders might be induced by senescent VECs, where KDM5A/H3K4me3 modification is the primary contributor.

Noteworthily, FABP4 is an adipokine secreted by adipocytes involved in FA transport,^[^ ^60^ ^]^ metabolism, and signaling.^[^ ^61^, ^62^, ^63^ ^]^ Furthermore, FABP4 ameliorates aging‐associated metabolic disorders in aged mice, specifically liver metabolism.^[^ ^15^ ^]^ The *KDM5A* knockdown group exhibited significant increase in FABP4 levels, indicating that FABP4 is an important mediator of endothelial KDM5A deficiency promoting aging. In addition, FABP4 was secreted in large amounts by VECs in aged mice, supplementing the common viewpoints that FABP4 is an adipose‐derived factor. Moreover, we also provide a new explanation that the increased age‐related secretion of endothelium‐derived FABP4 is a pathogenic factor for the lipid metabolic disorders during aging.

The scope of this study's sequencing analyses and detailed metabolic phenotyping was primarily focused on male mice to elucidate the role of endothelial KDM5A in the aging process. While this approach provided robust mechanistic insights, it inherently limited the generalizability of our findings across sexes. Investigations in female mice were limited to assessments of key phenotypes. Consequently, a comprehensive characterization of aging trajectories and metabolic consequences resulting from endothelial KDM5A deficiency in female mice remains an essential area for future research. Furthermore, the potential interplay between estrogen signaling pathways and KDM5A function represents a critical, unexplored dimension relevant to sex‐specific aging mechanisms and metabolic regulation, and investigating this specific regulatory relationship will be a major focus of our subsequent studies.

## 4 Conclusion

In summary, the present study demonstrates that endothelial KDM5A deficiency induces age‐associated FA metabolism disorders by enhancing H3K4me3 enrichment in the *FABP4* promoter, ultimately resulting in shortened lifespan and age‐related diseases. Therefore, targeting endothelial KDM5A may be a promising therapeutic approach for delaying aging and attenuating age‐related diseases.

Several potential limitations warrant further consideration and refinement. This study focused exclusively on KDM5A's role in FA metabolism (via FABP4/H3K4me3), overlooking its potential impact on glucose homeostasis. Reliance on male‐only sequencing data limits generalizability to females, ignoring sex‐specific aging mechanisms. Therapeutic targeting of endothelial KDM5A may cause off‐tissue effects (e.g., disrupting hepatic/muscular function) remains unresolved. In the future, we will use Glucose Tolerance Test/Insulin Tolerance Test and ChIP‐seq technologies to analyze KDM5A‐mediated metabolic genes, map the dual role of KDM5A in fatty acid‐glucose crosstalk, validate the findings in female populations to evaluate the effects of estrogen, and develop tissue‐specific dosing strategies to ensure treatment safety.

## 5 Experimental Section

Detailed experimental methods are available in the Supporting Information.

### Animals

All male and female mice (including routine C57BL/6 mice, KDM5A^f/f^ and KDM5A^f/f^, Tek^Cre^ mice) used in this study were purchased from Cyagen (Cyagen Biosciences Inc., Suzhou, China). The specific design and identification results of KDM5A^f/f^ and KDM5A^f/f^, Tek^Cre^ mice can be found in Materials S3 and S4 (Supporting Information). Mice were kept under 12‐h light/dark cycles at a consistent temperature and humidity. Mice were given ad libitum access to food and water. All experimental procedures were approved by the Animal Care Ethics Committees of Renji hospital, Shanghai Jiao Tong University School of Medicine (RJ2022‐0918), and Second Affiliated Hospital, Zhejiang University (2024‐008). All animal experiments were performed according to the National Institutes of Health Guide for the Care and Use of Laboratory Animals.

### Statistical Analysis

Data are expressed as mean ± standard deviation (SD). Statistical analyses were conducted using GraphPad Prism v.8.0 (GraphPad Software Inc., San Diego, CA, USA). Normality of data distribution was tested using the Kolmogorov‐Smirnov test. The Mann‐Whitney U test was used when the group data was not normally distributed or if group variances were unequal. Homogeneity of variance was analyzed using Levene's test. Continuous data with normal distribution were assessed using either student's *t* test and one‐way analysis of variance (ANOVA) with a post hoc test or two‐way ANOVA with a post hoc test (Tukey‐Kramer).

## Conflict of Interest

The authors declare no conflict of interest.

## Author Contributions

R.G., L.L., L.Y., and C.L. contributed equally to this work. R.G. and C.L. designed the experiments and collected, analyzed and interpreted data. R.G., L.L., L.Y., Y.L., B.L., and K.Y. analyzed and interpreted tandem mass tags‐labeled quantitative proteomics data. W.Y., J.S., W.W., (Jiahui Cheng) J.C., X.S., X.Z., C.C., X.X., J.Q., A.D., and (Juntao Chen) J.C. performed histopathological analyses of part of the histological techniques. A.D., (Juntao Chen) J.C., B.L., and K.Y. financially supported the project and helped with data interpretation. R.G., B.L., and K.Y. conceived and supervised the project and drafted the article. All authors contributed to the final manuscript.

## Supporting information



Supporting Information

Supporting Information

## Data Availability

The data that support the findings of this study are available from the corresponding author upon reasonable request.
