# Supplementary material for: Targeting Endothelial KDM5A to Attenuate Aging and Ameliorate Age‐Associated Metabolic Abnormalities
Source: Adv Sci (Weinh). 2025 Nov 14;13(6):e12657. doi: 10.1002/advs.202512657 (PMC12866742; doi:10.1002/advs.202512657)
Supplement: Supplementary file 1 — Supporting Information [file ADVS-13-e12657-s001.docx]

**Supporting Information**

**Targeting endothelial KDM5A to attenuate aging and ameliorate age-associated metabolic abnormalities**

Rifeng Gao *et al.*

**#Corresponding Author:** Rifeng Gao, grfvictory@zju.edu.cn; Aiqiang Dong, dr_dongaiqiang@zju.edu.cn; Juntao Chen, 2323155@zju.edu.cn; Bo Li, libo1993818@sjtu.edu.cn; Kun Yang, yk19930311@126.com.

**This PDF file includes:**

Supplementary Methods

Figs. S1 to S16

Table S1

Supplementary Materials 1-4

**Supplementary Methods**

**Tissue section staining**

Mice were anesthetized with 2% sodium pentobarbital, and tissues from mouse organs were fixed and sectioned for staining as previously described[1]. Paraffin sections (5 μm) were cut from indicated organs at specified ages, as indicated in the figure legends.

***For Oil-Red-O staining***, non-fixed tissues were embedded in OCT (Tissue-Tek) and frozen in liquid nitrogen. Slices (8-μm) were prepared using a Leica CM1950 cryostat and stored at -80°C until use. Sections were then immersed in Oil-Red-O working solution (1320-06-5, Sigma-Aldrich, USA) for 30 min and counterstained with hematoxylin. The relative area covered by red-stained oil droplets was calculated using an ImageJ software (US National Institutes of Health, Bethesda, MD, USA).

***For SA-β-gal activity cryosection staining***, freshly prepared liver cryosections (Leica CM1950 cryostat) were fixed with 0.5% glutaraldehyde in PBS for 15 min, washed with PBS supplemented with 1 mM MgCl_2_, and stained for 6–8 h in X-Gal staining solution [PBS/MgCl_2_, 0.2M K_3_Fe(CN)_6_, 0.2M K_4_Fe(CN)_6_·3H_2_O, X-Gal]. Sections were counterstained with Nuclear Fast Red (Sigma-Aldrich, USA).

***For immunostaining***, antigen retrieval was performed using citrate buffer (PH6; Zymed Laboratories) in a pressure cooker. Sections were blocked with 1% BSA and 0.5% Triton X-100. The primary antibodies used were anti-mouse CD31 (ab28364, Abcam, UK) and anti-mouse UCP1 (ab10983, Abcam). Sections were incubated overnight in primary antibodies diluted in 1% BSA and 0.5% Triton X-100 at 4 °C. Universal anti-mouse and anti-rabbit IgG were used as secondary antibodies, according to the manufacturer’s instructions (MP7500, Vector). Confocal images were captured using an Olympus FV-1000 confocal microscope (Tokyo, Japan) and analyzed using FV10-ASW 3.0 Viewer and ImageJ software.

***For histological analysis,*** the slices were stained with Hematoxylin and Eosin (H&E). The data were analyzed using the ImageJ software.

**Liver enzymes level detection in serum**

Serum alanine aminotransferase (ALT) and aspartate aminotransferase (AST) levels were determined using a COBAS C-111 chemistry analyzer (Roche, Switzerland) as previously described[1].

**Absolute quantitative analysis of long-chain FAs in targeted metabolism of mouse liver and adipose tissues**

Targeted quantitative analysis of medium-chain FA metabolism in liver and adipose tissues of 24-month-old KDM5A^f/f^ and KDM5A^f/f^, Tek^Cre^ mice was performed.

The specific methods are as follows:

***Reagents and materials***, Methanol was obtained from Thermo Fisher Scientific (Shanghai, China). N-Hexane and Trichloromethane were obtained from YONGHUA chemical Co., Ltd (Suzhou, China). Sulphuric acid and Sodium sulphate were obtained from SINOPHARM (Shanghai, China). Methyl salicylate was obtained from Sigma-Aldrich, USA. Mixed standard of 51 fatty acid methyl esters was obtained from NU-CHEK-PREP (Shanghai, China).

***Instruments***, Constant temperature water bath was obtained from Shanghai Yiheng Scientific Instrument Co., Ltd. (Shanghai, China). Refrigerated centrifuge was obtained from Hunan Xiangyi Experiment Equipment Co., Ltd. (Hunan, China). High-throughput tissue grinde was obtained from Zhejiang Meibi Instrument Co., Ltd. (Zhejiang, China). Multi tube vortex mixer was obtained from Haimen kylin-bell lab Instruments Co., Ltd (Haimen, China). Ultrasonic cleaner was obtained from Kunshan Shumei Ultrasonic Instrument Co., Ltd. (Haimen, China).

***Preparation of standard solutions***, A mixed standard stock solutions of 51 FAs (4000 μg/mL) were diluted into ten points. The calibration curve was made by adding the N-hexane covering a range from 1 to 2000 μg/mL (1, 5, 10, 25, 50, 100, 250, 500, 1000 and 2000 μg/mL). The concentration was the total concentration of each component. Stock solutions were stored at -20℃ before use and working solutions were prepared when using.

***Sample preparation***, An appropriate amount of sample was accurately weighed into a 2 mL centrifuge tube, and 1 mL chloroform methanol (2:1) solution and 100 mg glass beads were added. Then, they were put into a high-throughput tissue grinder, and shaken at 55 Hz for 1 min twice. Subsequently, the sample was centrifuged at 12000 rpm for 5 min at 4 ℃, then extracted in an ultrasonic instrument for 30 min, and all the supernatant was taken into a 10 mL glass centrifuge tube. Accurately add 2 mL 1% sulfuric acid methanol solution and fully vortex. After esterification, 1 mL n-hexane was added in a water bath at 80 ℃ for 30 min. Then 5 mL H_2_O (4 ℃) was added for washing after vortexing and standing for 2 min. Centrifuge at 3500 rpm for 10 min at 4℃ and transfer 700 μL supernatant into a 2 mL centrifuge tube; then add 100 mg anhydrous sodium sulfate powder to remove excess water; Pipette 300 μL of supernatant into a 2 mL centrifuge tube after vortexing; finally add 15 μL methyl salicylate (500 ppm) as an internal standard to the supernatant. The mixture was vortexed to and analyzed by gas chromatography-mass spectrometry analysis[2].

***Calculation formula*,** The content of sample = C*1/Amount*1000. The unit of content: μg/g. The unit of C: μg/mL. The unit of amount: mg.

Finally, it is tested on the machine under corresponding chromatographic and mass spectrometric conditions[2-3].

**TMT-labeled quantitative mouse serum proteomics**

Serum was extracted from 24-month-old KDM5A^f/f^ and KDM5A^f/f^, Tek^Cre^ mice for tandem mass tag (TMT) -labeled quantitative proteomic analysis. ***The basic process of labeling quantitative proteomics experiments is***: Extract the total protein in the sample; take out a part for protein concentration measurement and SDS-PAGE detection; take another part for trypsin digestion and labeling, and then take equal amounts of each labeled sample and mix them; Chromatographic separation. Finally, LC-MS/MS analysis and data analysis were performed on the samples. ***Bioinformatics analysis process***: Search the database for qualitative and quantitative data. After quality assessment and preprocessing, expression level analysis and functional analysis are performed respectively. Multiple common databases were used to perform functional annotation analysis on the identified proteins. GO analysis, Pathway analysis, and interaction analysis were performed on the differential proteins obtained through screening. At the same time, correlation analysis, expression pattern clustering heat map, venn analysis, etc. were performed on the differential comparison group data. In addition, based on the data situation, we investigated the relevant or interesting parts, selected key proteins and their functions or pathways, and conducted follow-up key research and validation directions.

**Metabolic cages**

Indirect calorimetry was performed using a Columbus Instruments Comprehensive Lab Animal Monitoring System (CLAMS, Columbus Instruments). Animals were placed individually in chambers for 3 consecutive days at ambient temperature (26.5 °C) with 12 h light/dark cycles. Animals had free access to food and water. Respiratory measurements were made in 20 min intervals after initial 7-9 h acclimation period. Energy expenditure was calculated from VO2 and VCO2 using the Weir formula

**Cell lines**

Mouse VECs were obtained from mouse livers by flow sorting method. The specific flow sorting strategy is shown in Extended Data Fig. 1A. VECs were cultured in endothelial cell medium (ScienCell, San Diego, CA, USA), supplemented with 5% fetal bovine serum and 1% penicillin/streptomycin (Thermo Fisher Scientific, Waltham, MA, USA).

**Isolation and Validation of Hepatic VECs**

Male C57BL/6 mice (6-8 weeks old) were anesthetized via intraperitoneal injection of 1% sodium pentobarbital (10 μL/g), followed by thoracic cavity exposure under aseptic conditions in a biosafety cabinet. Retrograde perfusion of the left ventricle with ice-cold phosphate-EDTA bufer [PEB, 0.5% (W/V) BSA, A1933, Merck; 2mM EDTA, E5391, Merck] was performed to clear erythrocytes, after which the heart was excised, atrial tissues and vasculature were dissected, and ventricular tissue was minced into 1 mm³ fragments. Enzymatic digestion [(0.1% (W/V) Collagenase,Type 2, LS004176, Worthington; 0.25% (W/V) Collagenase,Type 4, LS004188, Worthington; 0.25 U/ml Dispase II, D4693-1G, Sigma; 7.5 μg/ml Deoxyribonuclease I, LS002145, Worthington; 37°C, 40 min] was terminated with PEB containing 2% FBS, and the resulting suspension was filtered (40 μm) and centrifuged (300 × g, 5 min, 4 °C). For magnetic-activated cell sorting (MACS), CD45-negative (CD45^-^) selection was performed using anti-CD45 MicroBeads (Miltenyi Biotec) on LD Columns, followed by CD31-positive (CD31⁺) selection to isolate VECs. Purity was validated via flow cytometry (CD45^-^CD31⁺), and cells were cultured in ECM medium supplemented with ECGS and FBS under standard conditions (37°C, 5% CO₂).

**Construction of VEC senescence models**

To establish VEC senescence models, cells were induced using H_2_O_2_ or serially passaged[4]. ***Replicative senescent cell model***: During continuous cell passaging, cell numbers were counted, and the Population Doubling Level (PDL) index was calculated. The steps for calculating the PDL were as follows: At the beginning of the experiment, the initial cell count (Ni) was recorded. After each passage, the final cell count (Nf) was determined. The PDL for each passage was calculated using the formula: PDL=log(Nf/Ni)/log(2)[5]. The PDL values for each passage were summed to obtain total PDL values. The PDL index can be used to assess the aging status of cells. PDL3 and PDL28 VECs are defined as young and aging endothelial cells, respectively. ***H_2_O_2_-induced senescent cell model***: This VEC senescence model was constructed by continuous induction for 2 h using 200 μmol/L H_2_O_2_. After the cells were cultured for 24 hours, the VEC senescence rate was detected.

***RNA-seq analysis***

RNA-seq services were provided by Beijing Novogene Technology Co., Ltd (Beijing, China). Briefly, hepatic VECs were isolated from 6–8-week-old male KDM5A^f/f^ and KDM5A^f/f^, Tek^Cre^ mice and induced into a senescent model. Total RNA was extracted from senescent VECs using Trizol reagent, and RNA quality was assessed using the Agilent 2100 Bioanalyzer, ensuring an RNA integrity number (RIN) > 8.0. Libraries were prepared using the Illumina TruSeq RNA Sample Preparation Kit and sequenced on the Illumina NovaSeq 6000 platform with 150 bp paired-end reads. Quality control was performed using FastQC, and reads were aligned to the reference genome using Hisat2. Differential gene expression was analyzed with DESeq2, and gene ontology (GO) and pathway enrichment analysis were conducted using DAVID and KEGG databases to explore functional changes in senescence VECs.

**ChIP Assay**

VECs were crosslinked with 1% formaldehyde to stabilize protein-DNA interactions, followed by chromatin fragmentation into 200-500 bp fragments using sonication. The chromatin was incubated with either a KDM5A-specific antibody (NBP3-16478, Novus Biologicals) or an IgG control antibody, and the protein-DNA complexes were immunoprecipitated using Protein A/G beads. After stringent washing to remove non-specific bindings, the crosslinks were reversed by heating at 65°C, and proteins were digested with proteinase K. The purified DNA was then subjected to quantitative PCR (qPCR) to quantify the enrichment of the FABP4 promoter region. The enrichment of this region in the KDM5A antibody immunoprecipitate was compared to the IgG control, demonstrating KDM5A’s direct binding to the FABP4 promoter.

**SA-β-Gal staining**

For SA-β-gal activity cryosection staining, cells were fixed with 0.5% glutaraldehyde in PBS for 15 min, washed with PBS supplemented with 1 mM MgCl_2_, and stained for 6–8 h in X-Gal staining solution [PBS/MgCl_2_, 0.2M K_3_Fe(CN)_6_, 0.2M K_4_Fe(CN)_6_·3H_2_O, X-Gal] and counterstained with Nuclear Fast Red (Sigma-Aldrich).

**Immunofluorescence staining**

Cells were fixed in 4% paraformaldehyde for 30 min and washed three times with PBS (5 min each wash). Drop 10% BSA solution onto the cells, and then seal the cells for 1 h at 37°C. Subsequently, anti-KDM5A (ab194286, Abcam), and anti-CD31 (ab9498, Abcam) primary antibodies were diluted 1:100 with 0.1% Triton X-100 and dropped onto the cells. Cells were then cultured overnight at 4°C and then washed three times with PBS (5 min per wash). Next, the cells were treated with secondary antibodies Goat Anti-Rabbit IgG H&L (Alexa Fluor® 488 or 594; ab150077 or ab150080, Abcam) and Goat Anti-Mouse IgG H&L (Alexa Fluor® 488 or 594; ab 150113 or ab150116, Abcam), soaked in water at 37°C in the dark, and washed three times with PBS (5 min per wash). Then, the cells were stained with DAPI for 10 min at room temperature in the dark. An anti-fluorescence-quenching agent (Invitrogen, USA) was dropped onto the samples, and the cells were then covered with glass coverslips. Images were obtained using a fluorescence microscope (Leica, German).

**ELISA**

Mouse serum and cell supernatant samples were collected and stored at -80 °C. The concentrations of FABP4 in samples were determined in 96-well plates using specific ELISA kits (ab245023, Abcam), following the manufacturer’s instructions. Absorbance of each sample was measured at 450 nm.

**Construction, viral packaging and infection of Adenoviral vectors**

***KDM5A-related virus construction*,** Adenovirus overexpressing and knocking down KDM5A was generated at OBiO technology company (Shanghai, China). Briefly, the full-length KDM5A (Gene ID: NM_145997.2) was incorporated into the H225 pADV-mCMV-MCS-3xFLAG vector at the site of EcoRI and BamHI to produce pADV-mCMV-KDM5A-3xFLAG (Adv-OE-KDM5A). This shuttle vector Adv-OE-KDM5A, together with AdMax genomic plasmid, was co-transfected into HEK293 cells to produce Adv-OE-KDM5A, which was then subjected to purification and titration. For KDM5A knockdown, the KDM5A shRNA sequences used were 5'-Ccgg-ACTACCAATGGAGGATCTTAA-CTCGAG-TTAAGATCCTCCATTGGTAGT-TTTTTTg-3' (forward), and 5’-aattcaaaaaa-ACTACCAATGGAGGATCTTAA-CTCGAG-TAAGATCCTCCATTGGTAGT-3’ (reverse). Primers for these shRNAs were annealed to form double-stranded oligos, which were then inserted into the H7045 pAAV-U6-shRNA vector at the site of AgeI and EcoRI to produce AAV-KDM5A shRNA (sh-KDM5A). The adenovirus packaging, purification, and titration procedures for the shRNA construction were performed similarly as described above. Both the overexpression and knockdown control viruses were generated following the same procedures. VECs were cultured to 50–60 % confluence prior to transfection. The Adv-OE-KDM5A or sh-KDM5A was introduced into VECs, together with polybrene to generate VECs overexpressing or knocking down KDM5A. After infection, cells that have successfully integrated the viral vector were screened using medium containing ampicillin resistance.

***For FABP4*,** a FABP4-targeting sh-RNA (sh-FABP4; sequence: 5'-GGCCAAGCCCAACAUGAUCAUCAGT-3') and a negative control sh-RNA were packaged into an adeno-associated virus adeno-associated virus (AAV) (OBiO, Shanghai, China). The specific method is described above.

**AAV and Adv vector injections**

Fast the mice for 4-6 h and warm the tail to find the tail vein; use a sterile syringe and needle to prepare the AAV solution containing FABP4 (10^12^ AAV-TIE2-sh-FABP4 viral particles per mice) or the adenovirus virus solution containing KDM5A (10^11^ Adv-OE-KDM5A viral particles per mice); after fixing the mice, clean the tail with disinfectant, insert the needle after finding the vein and slowly push the Adv and AAV solutions; after completion, gently press the injection site to stop bleeding; finally, observe the mice for a few minutes to ensure that there are no bleeding or other adverse reactions, and let them recover.

**Effects of Adv-OE-KDM5A on Tumor**

To evaluate the impact of KDM5A adeno-associated virus therapy on tumors, we established a mouse tumor model. Briefly, pre-purchased C57BL/6 nude mice (Shanghai Model Organisms Center, Inc.) were injected with Adv-OE-KDM5A or Adv-OE-NC via the tail vein. After 2 weeks, allowing stable expression of KDM5A in vascular endothelial cells (VECs), A549 cells (1×10⁶) suspended in PBS were subcutaneously inoculated into the dorsal flank of the mice. One week post-inoculation, tumor diameters were measured using vernier calipers, and tumor volumes were calculated to monitor growth kinetics, with concurrent photographic documentation of tumors.

**Flow cytometry**

The liver tissues were isolated. After cutting them into pieces, the samples were digested in a buffer containing collagenase II (1.5 mg/mL), DNase I (0.5 mg/mL), and elastase (0.25 mg/mL) at 37 °C for 30 min. Subsequently, the enzymatic solution was filtered through a 70 μm cell strainer and centrifuged at 300 ×*g* for 5 min. The sample was resuspended in PBS buffer to obtain a single-cell suspension. Red blood cell lysis was performed in a Lysing Buffer at room temperature for 5 min. Next, we centrifuged the sample and resuspended it in PBS buffer. Dead cells were distinguished using a Zombie Aqua™ Fixable Viability Kit (1:1000; 423102, Biolegend Corporation). Cells were stained with fluorescently labeled antibodies against surface antigens at 4 °C for 30 min and then washed with Cell Staining Buffer. According to the manufacturer's instructions, the cells were fixed with FOXP3 Fix/Perm Buffer (1:10; 421401, Biolegend Corporation) at 4 °C for 20 min and permeabilized at 4 °C for 15 min. Afterward, the cells were washed and stained using a fluorescently labeled antibody that detects nuclear antigens at 4 °C for 30 min. Additionally, we washed the cells and resuspended them in Cell Staining Buffer for subsequent machine analysis. Finally, flow cytometry was performed using a FACS Aria III flow cytometer (BD Biosciences). The obtained data were analyzed using FlowJo 10 software (Tree Star).

***VECs sorting*:** VECs of liver tissue were labeled using antibodies against CD45 (APC-Cy7; 147717, Biolegend Corporation), CD11b (PE-Cy7; 562222, BD Biosciences), and CD31 (FITC; 160211, Biolegend Corporation). CD45^-^CD11b^-^CD31^+^ cells were VECs, and the sorting strategy can be seen in Extended Data Fig. 1A.

**Western blotting**

Tissues or cells were lysed in RIPA lysis buffer (WB0101, Biotech Well), and protein concentrations were determined using a BCA protein assay kit (P0010S, Beyotime). An equal amount of tissue protein (40–50μg) or cellular protein (20–30μg) was separated on 10–15% SDS-polyacrylamide gels by electrophoresis and subsequently transferred to a polyvinylidene difluoride membrane (ISEQ00010, Millipore, Billerica, MA, USA). The membranes were blocked with 5% non-fat milk, then incubated overnight at 4 °C with antibodies against KDM5A (1:1000; ab78322, abcam), KDM5B (1:1000; A15740, ABclonal), KDM5C (1:1000; A14104, ABclonal), H3K4me3 (1:1000; 9751, CST), CD36 (1:1000; ab252922, abcam), PPARδ (1:1000; 74076, CST), CPT1A (1:1000; ab78322, abcam), and FABP4 (1:1000; ab92501, abcam). Afterward, the membrane was washed with TBST, incubated with the corresponding horseradish peroxidase-conjugated IgG secondary antibody for 2 h at room temperature, and washed again with TBST buffer. Protein bands were visualized using an automatic chemiluminescence imaging analysis system (Bio-Rad, Hercules, CA, USA), and Pierce ECL western blotting substrate (Millipore) was employed. Signal intensity and protein band quantification were performed using Image Lab 3.0 and Image J software, respectively.

**RNA isolation and** **RT-qPCR**

The total RNA was isolated from tissues and VECs using TRIzol reagent (15596018, Ambion). Subsequently, the RNA underwent reverse-transcription into cDNA using a CFX96 real-time PCR system (Bio-Rad Laboratories, Inc., Hercules, CA, USA), and SYBR Green Real-Time PCR Master Mix Plus (Takara) was employed for subsequent reverse transcription quantitative real-time PCR. In each 10 μl reaction, 5 μl of SYBR Green dye, 1 μl of cDNA, 0.5 μl of forward primer, 0.5 μl of reverse primer, and 3 μl of ddH_2_O were included. The cycling parameters comprised 39 cycles of 95 ℃ for 30 s, 95 ℃ for 5s, and 60 °C for 30s. The data were analyzed using the 2-^△△^Ct method, with mRNA levels normalized to β-actin gene expression. The primer sequences are shown in the *Supplementary Table*.

**Imaging assessments**

***Magnetic resonance imaging (MRI) scans*:** Age-related alterations in fat distribution were detected using a 7-Tesla dedicated animal scanner (BioSpec 70/20 USR, Bruker, Ettlingen, Germany) equipped with a one-channel circularly polarized volume coil (Bruker BioSpec MRI GmbH). The mice were anesthetized throughout the examination using a mixture of oxygen and air, delivered with 2.0–2.5% isoflurane. Respiratory monitoring (SA Instruments Inc., Stony Kyok, NY, USA) was performed to maintain a respiratory rate of 30–50 breaths per min. Mice were positioned prone during scanning, and their rectal temperature was monitored and maintained at 37 ± 0.5 ℃. The scanning scheme consisted of a T2-weighted (T2WI) turbo Rapid Acquisition with Relaxation Enhancement (RARE) sequence and a T2WI turbo RARE fat-suppression (FS) sequence. T2WI and T2WI+FS images were generated using the following parameters: TE, 24 ms; TR, 2000 ms; FOV, 70 × 40 mm^2^; slice interval = 0.8 mm, slices 20; matrix, 280 × 160; average, 6. All sequences were triggered by respiratory gating to prevent artifacts. Subsequent MRI data analysis was performed using the post-processing software of the Biospin system (Paravision 6.0.1; Bruker, Ettlingen, Germany).

***X-ray analysis:*** Results of the lateral bone morphology analysis in mice from different groups were obtained using a digital X-ray system (OPTIMA XR646HD, GE, Germany) according to the manufacturer’s instructions.

**Supplementary Figures**

**
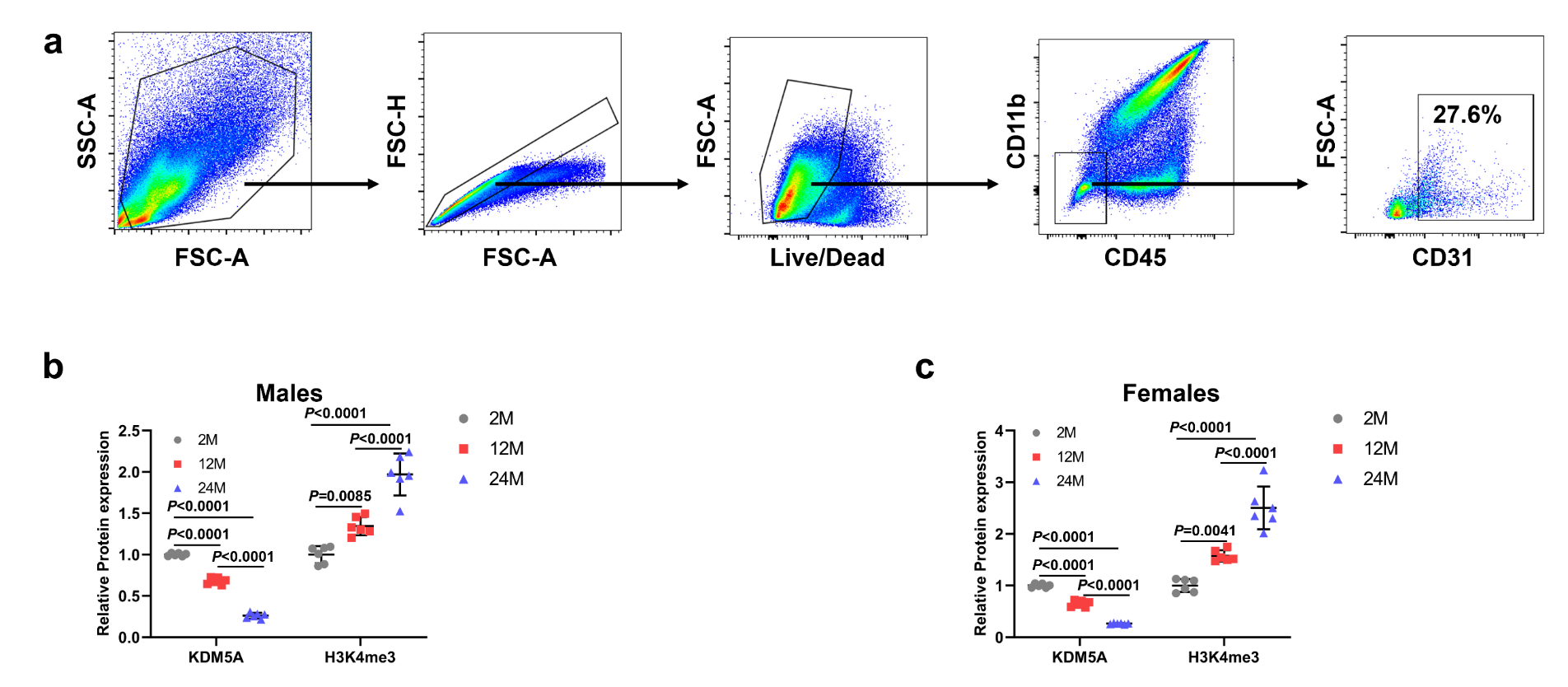
**

**Figure S1. The changes of KDM5A and H3K4me3 in VECs sorted from the livers of 2-, 12-, and 24-month-old male mice.** a) Flow sorting strategy for liver VECs from male and female mice at 2, 12 and 24 months of age; liver VECs are labeled with CD45^-^CD11b^-^CD31^+^. b) Western blot quantitative analysis of KDM5A and H3K4me3 in VECs sorted from the livers of 2-, 12-, and 24-month-old male mice using flow cytometry (*n* = 6). c) Protein quantitative analysis of KDM5A and H3K4me3 in VECs sorted from the livers of 2-, 12-, and 24-month-old female mice (*n* = 6). Data are presented as mean ± SD. Two-way ANOVA analysis followed by Sidak post hoc multi-comparison test was used in (b and c).


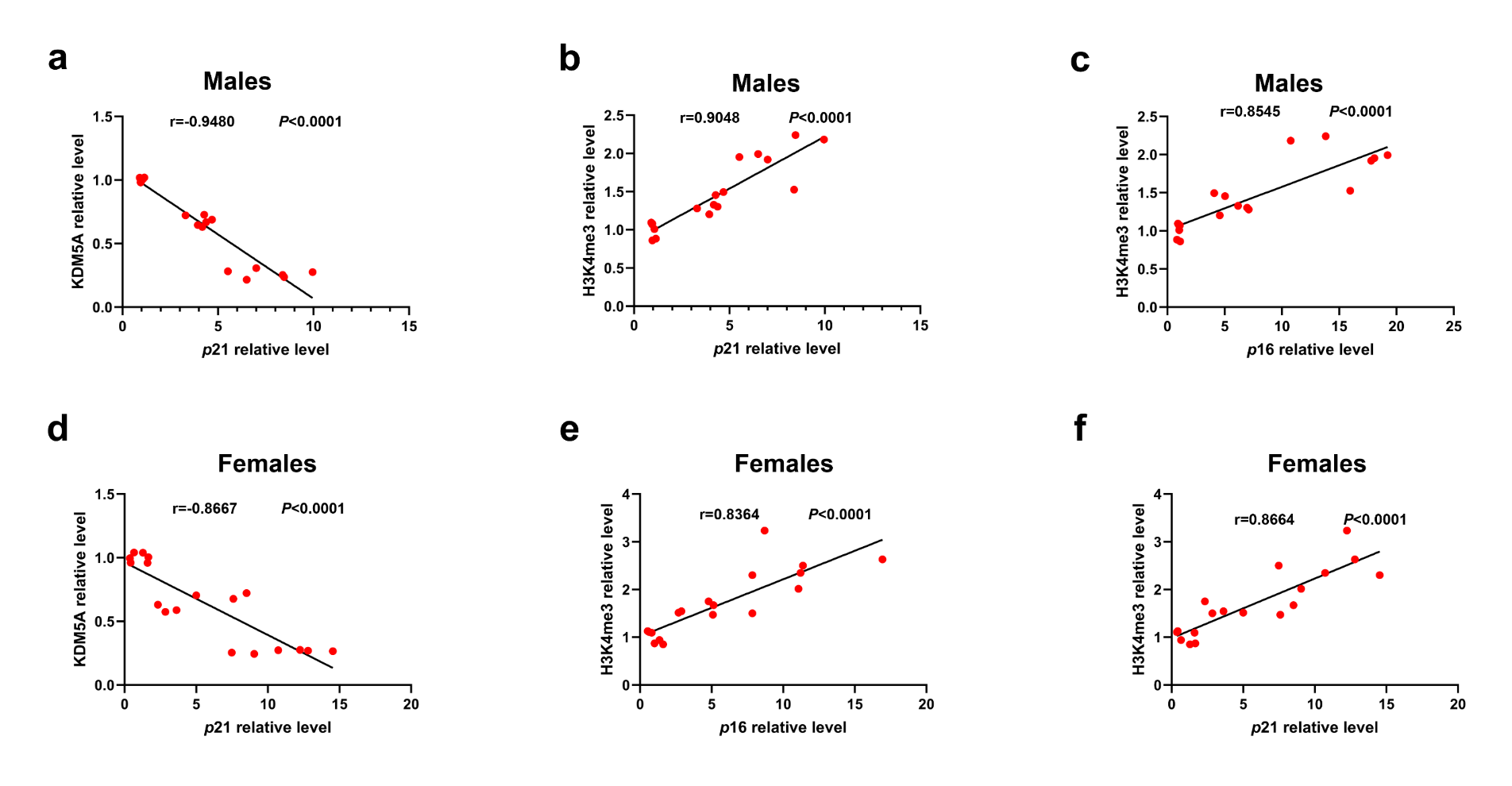


**Figure S2. Correlation analysis between KDM5A/H3K4me3 protein and senescence genes.** a) Simple linear regression analysis between KDM5A protein and *p*21 mRNA expression in the VECs of male mice. b) and c) Simple linear regression analysis between H3K4me3 protein and *p*21 or *p*16 mRNA expression in male VECs. d) Simple linear regression analysis between KDM5A protein and *p*21 mRNA expression in female VECs. e) and f) Simple linear regression analysis between H3K4me3 protein and *p*16 or *p*21 mRNA expression in female VECs.

**
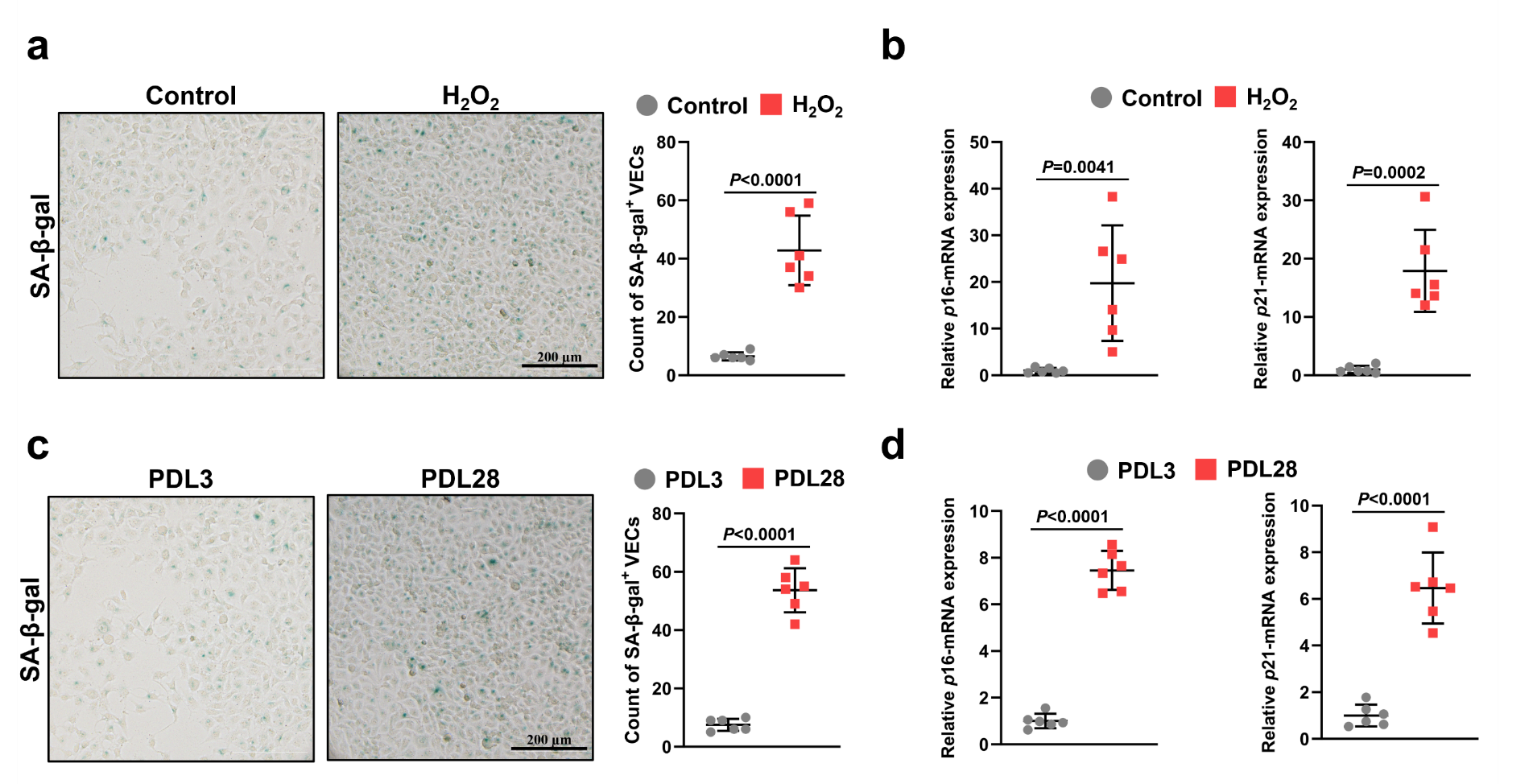
Figure S3. Validation of the aging VEC models.** a) SA-β-gal staining showing the number of senescent cells in H_2_O_2_-induced VECs (Scale bar: 200 μm; *n* = 6). b) RT-qPCR analysis of senescence markers (p16 and p21) in H_2_O_2_-induced VECs (*n* = 6). c) SA-β-gal staining showing the number of senescent cells in PDL28 VECs (Scale bar: 200 μm; *n* = 6). d) RT-qPCR analysis of senescence markers (p16 and p21) in PDL28 VECs (*n* = 6). Data are displayed as mean ± SD. Unpaired 2-tailed t test was used in Figure S4.

**
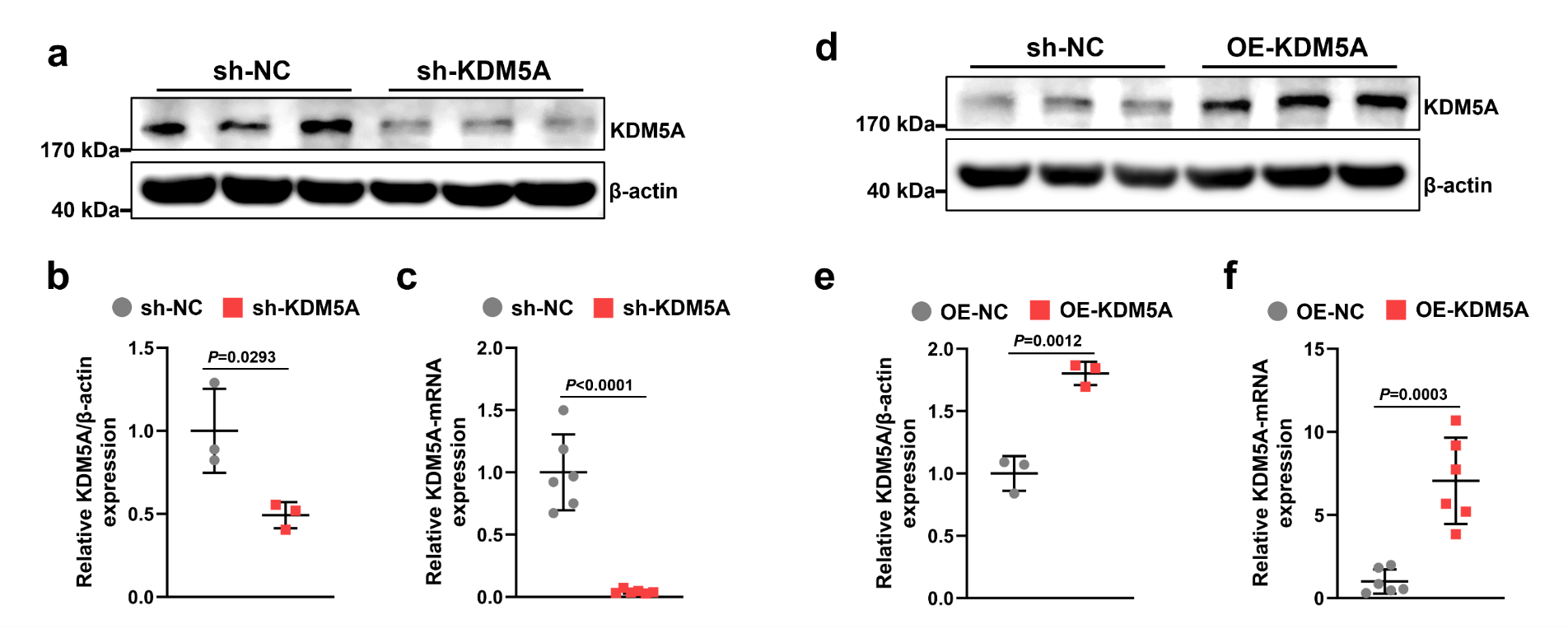
**

**Figure S4. Transfection efficiency of KDM5A in VECs.** a) and b) Western blot analysis of KDM5A levels in VECs after sh-KDM5A transfection (*n* = 3). c) RT-qPCR analysis of KDM5A levels in VECs after sh-KDM5A transfection (*n* = 6). d) and e) Western blot analysis of KDM5A levels in VECs after OE-KDM5A transfection (*n* = 3). f) RT-qPCR analysis of KDM5A levels in VECs after OE-KDM5A transfection (*n* = 6). Data are presented as mean ± SD. Unpaired 2-tailed t test was used.

**
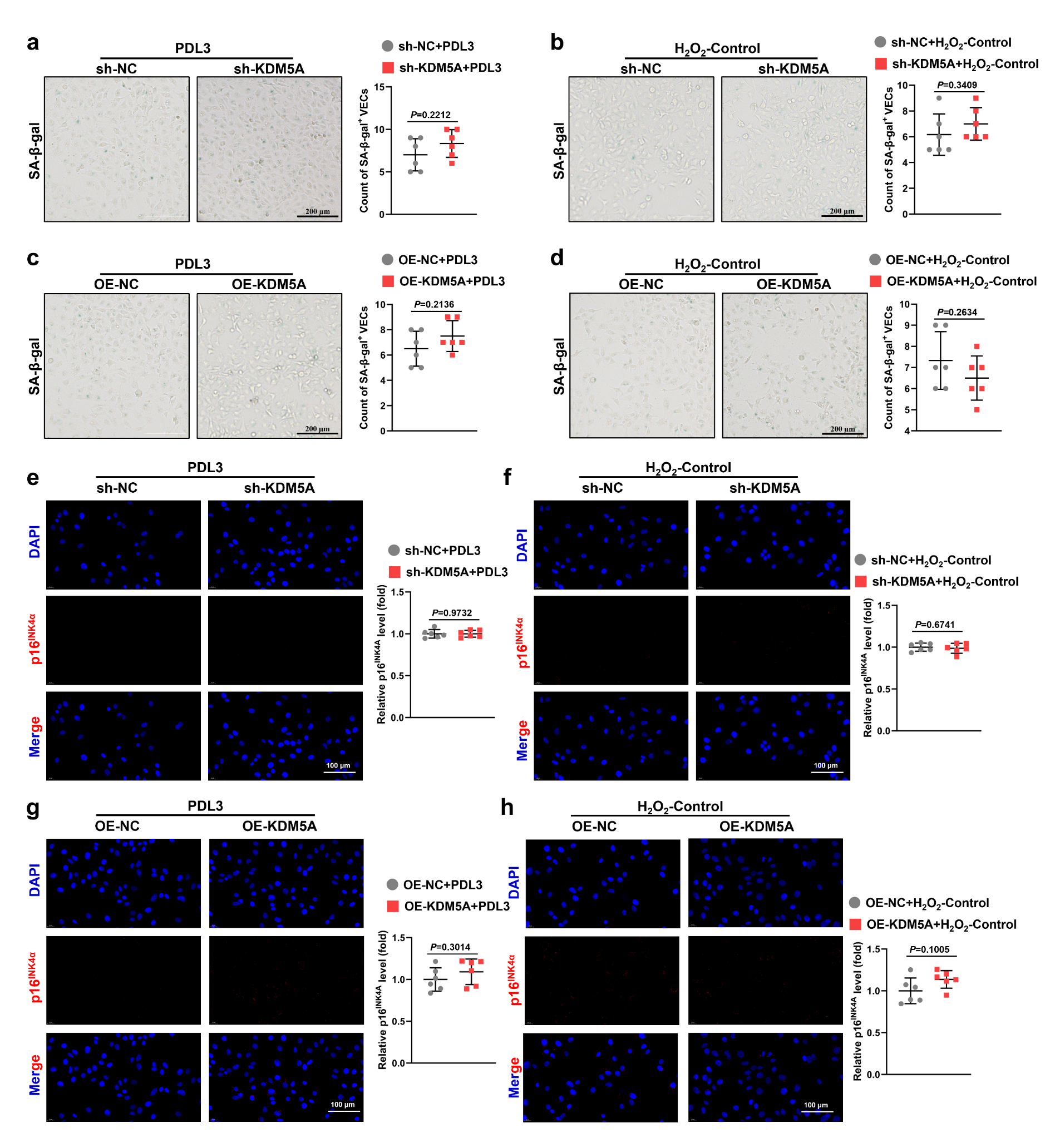
**

**Figure S5. Effects of KDM5A deficiency and overexpression on the senescent phenotype of young VECs.** a-b) SA-β-gal staining showing the number of senescent cells in PDL3 or H_2_O_2_-Control VECs after transfection with or without sh-KDM5A (Scale bar: 200 μm; *n* = 6). c-d) SA-β-gal staining revealing the number of senescent cells in PDL3 or H_2_O_2_-Control VECs after transfection with or without OE-KDM5A (Scale bar: 200 μm; *n* = 6). e-f) Representative immunofluorescence images and quantitative analyses of p16^INK4α^ in PDL3 or H_2_O_2_-Control VECs after sh-KDM5A or sh-NC transfection (Scale bar: 100 μm; *n* = 6). g-h) Representative fluorescent images and quantitative analyses of p16^INK4α^ in PDL3 or H_2_O_2_-Control VECs after OE-KDM5A or OE-NC transfection (Scale bar: 100 μm; *n* = 6). Data are displayed as mean ± SD. Unpaired 2-tailed t test was used in Figure S5.


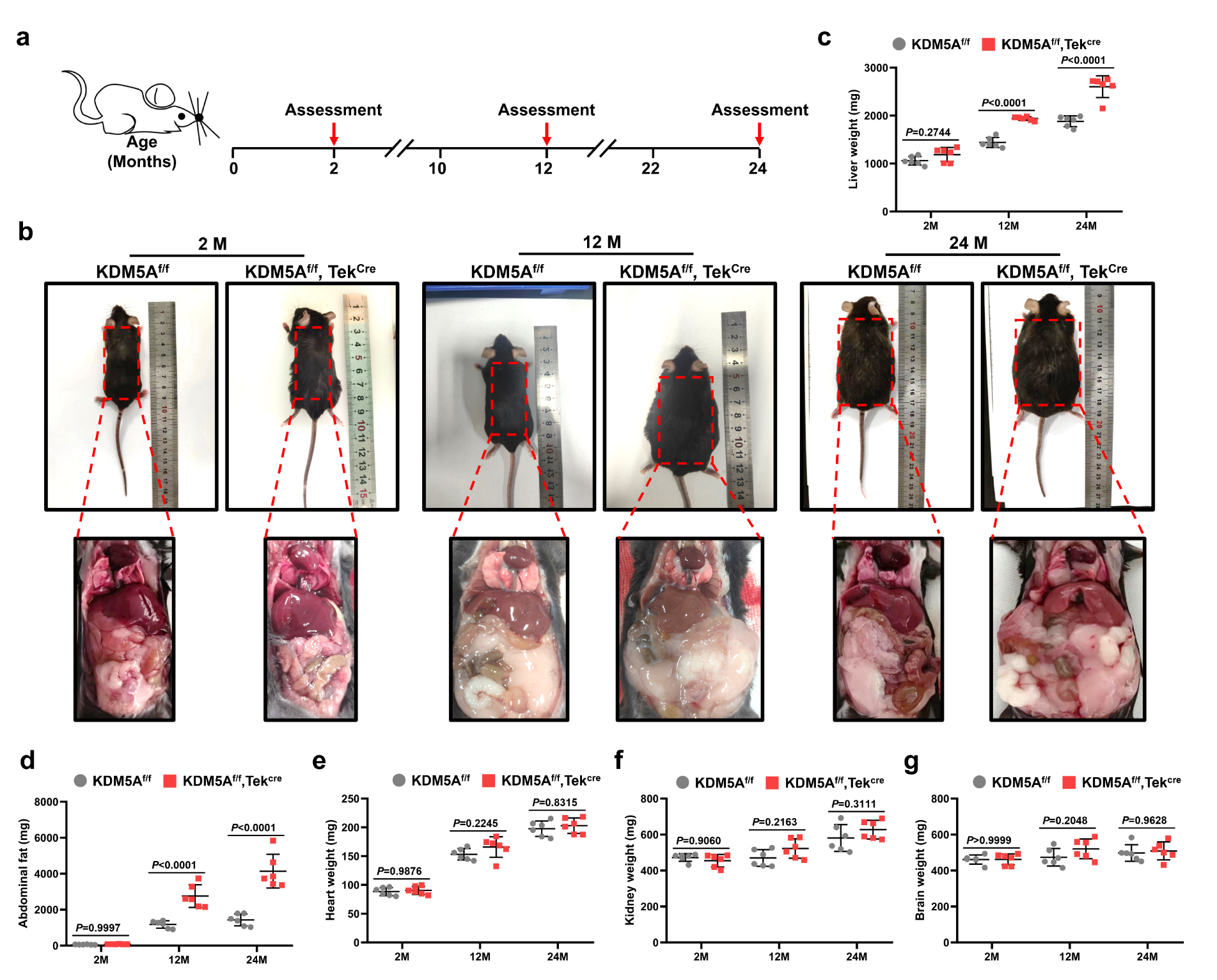


**Figure S6. Abdominal fat and multiple organ weights of KDM5A^f/f^, Tek^Cre^ and KDM5A^f/f^ mice at different ages.** a) Schematic showing the assessment strategy in male KDM5A^f/f^, Tek^Cre^ and KDM5A^f/f^ mice. b) Representative overall morphology as well as thoracic and abdominal organ morphology of 2-, 12-, and 24-month-old KDM5A^f/f^, Tek^Cre^ and KDM5A^f/f^ mice. c) Liver, d) abdominal fat, e) heart, f) kidney, and g) brain weights of KDM5A^f/f^, Tek^Cre^ and KDM5A^f/f^ mice at 2, 12, and 24 months of age. Data are displayed as mean ± SD. One-way ANOVA analysis followed by Sidak post hoc multi-comparison test was used in (c, d, e, f and g).

**
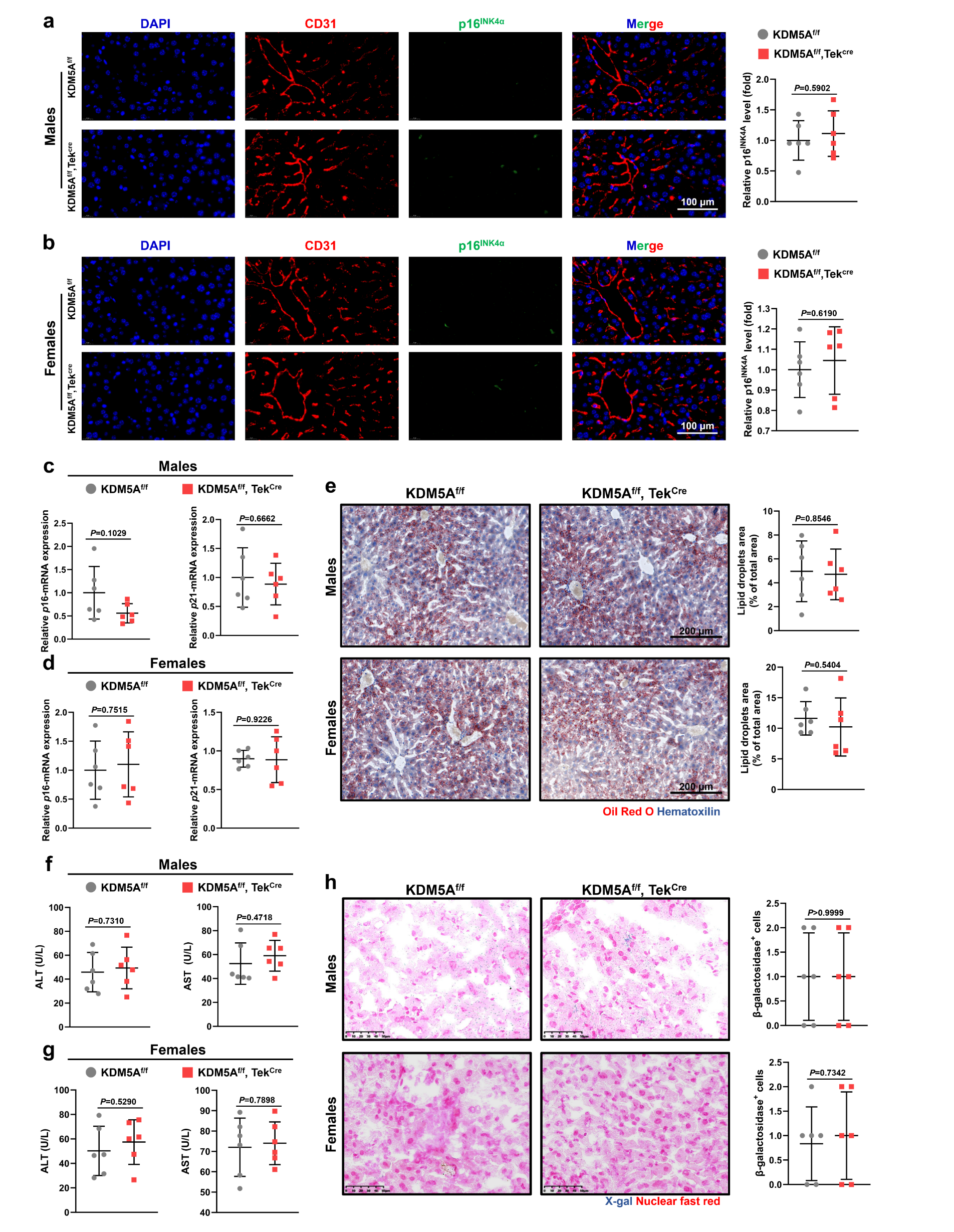
Figure S7. Effects of endothelial KDM5A deficiency on young mouse livers.** a) and b) Representative immunofluorescence images and quantitative analyses of p16^INK4α^ in liver VECs from 2-month-old male or female KDM5A^f/f^, Tek^Cre^ and KDM5A^f/f^ mice (Scale bar: 100 μm; *n* = 6). c) and d) RT-qPCR analysis of senescence markers (*p*16 and *p*21) in liver VECs of 2-month-old male or female KDM5A^f/f^, Tek^Cre^ and KDM5A^f/f^ mice (*n* = 6). e) Representative liver sections and quantitative analyses in 2-month-old male or female KDM5A^f/f^, Tek^Cre^ and KDM5A^f/f^ mice stained with Oil Red O and counterstained with H&E (Scale bar: 200 μm; *n* = 6). f) and g) Serum levels of the liver enzymes alanine transaminase (ALT) and aspartate transaminase (AST) in 2-month-old male or female KDM5A^f/f^, Tek^Cre^ and KDM5A^f/f^ mice (*n* = 6). h) Representative liver cryosections of SA-β-gal staining and quantitative analyses in 2-month-old male or female KDM5A^f/f^, Tek^Cre^ and KDM5A^f/f^ mice (Scale bar: 50 μm; *n* = 6). Data are displayed as mean ± SD. Unpaired 2-tailed t test was used.

**
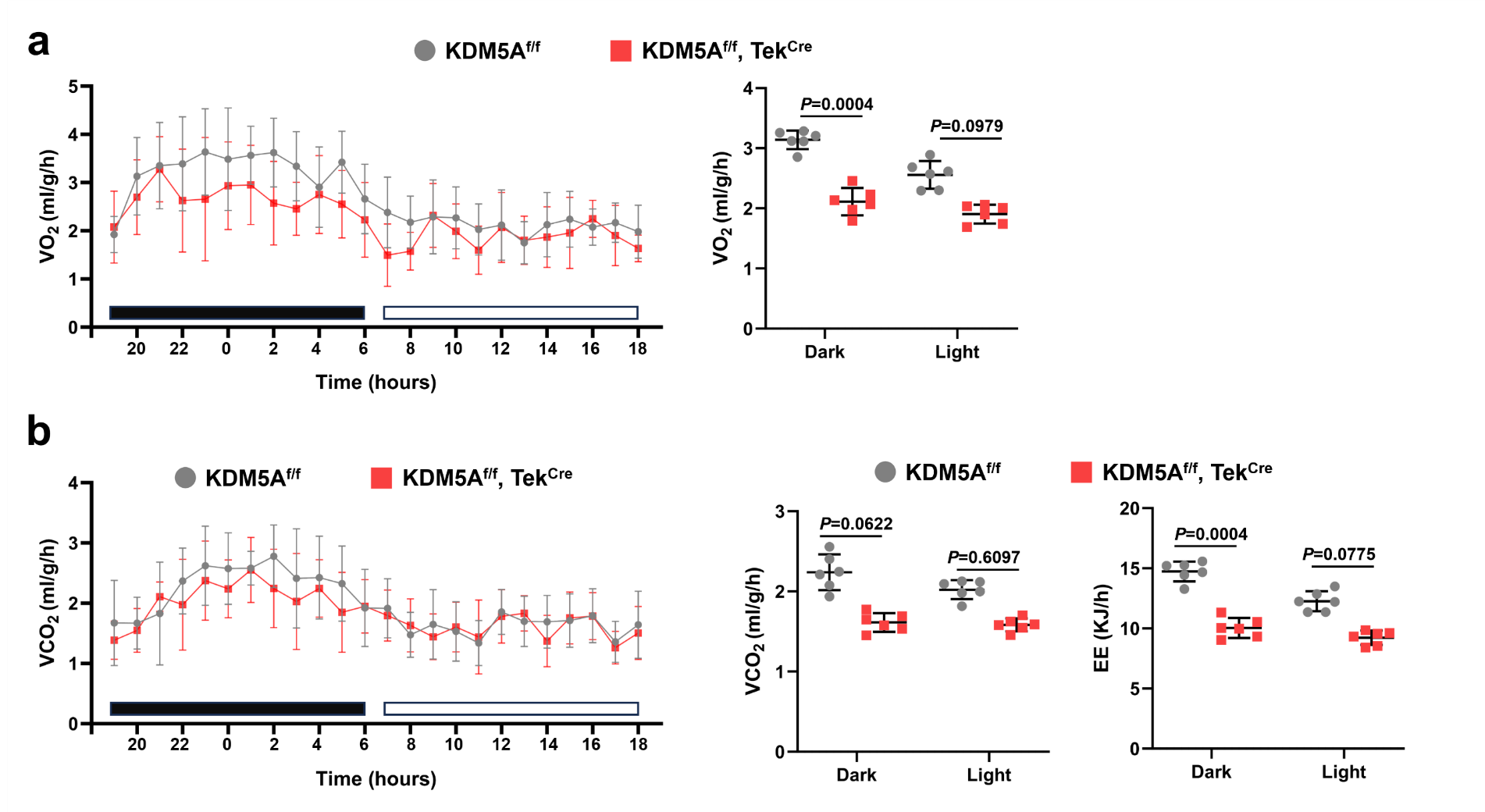
Figure S8. VEC-KDM5A knockdown reduced energy expenditure.** a-b) 24-month-old male KDM5A^f/f^, Tek^Cre^ and KDM5A^f/f^ mice were placed in metabolic cages to measure oxygen consumption rate (VO_2_; *n* = 6), carbon dioxide productivity (VCO_2_; *n* = 6), and energy expenditure (EE; *n* = 6). All results are presented as mean ± SD. Unpaired 2-tailed t test was used in (a and b).

**
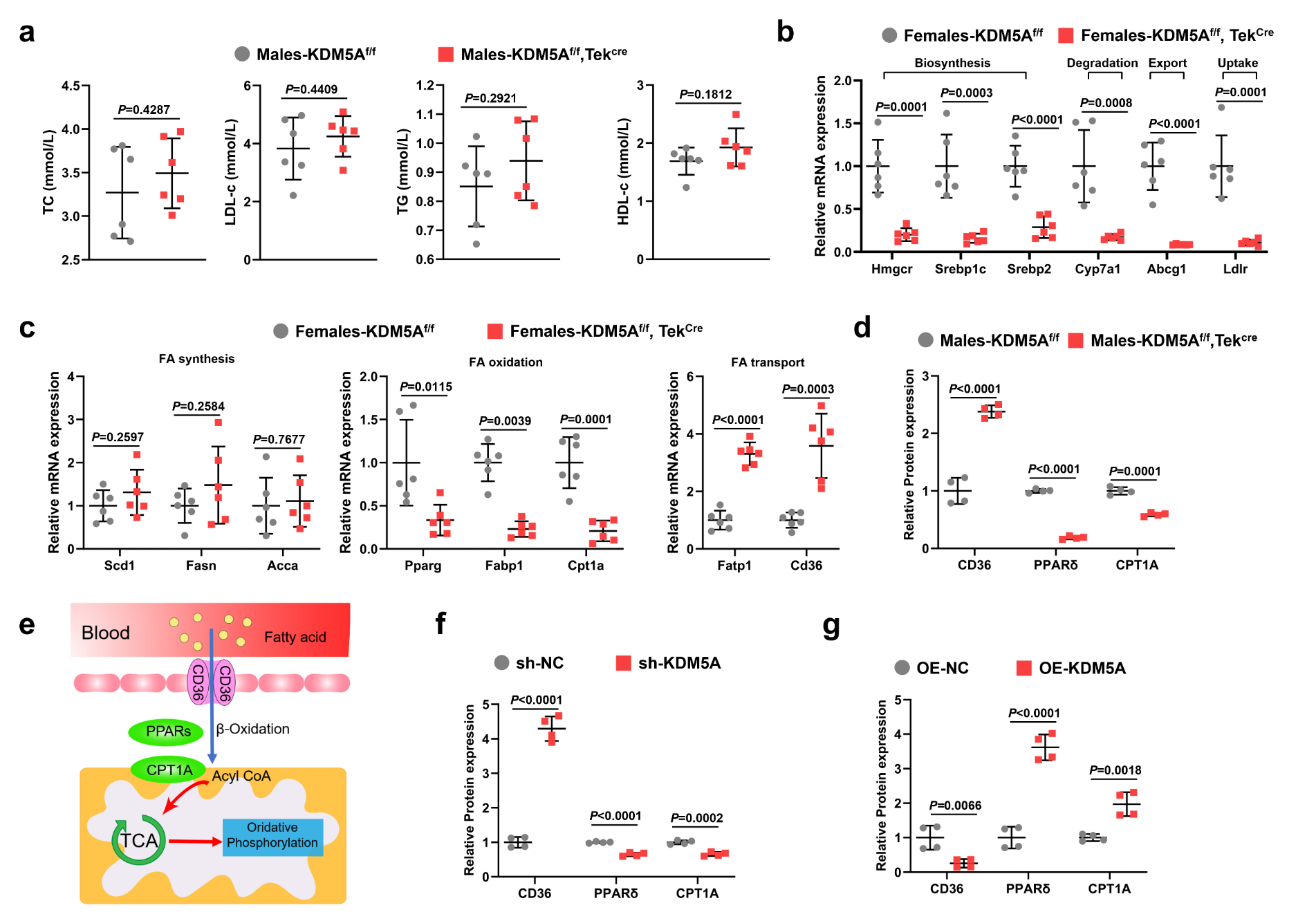
Figure S9. The impact of endothelial KDM5A deletion on cholesterol metabolism.** a) Effects of endothelial KDM5A deficiency on plasma levels of total cholesterol (TC), low-density lipoprotein-cholesterol (LDL-c), triglyceride (TG), and high-density lipoprotein-cholesterol (HDL-c). b) RT-qPCR analysis of cholesterol biosynthesis (Hmgcr, Srebp1c and Srebp2), degradation (Cyp7a1), export (Abcg1) and uptake (Ldlr; n=6) in female KDM5A^f/f^, Tek^Cre^ mice. c) RT-qPCR analysis of FA synthesis (Scd1, Fasn and Acca; Left; *n* = 6), FA transport (Pparg, Fabp1 and Cpt1a; Middle; *n* = 6), and FA oxidation (Fatp1 and Cd36; Right; *n* = 6) markers in female KDM5A^f/f^, Tek^Cre^ mice. d) Quantitative analysis of CD36, CPT1A and PPARδ proteins in the liver of males KDM5A^f/f^ and KDM5A^f/f^, Tek^Cre^ mice (*n* = 4). e) Schematic diagram of fatty acid metabolism. f) Western blot analysis of CD36, CPT1A and PPARδ in the AML-12 after VECs were transfected with sh-KDM5A (*n* = 6). g) Western blot analysis of CD36, CPT1A and PPARδ in the AML-12 after VECs were transfected with OE-KDM5A (*n* = 4). Data are displayed as mean ± SD. Unpaired 2-tailed t test was used in Figure S9.

**
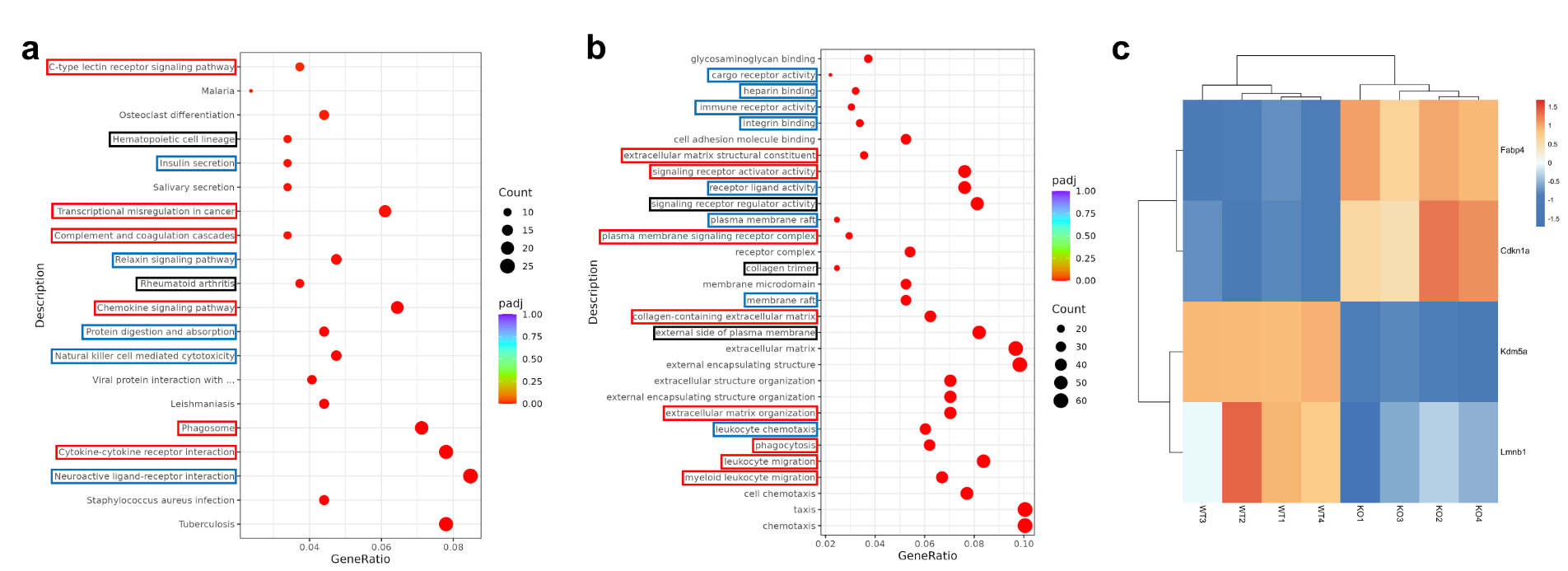
Figure S10.** **Differentially expressed genes after KDM5A knockout in VECs are mainly enriched in aging and metabolic signaling pathways.** a) GO and b) KEGG pathway enrichment of RNA-seq analysis in KDM5A^f/f^, Tek^Cre^ versus KDM5A^f/f^ mouse VECs after H_2_O_2_-induced senescence treatment [Senescence-associated pathways (red), metabolism-associated pathways (blue), and pathways simultaneously linked to both senescence and metabolism (black)]. c) Heatmap showing the expression of FABP4, KDM5A, and senescence-associated genes (Cdkn1a and Lmnb1) in senescent VECs from male KDM5A^f/f^(WT) and KDM5A^f/f^, Tek^Cre^ (KO) mice (*n* = 4).

**
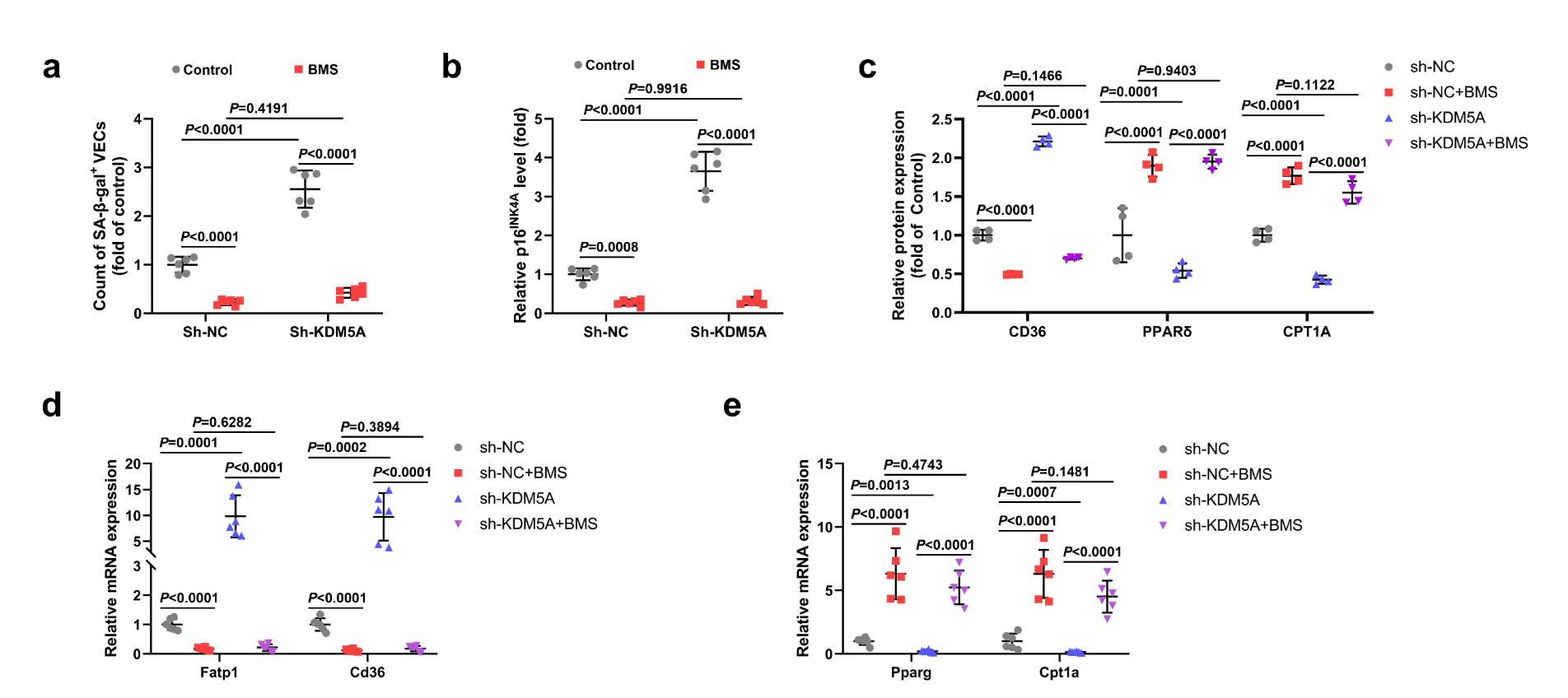
Figure S11.** **Changes of FA metabolism-related indicators in AML-12 cells.** a) Quantitative analysis of the number of senescent cells in H_2_O_2_-induced VECs after BMS treatment (*n* = 6). b) Quantitative analysis of p16^INK4α^ expression in H_2_O_2_-induced VECs after BMS treatment (*n* = 6). c) Quantitative analysis of CD36, PPARδ and CPT1A proteins in AML-12 cells after sh-KDM5A transfection and BMS treatment in upper VECs (*n* = 4). d-e) RT-qPCR analysis of FA oxidation (Fatp1 and Cd36) and FA transport (Pparg and Cpt1a) in AML-12 cells after VECs being transfected with sh-KDM5A and treated with BMS (*n* = 6). Data are presented as mean ± SD. Two-way ANOVA analysis followed by Sidak post hoc multi-comparison test was used.


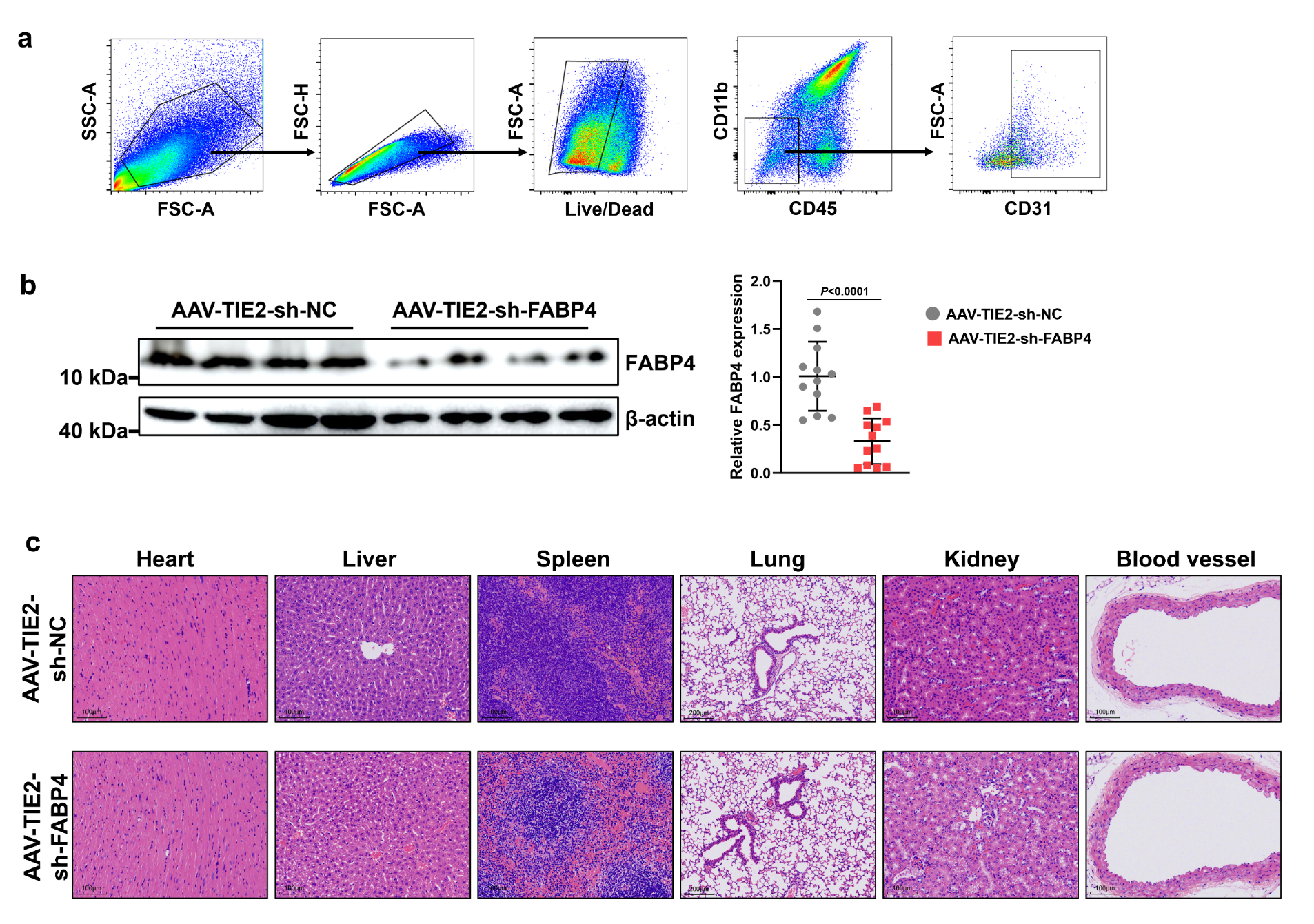


**Figure S12.** **Expression efficiency of AAV-TIE2-sh-*FABP4* in VECs and its toxicity in mice.** a) Flow sorting strategy for liver VECs in 6-week-old C57BL/6J mice treated with AAV-TIE2-sh-*FABP4* or AAV-TIE2-sh-*NC*. b) Western blots analysis of FABP4 expression efficiency in VECs after AAV-TIE2-sh-*FABP4* treatment (*n* = 12). c) H&E staining of the heart, liver, spleen, lung, kidney, and blood vessels of mice after 2 weeks of AAV-TIE2-sh-*FABP4* treatment, Scale bar: 100 or 200 μm. Data are displayed as mean ± SD. Unpaired 2-tailed t test was used in Figure S12b.


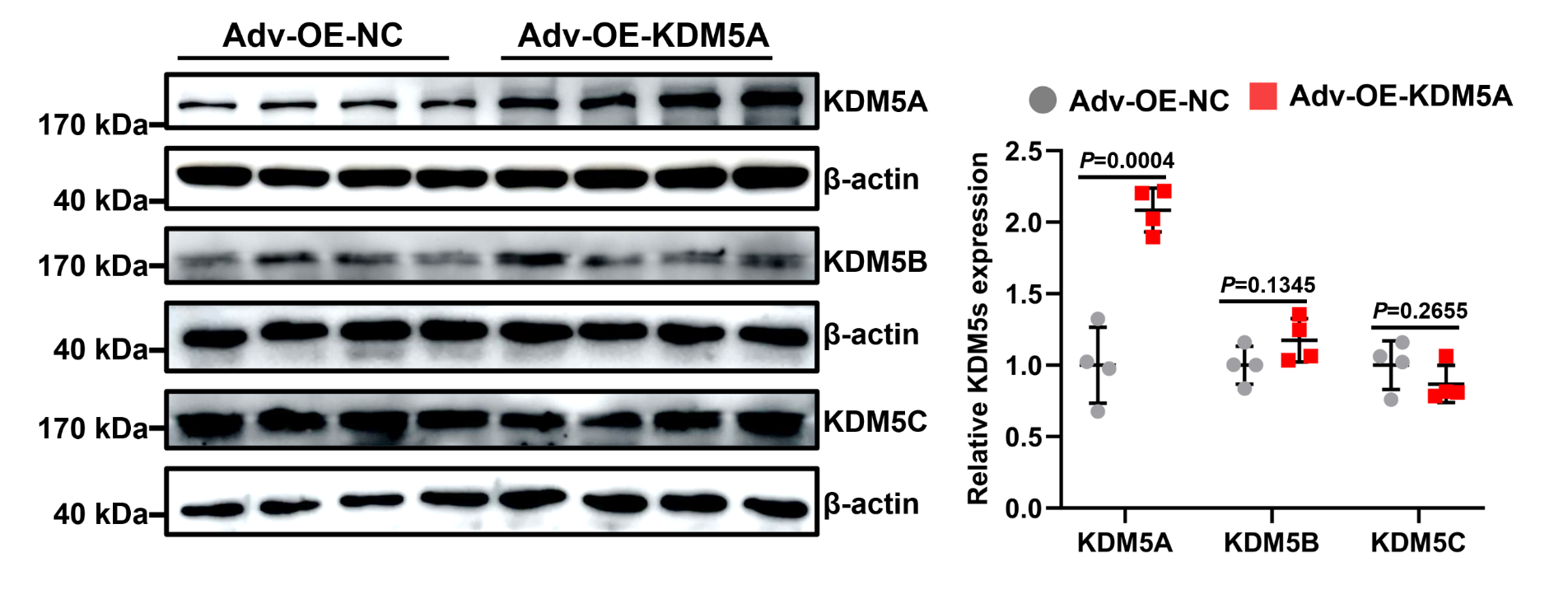


**Figure S13. Verification of off-target effects after Adv-OE-KDM5A treatment.** Western blot analysis of KDM5A/B/C expression efficiency in liver VECs after Adv-OE-*KDM5A* or Adv-OE-*NC* treatment (*n* = 4; each sample pooled from 3 mice). Data are displayed as mean ± SD. Unpaired 2-tailed t test was used in Figure S13.


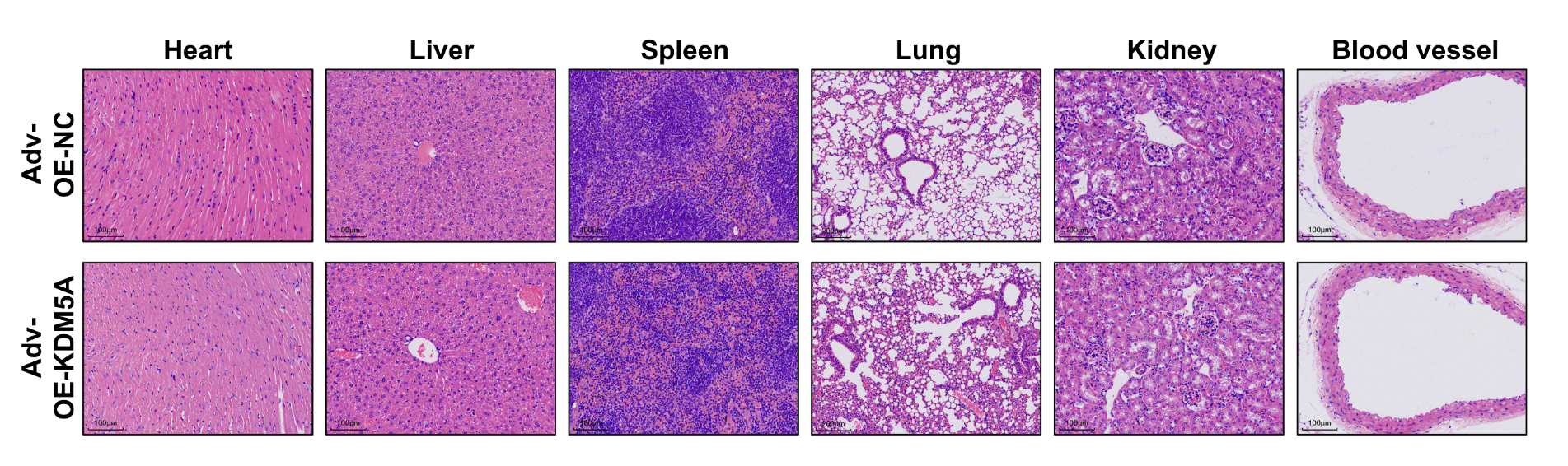


**Figure S14. Evaluation of toxic damage by Adv-OE-KDM5A treatment.** H&E staining of heart, liver, spleen, lung, kidney, and blood vessels after 2 weeks of Adv-OE-*KDM5A* or Adv-OE-*NC* treatment, Scale bar: 100 or 200 μm.


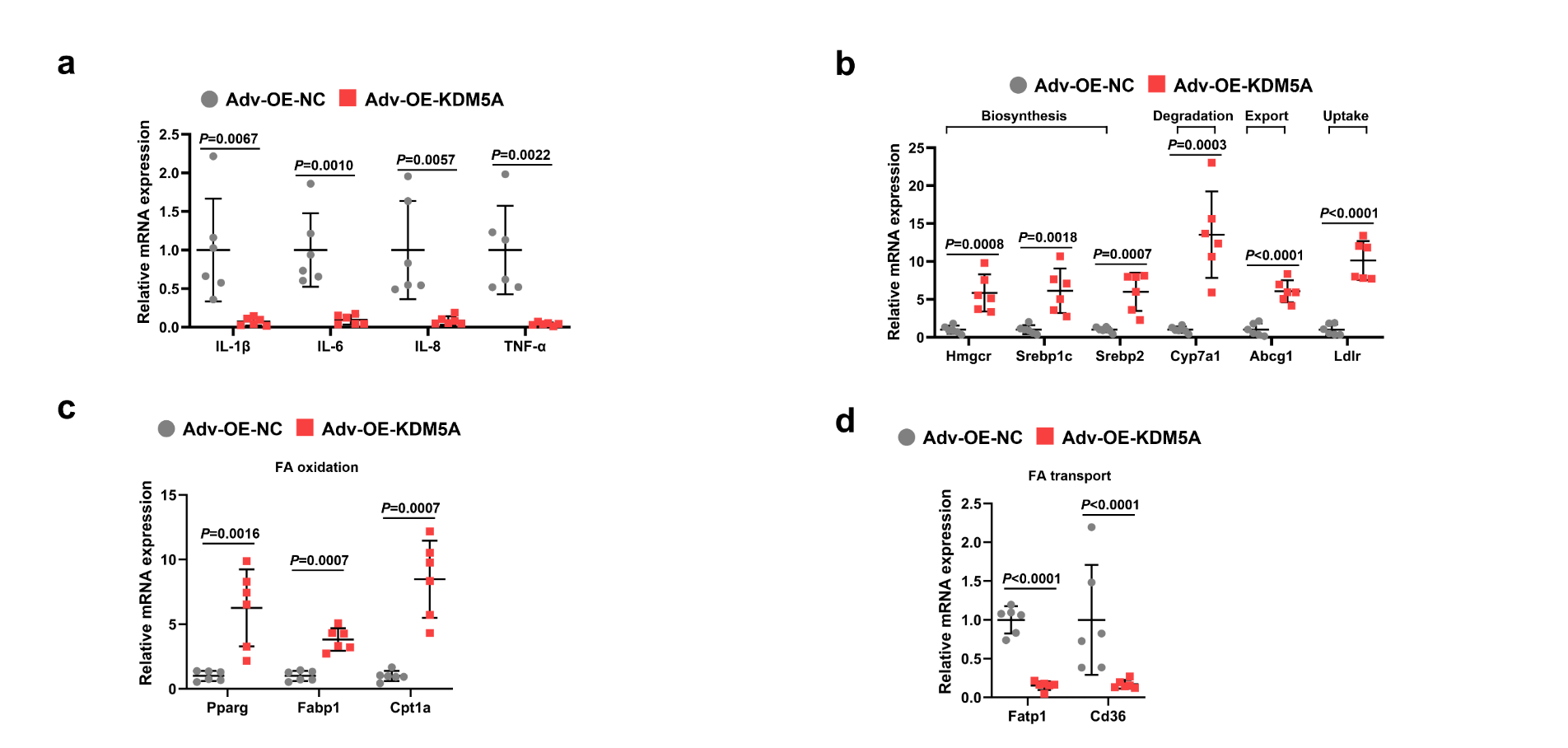


**Figure S15. Adv-OE-KDM5A treatment changes the transcriptional levels of SASPs as well as cholesterol and FA metabolism-related markers.** a) RT-qPCR analysis of SASPs (IL-1β, IL-6, IL-8, and TNF-α) in liver VECs from 24-month-old male mice after Adv-OE-KDM5A treatment (*n* = 6). b), c) and d) RT-qPCR analysis of cholesterol biosynthesis (Hmgcr, Srebp1c and Srebp2), cholesterol degradation (Cyp7a1), cholesterol export (Abcg1), cholesterol uptake (Ldlr), FA synthesis (Scd1, Fasn and Acca), FA transport (Pparg, Fabp1 and Cpt1a), and FA oxidation (Fatp1 and Cd36) markers (*n* = 6). Data are presented as mean ± SD. One-way ANOVA analysis followed by Sidak post hoc multi-comparison test was used in these data.


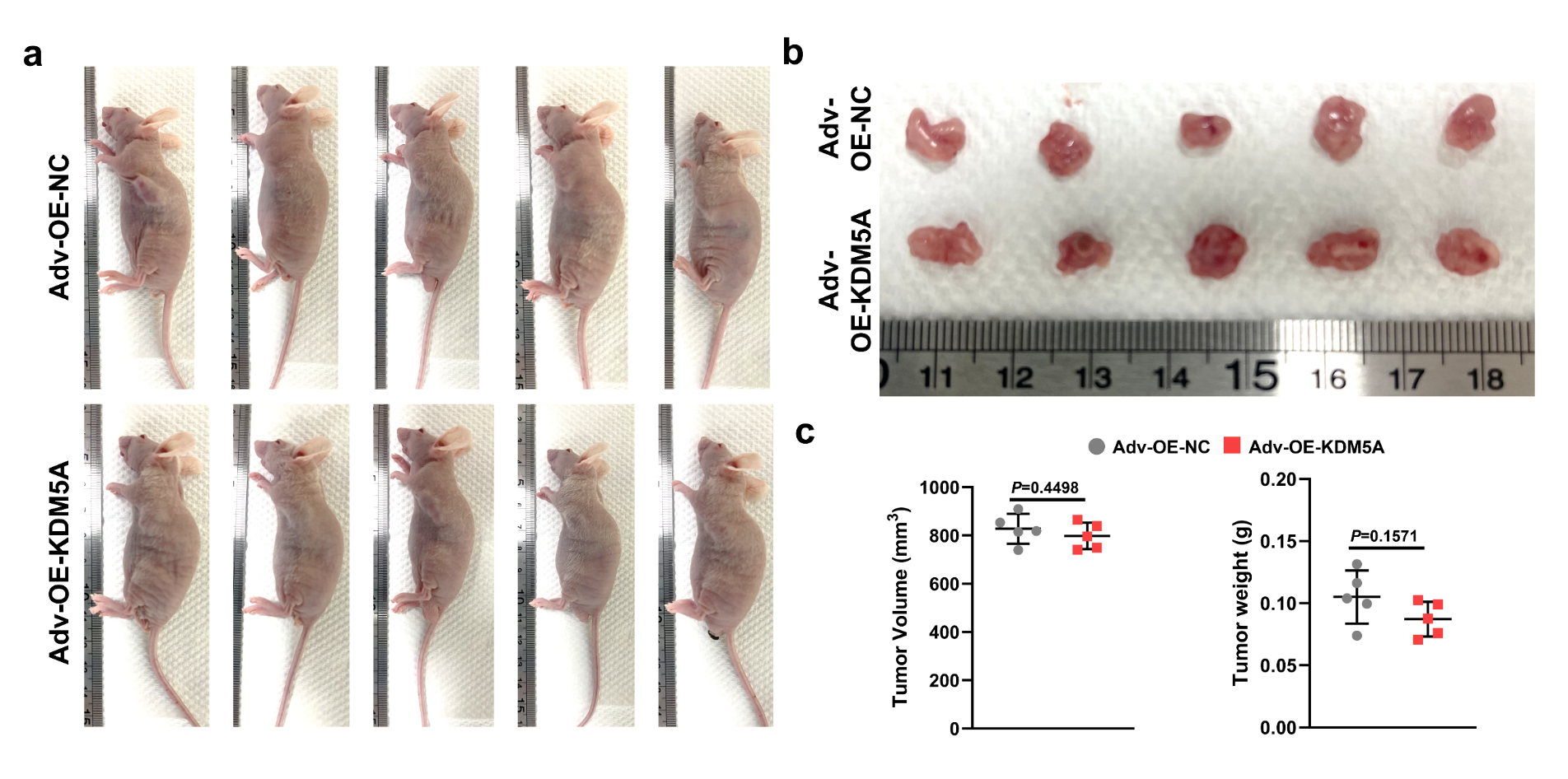


**Figure S16. Effects of Adv-OE-KDM5A treatment on tumors.** a) and b) Photographs of A549 non-small cell lung cancer tumors after treatment with Adv-OE-*NC* or Adv-OE-*KDM5A*. c) Changes in tumor volumes (left) and weights (right) after treatment with Adv-OE-*NC* or Adv-OE-*KDM5A* (*n* = 5). Data are displayed as mean ± SD. Unpaired 2-tailed t test was used in Figure S13c.

**References**

[1] M. Grunewald, S. Kumar, H. Sharife, E. Volinsky, A. Gileles-Hillel, T. Licht, A. Permyakova, L. Hinden, S. Azar, Y. Friedmann, P. Kupetz, R. Tzuberi, A. Anisimov, K. Alitalo, M. Horwitz, S. Leebhoff, O. Z. Khoma, R. Hlushchuk, V. Djonov, R. Abramovitch, J. Tam, E. Keshet, *Science* **2021**, *373*, eabc8479.

[2] L. R. Hoving, M. Heijink, V. van Harmelen, K. W. van Dijk, M. Giera, *Methods. Mol. Biol.* **2018**, *1730*, 257.

[3] M. Beccaria, F. A. Franchina, M. Nasir, T. Mellors, J. E. Hill, G. Purcaro, *Anal. Bioanal. Chem.* **2018**, *410*, 7987.

[4] a) H. Oeseburg, D. Iusuf, P. van der Harst, W. H. van Gilst, R. H. Henning, A. J. Roks, *Hypertension* **2009**, *53*, 417; b) T. Lin, W. Q. Yang, W. W. Luo, L. L. Zhang, Y. Q. Mai, Z. Q. Li, S. T. Liu, L. J. Jiang, P. Q. Liu, Z. M. Li, *Oxid. Med. Cell. Longev.* **2022**, *2022*, 1198607.

[5] R. Menghini, V. Casagrande, M. Cardellini, E. Martelli, A. Terrinoni, F. Amati, M. Vasa-Nicotera, A. Ippoliti, G. Novelli, G. Melino, R. Lauro, M. Federici, *Circulation* **2009**, *120*, 1524.

**Supplementary Table 1**

Mouse primers used in this study

| **Gene** | **Forward primers (5’–3’)** | **Reverse primers (5’–3’)** |
| --- | --- | --- |
| p16 | aatctccgcgaggaaagc | gtctgcagcggactccat |
| p21 | GGGCGCACGATGTTCAGAA | CACCACCAGGTCGAAATGGG |
| Srebp1c | TGACCCGGCTATTCCGTGA | CTGGGCTGAGCAATACAGTTC |
| FABP4 | AAGGTGAAGAGCATCATAACCCT | TCACGCCTTTCATAACACATTCC |
| KDM5A | CACAGACCCGCTGAGTTTTAT | CTTCACAGGCAAATGGAGGTT |
| KDM5B | CTGGGAAGAGTTCGCGGAC | CGCGGGGTGAAATGAAGTTTAT |
| KDM5C | GAGGCCCAGACAAGAGTGAAA | TTGGGAATCTTTAAGGATGAGCC |
| KDM5D | CCAGGATCTGACGACTTTCTACC | TTCTCCGCAATGGGTCTGATT |
| IL-1β | GCAACTGTTCCTGAACTCAACT | ATCTTTTGGGGTCCGTCAACT |
| IL-6 | TAGTCCTTCCTACCCCAATTTCC | TTGGTCCTTAGCCACTCCTTC |
| IL-8 | CAAGGCTGGTCCATGCTCC | TGCTATCACTTCCTTTCTGTTGC |
| TNF-α | CCCTCACACTCAGATCATCTTCT | GCTACGACGTGGGCTACAG |
| Hmgcr | AGCTTGCCCGAATTGTATGTG | TCTGTTGTGAACCATGTGACTTC |
| Srebp2 | GCAGCAACGGGACCATTCT | CCCCATGACTAAGTCCTTCAACT |
| Cyp7a1 | GGGATTGCTGTGGTAGTGAGC | GGTATGGAATCAACCCGTTGTC |
| Abcg1 | CTTTCCTACTCTGTACCCGAGG | CGGGGCATTCCATTGATAAGG |
| Ldlr | TGACTCAGACGAACAAGGCTG | ATCTAGGCAATCTCGGTCTCC |
| Scd1 | TTCTTGCGATACACTCTGGTGC | CGGGATTGAATGTTCTTGTCGT |
| Fasn | GGAGGTGGTGATAGCCGGTAT | TGGGTAATCCATAGAGCCCAG |
| Acca | GACGCATTCCTGTGCTGAC | TGGACTCCTGGGGCTCTTTTA |
| Pparg | TCGCTGATGCACTGCCTATG | GAGAGGTCCACAGAGCTGATT |
| Fabp1 | ATGAACTTCTCCGGCAAGTACC | CTGACACCCCCTTGATGTCC |
| Cpt1a | CTCCGCCTGAGCCATGAAG | CACCAGTGATGATGCCATTCT |
| Fatp1 | CGCTTTCTGCGTATCGTCTG | GATGCACGGGATCGTGTCT |
| Cd36 | ATGGGCTGTGATCGGAACTG | GTCTTCCCAATAAGCATGTCTCC |
| β-actin | GGCTGTATTCCCCTCCATCG | CCAGTTGGTAACAATGCCATGT |
