# Supplementary material for: Targeting Endothelial KDM5A to Attenuate Aging and Ameliorate Age‐Associated Metabolic Abnormalities
Source: Adv Sci (Weinh). 2025 Nov 14;13(6):e12657. doi: 10.1002/advs.202512657 (PMC12866742; doi:10.1002/advs.202512657)

Supplementary Material 1

KDM5A knockdown plasmid construction

Experimental Principle:

RNA interference (RNAi) is a highly conserved biological process across evolution, where double-stranded RNA (dsRNA) induces the efficient and specific degradation of homologous mRNA. This technology, by allowing for the specific reduction or silencing of target gene expression, has become a powerful tool in exploring gene function, as well as in the fields of gene therapy for infectious diseases and malignant tumors.

Short hairpin RNA (shRNA) is one method of RNA interference. It utilizes RNA polymerase III (pol III) promoters, such as the human and murine U6 and H1 promoters, to transcribe short interfering RNA (siRNA), which are double-stranded RNA molecules of 19-21 nucleotides in length. Within the cell, transcribed shRNA is processed and incorporated into the RNA-induced silencing complex (RISC), which guides the degradation of target RNA by nucleases.

The advantages of using shRNA expression vectors include their suitability for long-term studies. Vectors with antibiotic selection markers can continuously suppress target gene expression in cells for weeks or even longer. Additionally, viral vectors can be employed to express siRNA, offering the advantage of directly infecting cells with high efficiency for gene silencing studies. This method circumvents the challenges associated with low transfection efficiency of plasmids and provides more stable transfection outcomes.

Below is the map of the selected interference vector, **pADV-U6-shRNA-CMV-MCS**, which shows where the shRNA sequence to be constructed will be inserted.

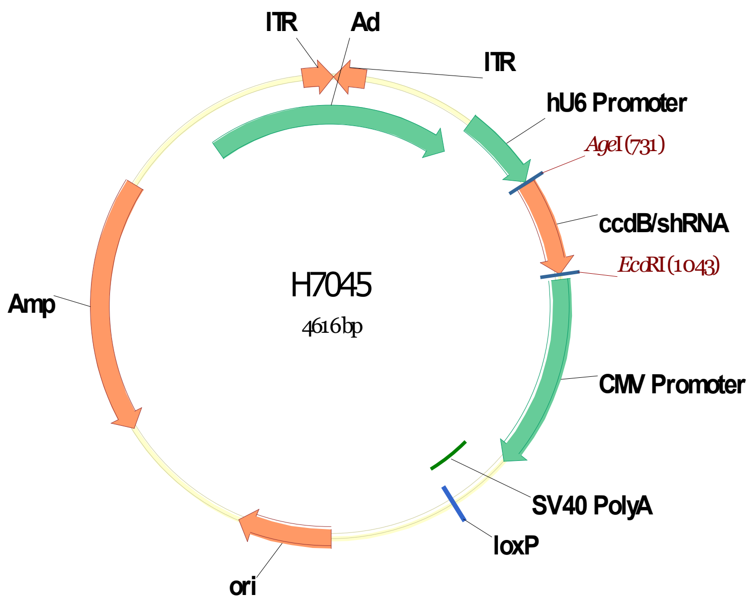

Experimental purpose:

To construct a viral vector with mouse Kdm5a interference.

Experimental steps:

Design siRNA targets based on the transcript of mouse Kdm5a gene and arrange primer synthesis. Anneal the single-stranded primer into a double-stranded oligo sequence, connect it into the double-enzyme linearized RNA interference vector, and replace the original ccdB toxic gene. Screen the transformants by colony PCR, and sequence the screened positive clones. Sequencing verifies the correct clones and extracts high-purity plasmids.

Experimental Results:

1. siRNA sequence:

siRNA sequences

Marker	Gene	Gene ID	TargetSeq	GC%
Y13684	Kdm5a	NM_145997.2	CGAGGAAATAATGAGGATAAA	
Y13685	Kdm5a	NM_145997.2	ACTACCAATGGAGGATCTTAA	
Y13686	Kdm5a	NM_145997.2	TGTTGGTGTATCCGCAGAAAT	
Y5068	NC2		CCTAAGGTTAAGTCGCCCTCG	

Viral vector construction framework

NO.	5′	STEM	Loop	STEM	3′
Y13684-F	Ccgg	CGAGGAAATAATGAGGATAAA	CTCGAG	TTTATCCTCATTATTTCCTCG	TTTTTTg
Y13684-R	aattcaaaaaa	CGAGGAAATAATGAGGATAAA	CTCGAG	TTTATCCTCATTATTTCCTCG	
Y13685-F	Ccgg	ACTACCAATGGAGGATCTTAA	CTCGAG	TTAAGATCCTCCATTGGTAGT	TTTTTTg
Y13685-R	aattcaaaaaa	ACTACCAATGGAGGATCTTAA	CTCGAG	TTAAGATCCTCCATTGGTAGT	
Y13686-F	Ccgg	TGTTGGTGTATCCGCAGAAAT	CTCGAG	ATTTCTGCGGATACACCAACA	TTTTTTg
Y13686-R	aattcaaaaaa	TGTTGGTGTATCCGCAGAAAT	CTCGAG	ATTTCTGCGGATACACCAACA	
Y5068-F	Ccgg	CCTAAGGTTAAGTCGCCCTCG	TTCAAGAGA	CGAGGGCGACTTAACCTTAGG	TTTTTTg
Y5068-R	aattcaaaaaa	CCTAAGGTTAAGTCGCCCTCG	TCTCTTGAA	CGAGGGCGACTTAACCTTAGG	

2. DNA primer fragment:

Y13684-F	CcggCGAGGAAATAATGAGGATAAACTCGAGTTTATCCTCATTATTTCCTCGTTTTTTg
Y13684-R	aattcaaaaaaCGAGGAAATAATGAGGATAAACTCGAGTTTATCCTCATTATTTCCTCG
Y13685-F	CcggACTACCAATGGAGGATCTTAACTCGAGTTAAGATCCTCCATTGGTAGTTTTTTTg
Y13685-R	aattcaaaaaaACTACCAATGGAGGATCTTAACTCGAGTTAAGATCCTCCATTGGTAGT
Y13686-F	CcggTGTTGGTGTATCCGCAGAAATCTCGAGATTTCTGCGGATACACCAACATTTTTTg
Y13686-R	aattcaaaaaaTGTTGGTGTATCCGCAGAAATCTCGAGATTTCTGCGGATACACCAACA
Y5068-F	CcggCCTAAGGTTAAGTCGCCCTCGCTCGAGCGAGGGCGACTTAACCTTAGGTTTTTTg
Y5068-R	aattcaaaaaaCCTAAGGTTAAGTCGCCCTCGCTCGAGCGAGGGCGACTTAACCTTAGG

3. Linearization of expression vector:

Use endonuclease to cut the expression vector to obtain the vector fragment.

4. Colony PCR to identify positive clones:

Use colony PCR to identify transformants.

5. Analysis of sequencing results:

Y13684 sequencing results:

AATTAGTACAAAATACGTGACGTAGAAAGTAATAATTTCTTGGGTAGTTTGCAGT
TTTAAAATTATGTTTTAAAATGGACTATCATATGCTTACCGTAACTTGAAAGTATTTCG
ATTTCTTGGCTTTATATATCTTGTGGAAAGGACGAAACACCGGCGAGGAAATAATGA
GGATAAACTCGAGTTTATCCTCATTATTTCCTCGTTTTTGAATTCGGATCCATTAGGC
GGCCGCGTGGATAACCGTATTACCGCCATGCATTAGTTATTAATAGTAATCAATTACG
GGGTCATTAGTTCATAGCCCATATATGGAGTTCCGCGTTACATAACTTACGGTAAATG
GCCCGCCTGGCTGACCGCCCAACGACCCCCGCCCATTGACGTCAATAATGACGTATGT
TCCCATAGTAACGCCAATAGGGACTTTCCATTGACGTCAATGGGTGGAGTATTTACGG
TAAACTGCCCACTTGGCAGTACATCAAGTGTATCATATGCCAAGTACGCCCCCTATTG
ACGTCAATGACGGTAAATGGCCCGCCTGGCATTATGCCCAGTACATGACCTTATGGG
ACTTTCCTACTTGGCAGTACATCTACGTATTAGTCATCGCTATTACCATGGTGATGCG
GTTTTGGCAGTACATCAATGGGCGTGGATAGCGGTTTGACTCACGGGGATTTCCAAGT
CTCCACCCCATTGACGTCA

Y13684-F:

CcggCGAGGAAATAATGAGGATAAACTCGAGTTTATCCTCATTATTTCCTCGTTTTTg

Y13685 sequencing results:

CTTGGATTATTTGACTGTAACACAAAGATATTAGTACAAAATACGTGACGTAGAAA
GTAATAATTTCTTGGGTAGTTTGCAGTTTTAAAATTATGTTTTAAAATGGACTATCATATG
CTTACCGTAACTTGAAAGTATTTCGATTTCTTGGCTTTATATATCTTGTGGAAAGGACGA
AACACCGGACTACCAATGGAGGATCTTAACTCGAGTTAAGATCCTCCATTGGTAGTTTT
TTTGAATTCGGATCCATTAGGCGGCCGCGTGGATAACCGTATTACCGCCATGCATTAGTT
ATTAATAGTAATCAATTACGGGGTCATTAGTTCATAGCCCATATATGGAGTTCCGCGTTAC
ATAACTTACGGTAAATGGCCCGCCTGGCTGACCGCCCAACGACCCCCGCCCATTGACG
TCAATAATGACGTATGTTCCCATAGTAACGCCAATAGGGACTTTCCATTGACGTCAATGG
GTGGAGTATTTACGGTAAACTGCCCACTTGGCAGTACATCAAGTGTATCATATGCCAAG
TACGCCCCCTATTGACGTCAATGACGGTAAATGGCCCGCCTGGCATTATGCCCAGTACA
TGACCTTATGGGACTTTCCTACTTGGCAGTACATCTACGTATTAGTCATCGCTATTACCAT
GGTGATGCGGTTTTG

Y13685-F:

CcggACTACCAATGGAGGATCTTAACTCGAGTTAAGATCCTCCATTGGTAGTTTTTTg

Y13686 sequencing results:

TGACTGTAACACAAAGATATTAGTACAAAATACGTGACGTAGAAAGTAATAATTTC
TTGGGTAGTTTGCAGTTTTAAAATTATGTTTTAAAATGGACTATCATATGCTTACCGTAA
CTTGAAAGTATTTCGATTTCTTGGCTTTATATATCTTGTGGAAAGGACGAAACACCGGT

GTTGGTGTATCCGCAGAAATCTCGAGATTTCTGCGGATACACCAACATTTTTTGAATTC
GGATCCATTAGGCGGCCGCGTGGATAACCGTATTACCGCCATGCATTAGTTATTAATAGT
AATCAATTACGGGGTCATTAGTTCATAGCCCATATATGGAGTTCCGCGTTACATAACTTA
CGGTAAATGGCCCGCCTGGCTGACCGCCCAACGACCCCCGCCCATTGACGTCAATAAT
GACGTATGTTCCCATAGTAACGCCAATAGGGACTTTCCATTGACGTCAATGGGTGGAGT
ATTTACGGTAAACTGCCCACTTGGCAGTACATCAAGTGTATCATATGCCAAGTACGCCC
CCTATTGACGTCAATGACGGTAAATGGCCCGCCTGGCATTATGCCCAGTACATGACCTT
ATGGGACTTTCCTACTTGGCAGTACATCTACGTATTAGTCATCGCTATTACCATGGTGAT
GCGGTTTTGGCAGTACATCAATGGGCGTGGATAGC

Y13686-F:
CcggTGTTGGTGTATCCGCAGAAATCTCGAGATTTCTGCGGATACACCAACATTTTTTg

Sequencing results of Y5068 (NC control plasmid containing an irrelevant sequence):

CGCGGCGCCTAATGGATCCGAATTCAAAAAACCTAAGGTTAAGTCGCCCTCGCTC
GAGCGAGGGCGACTTAACCTTAGGCCGGTGTTTCGTCCTTTCCACAAGATATATAAAGC
CAAGAAATCGAAATACTTTCAAGTTACGGTAAGCATATGATAGTCCATTTTAAAACATA
ATTTTAAAACTGCAAACTACCCAAGAAATTATTACTTTCTACGTCACGTATTTTGTACTA
ATATCTTTGTGTTTACAGTCAAATTAATTCCAATTATCTCTCTAACAGCCTTGTATCGTAT
ATGCAAATATGAAGGAATCATGGGAAATAGGCCCTCGGTGAAGGGGGCGGCCGCTCGA
GTCTAGAGCTGACTATAATAATAAAACGCCAACTTTGACCCGGAACGCGGAAAACACC
TGAGAAAAACACCTGGGCGAGTCTCCACGTAAACGGTCAAAGTCCCCGCGGCCCTAG
ACAAATATTACGCGCTATGAGTAACACAAAATTATTCAGATTTCACTTCCTCTTATTCAG
TTTTCCCGCGAAAATGGCCAAATCTTACTCGGTTACGCCCAAATTTACTACAACATCCG
CCTAAAACCGCGCGAAAATTGTCACTTCCTGTGTACACCGGCGCACACCAAAAACGTC
ACTTTTGCCACATCCGTCGCTTACATGTGTTCCGCCACACTTGCAACATCACACTTCCG
CCACACTACTACGTCACCCGCCCCG

Supplementary Material 2

KDM5A overexpression plasmid (H18662) construction

1. Sample information

**Clone serial number**	H18662	**Gene name**	Kdm5a	
**GenBank ID**	NM_145997.2	**Gene size**	5088	
**Species**	Mouse	**Upstream and downstream cloning restriction sites**	EcoRI	BamHI
**Prokaryotic resistance ：**	Amp			
**Empty vector name**	H225 pADV-mCMV-MCS-3xFLAG			
**Build Name**	pADV-mCMV-Kdm5a-3xFLAG			
**Forward sequencing primer**	MCMV-F GGTATAAGAGGCGCGACCAG			
**Reverse sequencing primer**	SV40-pArev GAAATTTGTGATGCTATTGC			

2. Empty vector map (before inserting the target gene)

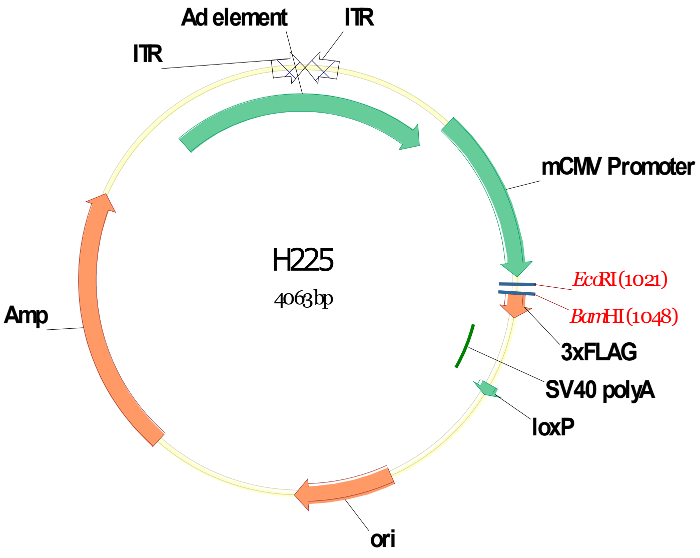

3. Full Spectrum

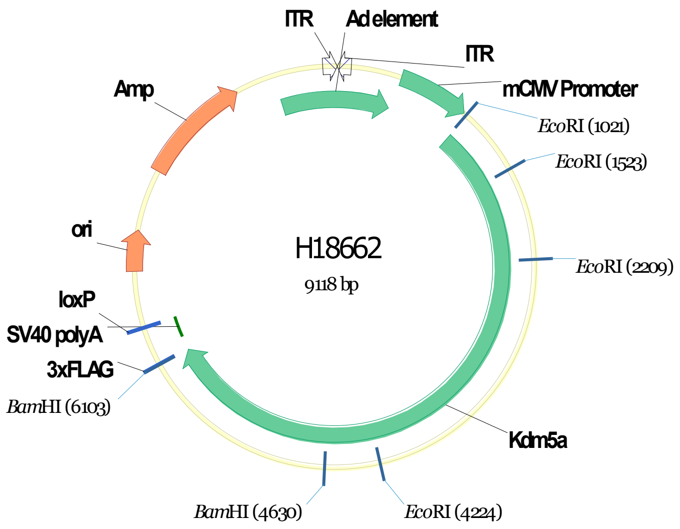

4. Experimental Results

4.1 Sample information

Serial number	Sample name	Carrier information
1	Marker	
2	293T	
3	H225	pADV-mCMV-MCS-3xFLAG
4	H18662	pADV-mCMV-Kdm5a-3xFLAG

4.2 Results

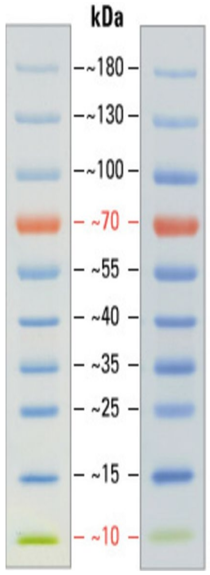

Figure 1. Protein Marker

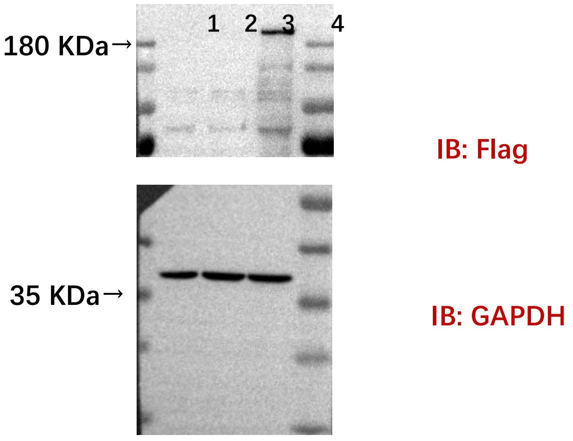

Figure 2. Western Blot results

Supplementary Material 3

Mouse Kdm5a Conditional Knockout Project (CRISPR/Cas)

1.Objective

To create a Kdm5a conditional knockout mouse model (C57BL/6NCya) by CRISPR/Cas-mediated genome engineering.

2. Project Summary

The Kdm5a gene (NCBI Reference Sequence: NM_145997; Ensembl: ENSMUSG00000030180) is located on mouse chromosome 6. Twenty-eight exons are identified, with the ATG start codon in exon 1 and the TAG stop codon in exon 28 (Transcript Kdm5a-201: ENSMUST00000005108). Exon 5~6 will be selected as conditional knockout region (cKO region). The region contains 241 bp coding sequence. Deletion of this region should result in the loss of function of the mouse Kdm5a gene. To engineer the targeting vector, homologous arms and cKO region will be generated by PCR using BAC clone RP23-393D21 as template. Ribonucleoprotein (RNP) and targeting vector will be co-injected into fertilized eggs for cKO mouse production. The pups will be genotyped by PCR followed by sequencing analysis.

Note: Mice homozygous for a knock-out allele exhibit reduced body size, abnormal involuntary movement and quantitative changes in the hematopoietic stem cell and myeloid progenitor compartments, consistent with enhanced survival and increased cycling. Neonatal survival is sensitive to genetic background.

The knockout of Exon 5~6 will result in frameshift of the gene, and covers 4.75% of the coding region. The size of intron 4 for 5'-loxP site insertion: 6547 bp, and the size of intron 6 for 3'-loxP site insertion: 2599 bp. The size of effective cKO region: ~2.0 kb. This strategy is designed based on genetic information in existing databases. Due to the complexity of biological processes, all risk of loxP insertion on gene transcription, RNA splicing and protein translation cannot be predicted at existing technological level. It is possible to adjust the target region according to the actual situation during the experiment.

3. Overview of the Targeting Strategy

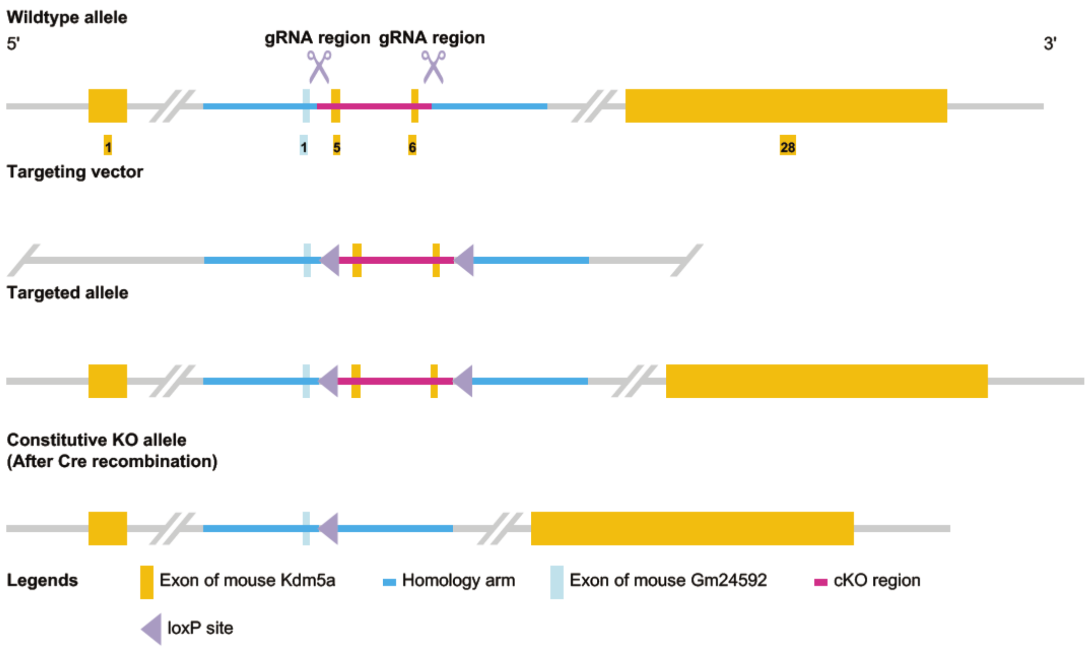

4. Sequence Analysis

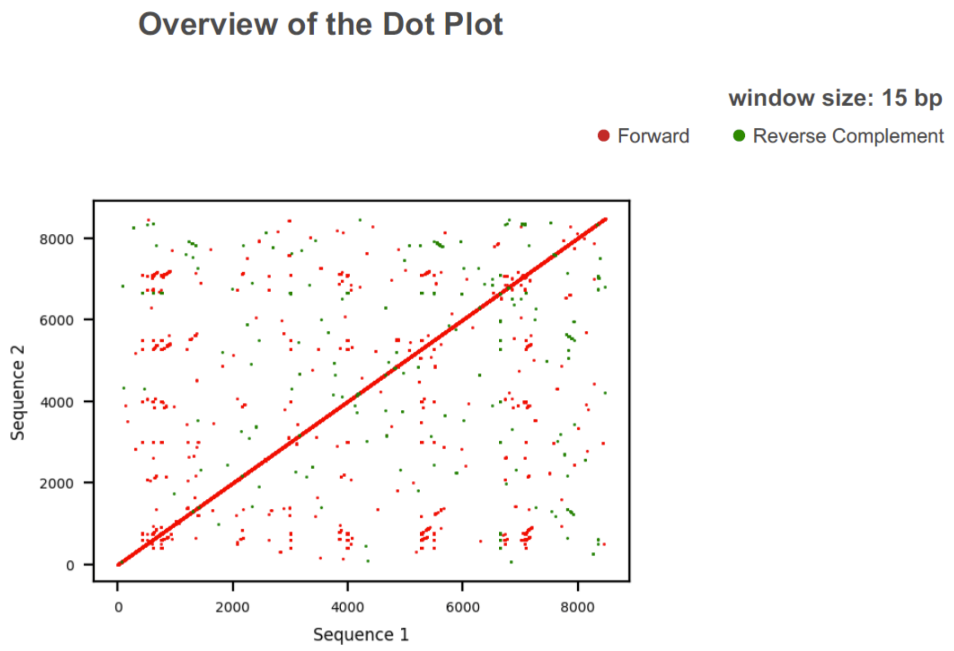

Note: The sequence of homologous arms and cKO region is aligned with itself to determine if there are tandem repeats. Tandem repeats are found in the dot plot matrix. It may be difficult to construct this targeting vector.

Overview of the GC Content Distribution

window size: 300 bp

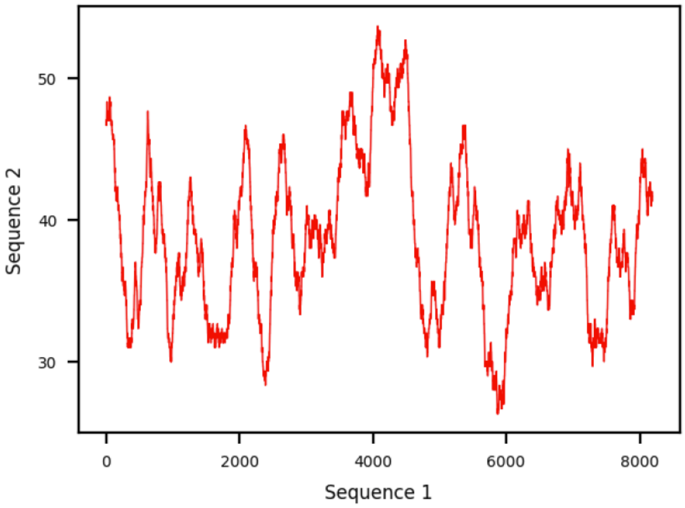

Full Length(8488bp) | A(26.5% 2249) | C(19.24% 1633) | G(19.64% 1667) | T(34.63% 2939)

Note: The sequence of homologous arms and cKO region is analyzed to determine the GC content. No significant high GC-content region is found. So this region is suitable for PCR screening or sequencing analysis.

BLAT Search Results (up)

Note: The 3000 bp section upstream of cKO region is BLAT searched against the genome. No significant similarity is found.

Query	Score	Start	End	Qsize	Identity	Chrom	Strand	Start	End	Span
YourSeq	3000	1	3000	3000	100.0%	chr6	+	120355245	120358244	3000
YourSeq	264	505	1141	3000	91.1%	chr10	-	24508084	24508700	617
YourSeq	169	507	677	3000	99.5%	chr7	-	37952527	37952697	171
YourSeq	165	507	676	3000	98.9%	chr10	-	93065566	93065753	188
YourSeq	164	507	676	3000	98.3%	chr8	-	34094438	34094607	170
YourSeq	162	503	674	3000	97.7%	chr5	-	92661210	92661401	192
YourSeq	161	507	676	3000	97.7%	chr4	-	142241820	142242002	183
YourSeq	161	507	677	3000	96.5%	chr16	-	15424474	15424643	170
YourSeq	160	507	674	3000	98.3%	chr2	-	131648060	131648265	206
YourSeq	155	518	676	3000	98.8%	chr9	+	106722959	106723117	159

BLAT Search Results (down)

Note: The 3000 bp section downstream of cKO region is BLAT searched against the genome. No significant similarity is found.

Query	Score	Start	End	Qsize	Identity	Chrom	Strand	Start	End	Span
YourSeq	3000	1	3000	3000	100.0%	chr6	+	120360233	120363232	3000
YourSeq	306	40	442	3000	95.8%	chr13	+	100746973	100747581	609
YourSeq	303	39	441	3000	92.1%	chr17	+	27743863	27744243	381
YourSeq	295	39	458	3000	94.0%	chr5	+	33033417	33033952	536
YourSeq	295	42	447	3000	94.1%	chr16	+	17239267	17239747	481
YourSeq	286	40	441	3000	90.8%	chr10	-	126868029	126868379	351
YourSeq	282	46	442	3000	89.3%	chr5	-	121701983	121702320	338
YourSeq	282	56	442	3000	91.5%	chr19	-	6988656	6988990	335
YourSeq	275	69	442	3000	94.3%	chr9	+	110232263	110232821	559
YourSeq	273	42	435	3000	91.3%	chr7	+	81131913	81132217	305

Gene Summary

Kdm5a:

Enables chromatin DNA binding activity and transcription coactivator activity. Involved in circadian regulation of gene expression and negative regulation of histone deacetylase activity. Acts upstream of or within histone H3-K4 demethylation and negative regulation of transcription by RNA polymerase II. Located in nucleus. Part of protein-DNA complex. Is expressed in several structures, including 1st branchial arch; early conceptus; genitourinary system; limb; and sensory organ. Human ortholog(s) of this gene implicated in ankylosing spondylitis. Orthologous to human KDM5A (lysine demethylase 5A).

5.1 Gene Alignment

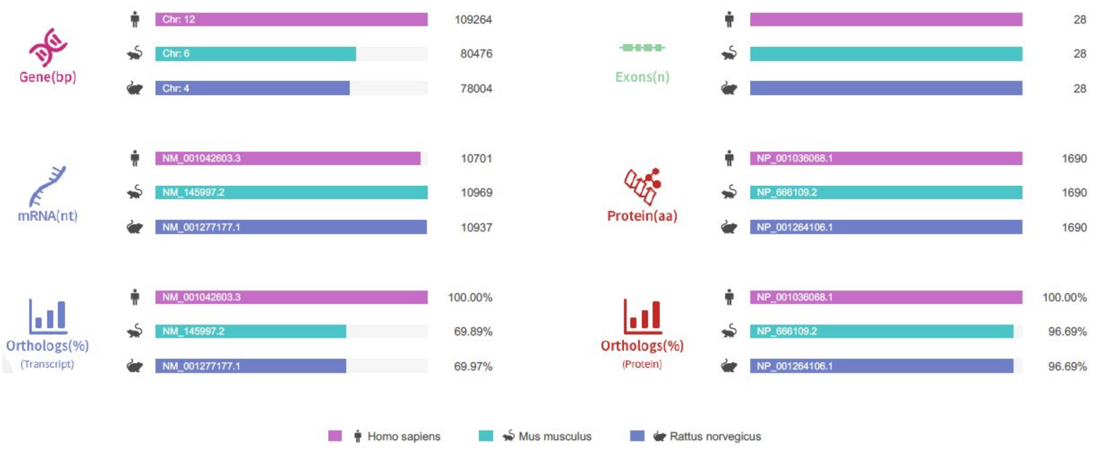

5.2 Paralogous Gene

Target Gene	Paralogous Gene	Location	Target % ID	Query % ID
Kdm5a	Kdm5b (lysine (K)-specific demethylase 5B) ENSMUSG00000042207	Chr 1: 134487909 - 134563023:1	52.14 %	47.63 %
Kdm5a	Kdm5c (lysine (K)-specific demethylase 5C) ENSMUSG00000025332	Chr X: 151016016 - 151057531:1	51.87 %	47.69 %
Kdm5a	Kdm5d (lysine (K)-specific demethylase 5D) ENSMUSG00000056673	Chr Y: 897788 - 956786:1	49.94 %	45.74 %
Kdm5a	Kdm4d (lysine (K)-specific demethylase 4D) ENSMUSG00000053914	Chr 9: 14373844 - 14411778:-1	25.88 %	7.81 %
Kdm5a	Kdm4c (lysine (K)-specific demethylase 4C) ENSMUSG00000028397	Chr 4: 74160734 - 74324097:1	20.49 %	12.78 %
Kdm5a	Kdm4a (lysine (K)-specific demethylase 4A) ENSMUSG00000033326	Chr 4: 117994154 - 118037240:-1	19.74 %	12.43 %
Kdm5a	Kdm4b (lysine (K)-specific demethylase 4B) ENSMUSG00000024201	Chr 17: 56633062 - 56709870:1	18.97 %	12.19 %
Kdm5a	Jarid2 (jumonji, AT rich interactive domain 2) ENSMUSG00000038518	Chr 13: 44882950 - 45075119:1	16.53 %	12.07 %

5.3 Genome Information

Genome Version	Chr	Location	Size	Orientation	Database
GRCm39	Chr6	120,341,060-120,421,535	80,476 bases	Plus strand	NCBI
GRCm38	Chr6	120,364,099-120,444,574	80,476 bases	Plus strand	NCBI
GRCm39	Chr6	120,341,085-120,421,535	80,451 bases	Plus strand	Ensembl

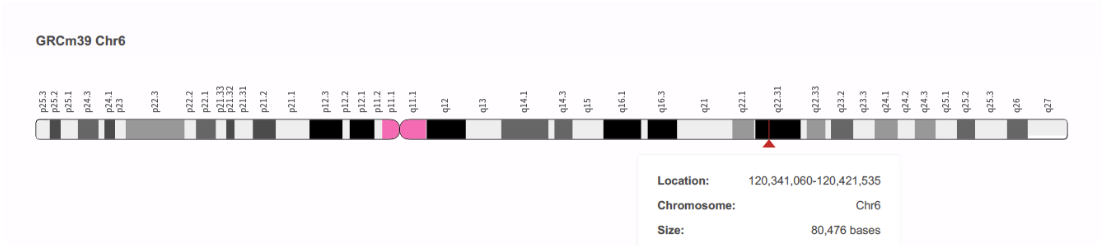

5.4 Transcript

This gene has 9 transcripts (splice variants), 222 orthologues, 8 paralogues and is associated with 11 phenotypes.

Transcript ID	Name	bp	Protein	Biotype	CCDS	UniProt Match	Flags
ENSMUST00000005108.10	Kdm5a-201	10944	1690aa	Protein coding	CCDS51889	Q3UXZ9	Ensembl Canonical, GENCODE basic, APPRIS P1, TSL:5,
ENSMUST00000203373.2	Kdm5a-209	460	153aa	Protein coding		A0A0N4SUK6	TSL:3, CDS 5' and 3' incomplete,
ENSMUST00000135802.8	Kdm5a-206	3665	814aa	Nonsense mediated decay		A0A0N4SVA7	TSL:1,
ENSMUST00000132009.4	Kdm5a-204	3329	621aa	Nonsense mediated decay		Q921T5	TSL:1,
ENSMUST00000142791.2	Kdm5a-207	1295	No protein	Protein coding CDS not defined		-	TSL:3,
ENSMUST00000124847.2	Kdm5a-203	2716	No protein	Retained intron		-	TSL:1,
ENSMUST00000152293.6	Kdm5a-208	1402	No protein	Retained intron		-	TSL:1,
ENSMUST00000124525.2	Kdm5a-202	999	No protein	Retained intron		-	TSL:5,
ENSMUST00000132599.2	Kdm5a-205	689	No protein	Retained intron		-	TSL:2,

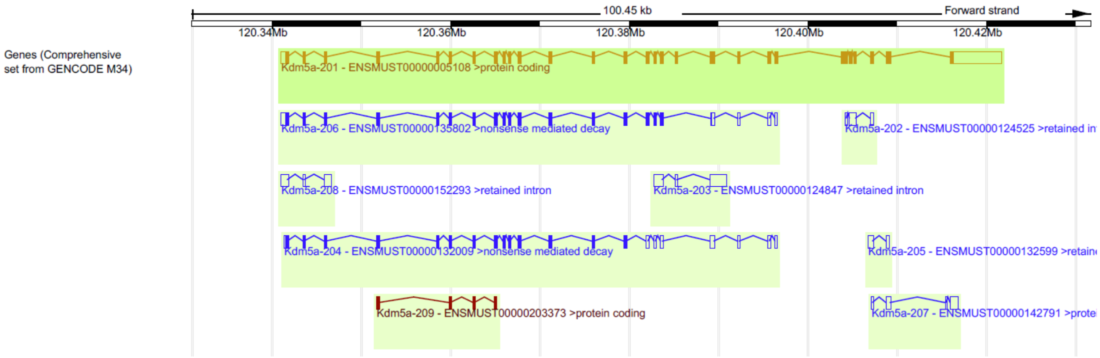

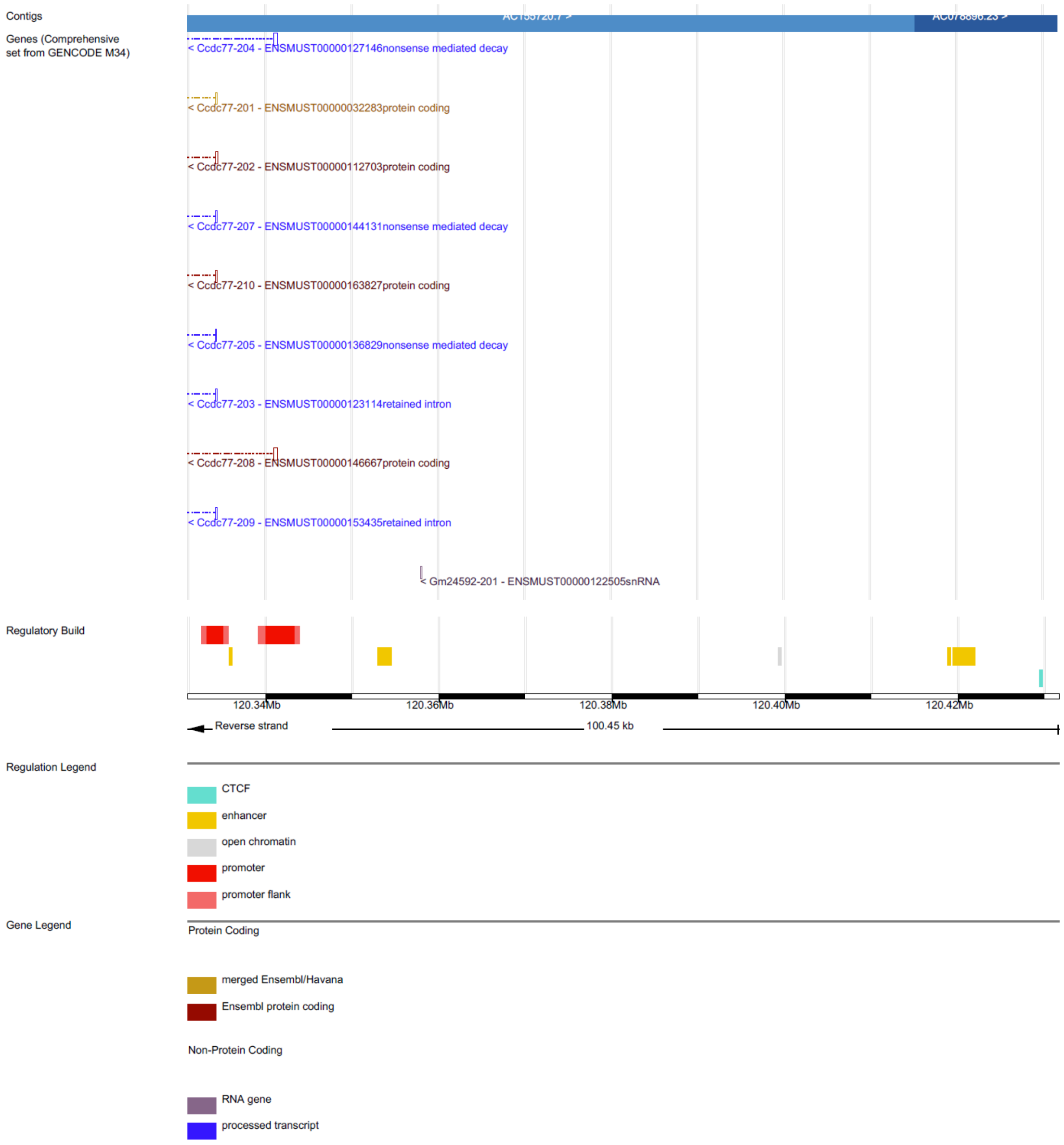

We wish to acknowledge the following scientific resources: Ensembl, RDDC, NCBI, UCSC and MGI.

5.5 Protein Function

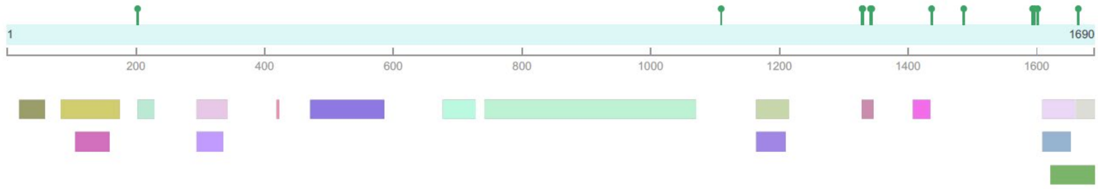

Marker	Type	Range	Feature
	Region	18 - 59	Jmjn
	Region	84 - 175	Arid_kdm5a
	Region	203 - 229	Disordered.
	Region	295 - 343	Phd1_kdm5a
	Region	419 - 423	Gsgfp motif.
	Region	470 - 586	Jmjc
	Region	676 - 728	Zf-c5hc2
	Region	741 - 1070	Plu-1
	Region	1163 - 1215	Phd2_kdm5a
	Region	1327 - 1346	Disordered.

5.6 Gene Expression

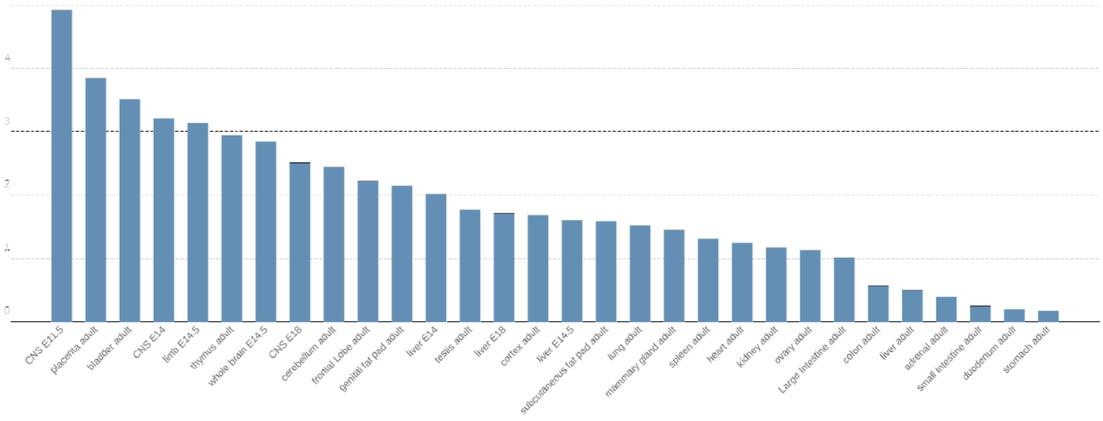

Supplementary Material 4

Tek-Cre Animal Report

Primers for Tek-Cre PCR:
Tek-Cre-F (F1): 5'-AAAAATCAGCATTTTCAACAAA-3'
Tek-Cre-R (R1): 5'-GTTTATTTACCGCCGTGTGTG-3'

Expected PCR Product:
Wildtype: N.A.
Targeted: 179 bp

Primers for Wildtype PCR:
Tek-Cre-F (F1): 5'-AAAAATCAGCATTTTCAACAAA-3'
Tek-Cre-WR (R2): 5'-TTGGATTTTAGTCCCCTATCTGA-3'

Expected PCR Product:
Wildtype: 375 bp
Targeted: N.A.

Reaction Mix:

Component	x1
Mouse genomic DNA	1.5 µl
Forward primer (10 µM)	1.0 µl
Reverse primer (10 µM)	1.0 µl
Premix Taq Polymerase	12.5 µl
ddH_2O	9.0 µl
Total	25.0 µl

Cycling Condition:

Step	Temp.	Time	Cycles
Initial denaturation	94 °C	3 min	
Denaturation	94 °C	30s	38 x
Annealing	60 °C	35s	
Extension	72 °C	35s	
Additional extension	72 °C	5 min	

Marker

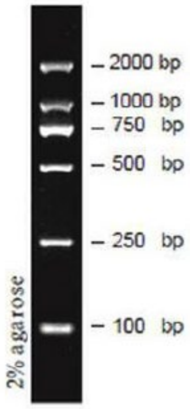

Tek-Cre PCR (WT: N.A.; MT: 179 bp)

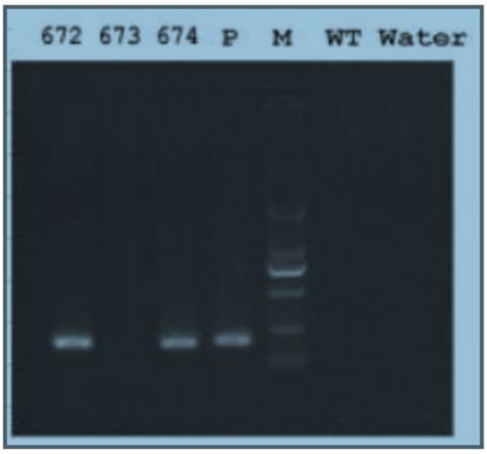

Wildtype PCR, (WT: 375 bp; MT: N.A.)

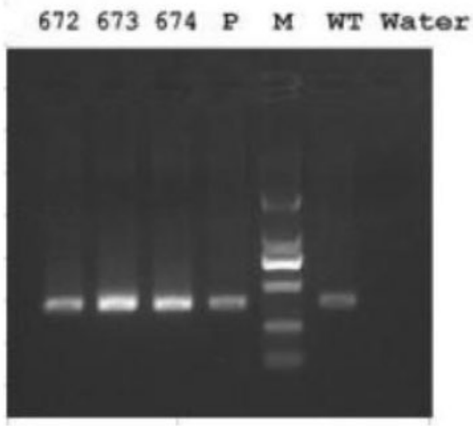

Supplement: Supplementary file 2 — Supporting Information [file ADVS-13-e12657-s002.zip › Supplementary Materials 1-4 for publication.pdf]
